# Sucrose metabolism gene families and their biological functions

**DOI:** 10.1038/srep17583

**Published:** 2015-11-30

**Authors:** Shu-Ye Jiang, Yun-Hua Chi, Ji-Zhou Wang, Jun-Xia Zhou, Yan-Song Cheng, Bao-Lan Zhang, Ali Ma, Jeevanandam Vanitha, Srinivasan Ramachandran

**Affiliations:** 1 Genome Structural Biology Group, Temasek Life Sciences Laboratory, National University of Singapore, Singapore 117604; 2 TLL-IOB Joint R&D Laboratory, Institute of Botany, Chinese Academy of Sciences, Beijing 100093, China

## Abstract

Sucrose, as the main product of photosynthesis, plays crucial roles in plant development. Although studies on general metabolism pathway were well documented, less information is available on the genome-wide identification of these genes, their expansion and evolutionary history as well as their biological functions. We focused on four sucrose metabolism related gene families including sucrose synthase, sucrose phosphate synthase, sucrose phosphate phosphatase and UDP-glucose pyrophosphorylase. These gene families exhibited different expansion and evolutionary history as their host genomes experienced differentiated rates of the whole genome duplication, tandem and segmental duplication, or mobile element mediated gene gain and loss. They were evolutionarily conserved under purifying selection among species and expression divergence played important roles for gene survival after expansion. However, we have detected recent positive selection during intra-species divergence. Overexpression of 15 sorghum genes in Arabidopsis revealed their roles in biomass accumulation, flowering time control, seed germination and response to high salinity and sugar stresses. Our studies uncovered the molecular mechanisms of gene expansion and evolution and also provided new insight into the role of positive selection in intra-species divergence. Overexpression data revealed novel biological functions of these genes in flowering time control and seed germination under normal and stress conditions.

Sucrose is the principal product of photosynthesis and plays crucial roles in growth, development, storage, signal transduction and acclimation to environmental stresses in plants. The general pathway for sucrose metabolism in higher plants is now clear and the pathway is highlighted in Supplementary Figure S1. Briefly, triose phosphates produced in the chloroplast through the Calvin cycle are transported into the cytosol, from which fructose 6-phosphates are synthesized. The products are used to produce glucose 6-phosphates and subsequently glucose 1-phosphates by phosphoglucose isomerase and phosphoglucomutase, respectively. UDP-glucose Pyrophosphorylase (UDPGP) is used to catalyze glucose 1-phosphates into UDP-glucoses, from which sucrose 6-phosphate are produced by sucrose phosphate synthase (SPS). Finally, sucrose are synthesized from sucrose 6-phosphate by sucrose phosphate phosphatase (SPP). On the other hand, sucrose is also degraded by either sucrose synthase (SuSy) or invertase. Under SuSy, sucrose is degraded into UDP-glucose, which is a reversible reaction. Under invertase, sucrose is inverted into both fructose and glucose. The former is used to synthesize fructose 6-phosphate by fructokinase and the latter is phosphatized into glucose 6-phosphte under hexokinase. Thus, at least 9 enzymes have been involved in sucrose metabolism, which are fructokinase, hexokinase, invertase, phosphoglucomutase, phosphoglucose isomerase, SuSy, SPS, SPP and UDPGP. We are more interested in the last four types of enzymes as they might contribute more to the synthesis of sucrose rather than degradation when compared with the remaining 5 types of enzymes.

*SuSy* members are encoded by a small multiple gene family. With the release of increasing whole genome sequencing data, more and more *SuSy* gene families were genome-widely identified and characterized in plants. Both model plants Arabidopsis and rice encode 6 *SuSy* genes^1^,^2^ whereas 7 *SuSy* members were identified in either poplar or cotton^3^,^4^. Currently available data show that higher plants encode 4-15 SuSy genes^5^,^6^,^7^. However, compared with the *SuSy* family, less information is available for the genome-wide identification and characterization as well as evolution of genes encoding SPS, SPP and UDPGP. Both SPS and SPP are also encoded by small gene families. SPS is a key enzyme for sucrose synthesis. Castleden *et al.* (2004) carried out phylogenetic analysis based on all available SPS sequences at that time^8^. Subsequently, genomic and EST sequence data from Arabidopsis, rice and maize were employed to identify the *SPS* genes. The analysis showed that the Arabidopsis and rice genomes encode a total of 4 and 5 members, respectively and gene duplication might play a role in their expansion^8^,^9^,^10^. Relatively, fewer reports have been focused on *SPPs*. Genes have been identified from several species including rice, maize, wheat, barley, Arabidopsis and tomato^11^,^12^. These species contain at least two *SPP* genes^12^,^13^,^14^. For example, four and three *SPP* genes have been identified in Arabidopsis and rice, respectively^11^. For the *UDPGP* families, previous data showed that only one or two *UDPGPs* existed in plants^15^. However, studies showed that UDP-sugar pyrophosphorylase also catalyzed the same reaction as the enzyme has broad substrates specificity^16^ (Litterer *et al.* 2006). Besides UDP-glucose, it also catalyzes the reversible formation of UDP-galactose, UDP-glucuronic acid, UDP-l-arabinose and UDP-xylose. In addition, these types of enzymes share the same protein domain structure. Thus, in this study, UDPGP refers to these enzymes with the common Pfam domain (PF01704, http://pfam.xfam.org/) structure. Although more and more whole genome sequencing data are available, no genome-wide identification and characterization have been recently reported for *SPS*, *SPP* and *UDPGP*. In addition, the evolutionary analysis on *SuSy*, *SPS* and *SPP* and *UDPGP* were mainly focus on the investigation among different genus, families, orders and even kingdoms^3^,^6^,^8^,^12^,^15^,^17^ and little is known on the recent evolution among species, subspecies or varieties belonging to the same species. *SuSy*, *SPS* and *SPP* could be detected in plants and bacteria. However, *UDPGP* were detected not only in plants and bacteria but also in animals and fungi. Furthermore, all four types of enzymes have been undergone gene duplication but the mechanism is not yet elucidated.

On the other hand, functional characterization of these four gene families has also been carried out in some plant species by overexpression, knockout and silencing of genes. Overexpression of a *SuSy* gene increased biomass production and/or sucrose content in switchgrass, tobacco, cotton or poplar^18^,^19^,^20^,^21^. Loss of function of a *SuSy* gene by knockout mutation exhibited various visible phenotypes^22^,^23^,^24^. However, no obvious difference in sucrose synthase activity or growth phenotype was observed when one of two *SuSy* genes was knocked out in Arabidopsis^25^. Overexpression of *SPS* genes significantly increased sucrose synthesis in certain tissues and led to the changes in phenotypes including increased biomass^26^,^27^,^28^. Anti-sense expression of *SPS* genes or knockout of these genes by T-DNA/retrotransposon insertion repressed sucrose synthesis and resulted in abnormal development^29^,^30^. Decreased SPP level in transgenic tobacco by RNAi inhibited photosynthesis and altered carbohydrate partitioning with reduced growth rate. SPP activity was also repressed in transgenic potato tubers (*Solanum tuberosum*) by RNAi, which affected the hexose-to-sucrose ratio upon cold storage with only minor effects on total soluble carbohydrate accumulation^14^.

Antisense suppression of a *UDPGP* gene in plants could reduce soluble carbohydrate and starch or sucrose content^31^,^32^. In rice, inactivation of the *UGPase1* gene led to genic male sterility^33^,^34^. Investigation of T-DNA insertion of a *UDPGP* gene suggested it should be a novel plant cell death regulator^35^. Data also showed that overexpression of a native or foreign *UDPGP* gene in various plant species could increase plant height^18^ and enhance leaf area and leaf-to-shoot biomass ratio^36^ or vegetative growth^37^. Generally, limited data are available for studies on biological functions of these four gene families. For some important crops, for example, sweet sorghum, little is known about their biological functions of these four gene families although sucrose metabolism is an important step for stem sugar accumulation in this plant.

In this study, we first genome-widely identified and characterized all genes encoding SuSy, SPS, SPP and UDPGP in 15 species from algae, moss as well as monocot and dicot plants. We then evaluated their expansion mechanisms and evolutionary history. Subsequently, we investigated their selection forces and expression divergence among different tissues or under various stresses to further annotate their biological functions and retention mechanisms of expanded genes. Finally, we investigated their biological functions by overexpressing 15 sorghum genes in the Arabidopsis genome. Our data show that plant species encode different numbers of *SuSy*, *SPS*, *SPP* and *UDPGP* genes. These four gene families exhibited different expansion and evolutionary history as their host genomes experienced differentiated rates of the whole genome duplication, tandem and segmental duplication, or mobile element mediated gene gain and loss. All these were evolutionarily conserved under purifying selection among species and expression divergence played important roles for gene survival after expansion. However, we have detected recent positive selection in several sites of the sorghum *UDPGP* gene *Sobic.006G213100*, suggesting a role of this gene in intra-species divergence. Overexpression of 15 sorghum genes in Arabidopsis showed the roles in biomass accumulation and revealed novel biological functions in seed germination under normal and high salinity stress.

## Results

### Plant genomes encode different sizes of the *SuSy*, *SPS*, *SPP* and *UDPGP* families

To identify and characterize the four family members on a genome-wide level, 15 completely sequenced genomes from monocot, dicot plants as well as algae and spikemoss have been selected for such analyses as listed in Table 1. The Hidden Markov model (HMM) searches followed by subsequent confirmation showed that not all plant genomes encoded the all four gene families (Table 1). Generally, all the 15 selected plant genomes have contained three gene families including *SuSy*, *SPP* and *UDPGP*. However, no *SPS* gene has been detected in the lower plant species *C. reinhardtii*. For the *SuSy* gene family, only one gene was detected in both *C. reinhardtii* (alga) and *S. moellendorffii* (moss) and most species encode more than 4 *SuSy* genes with the highest number (31) of *SuSy* genes in apple (Table 1). For the *SPS* gene family, most of plants encode 3-5 members whereas up to 7 *SPS* genes were identified in maize, soybean or apple (Table 1). For the *SPP* gene family, only one gene was detected in monkey flower and castor bean and the remaining genomes encode 2-5 genes. For the *UGPGP* gene family, the plant genomes encode at least 3 members whereas up to 15 members were detected in the soybean genome. Besides the members as listed in Table 1, we have also identified numbers of members encoding partial domains of these protein families. Further analysis showed that these members contained no typical domain structure and they also lacked expression evidence. Thus, these members were removed from our further analysis due to the low feasibility of phylogenetic analyses although the rate of gene duplication might be under-estimated.

### Four gene families exhibited different expansion and evolutionary history

**Figure 1:**
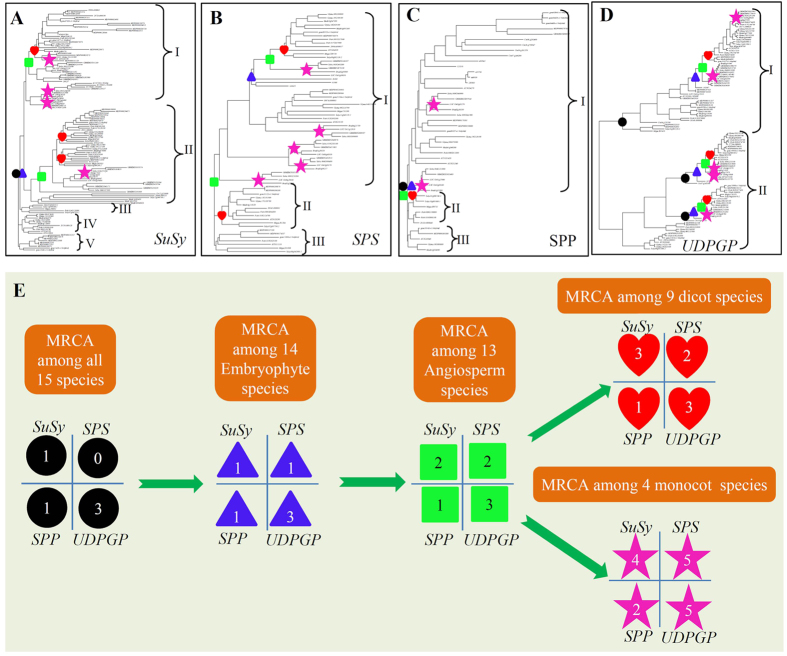
Phylogenetic analyses and evolutionary history of the *SuSy*, *SPS*, *SPP* and *UDPGP* families. (**A–D**) Phylogenetic analyses of the *SuSy*, *SPS*, *SPP* and *UDPGP* families, respectively, in 15 species including 4 monocot and 9 dicot plants as well as 1 spikemoss and 1 algae. Domain amino acid sequences were achieved to construct phylogenetic trees using the bootstrap method with a heuristic search of the PAUP 4.0 b8 program. The results were confirmed by the Bayesian analyses. We defined ancestral units according to the description by Shiu *et al.* (2004)^38^. Their enlarged phylogenetic trees are shown in Supplementary Fig. S2 (**A–D**), respectively. **(E)** Evolutionary history of the four gene families in 15 organisms. Black circles represent the MRCA units among all 15 organisms; blue triangles indicate the MRCA units among 14 embryophyte species and green squares show the MRCA units among angiosperm species. Red heart shapes and pink stars represent the MRCA units in 9 dicot and 4 monocot plants, respectively.

To classify the members of these four gene families and to facilitate their functional analysis, we reconstructed the phylogenetic relationship in each family (Fig. 1A–D, Supplementary Fig. S2 A–D) using their corresponding protein domain sequences as described in the Methods. Previous studies identified 3 groups of *SuSy* genes, which were based on available *SuSy* members from limited plant species^4^,^5^. However, the phylogenetic tree based on *SuSy* members from 15 species showed that a total of 5 groups could be clustered (Fig. 1A and Supplementary Fig. S2A). Both groups I and II are the main groups consisting of members from all dicot and monocot plants. The remaining three groups are species-specific and they consist of members from limited species. For the *SPS* gene family, we identified 3 groups (Fig. 1B and Supplementary Fig. S2B). Among them, group I is the largest one, consisting of the all members from both dicot and monocot plants. Previous data showed that four groups were identified^8^. However, in the study, groups B and C were actually in the same group^8^; thus, similar to our result. On the other hand, the *SPP* family was also clustered into three different groups with the group I as the largest one consisting of all members from angiosperm species (Fig. 1C and Supplementary Fig. S2C). As for the *UDPGP* family, no genome-wide identification was previously reported. We have identified a total of 110 members from 15 species (Table 1) and they could be clustered into two groups (Fig. 1D and Supplementary Fig. S2D). In each group, at least one member has been included from all the 15 species.

**Figure 2:**
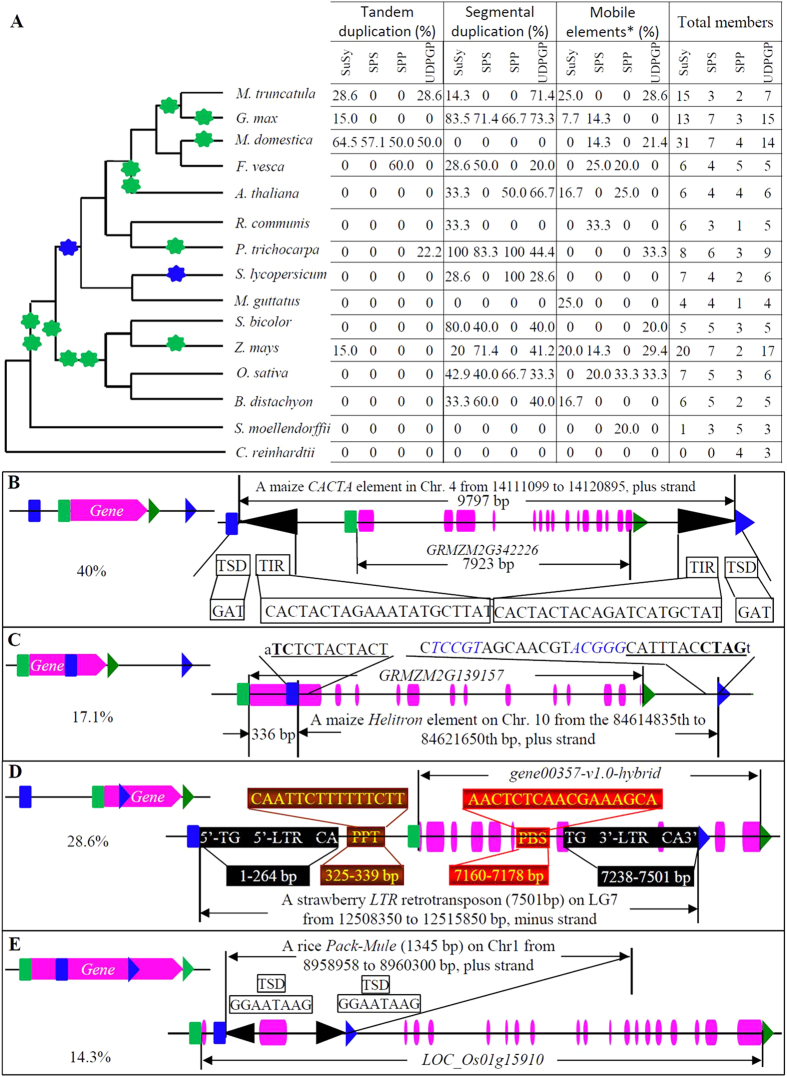
Expansion mechanisms of the *SuSy*, *SPS*, *SPP* and *UDPGP* families in 15 plants. **(A)** The phylogenetic tree in left panel was constructed according to the plant genome duplication database (http://chibba.agtec.uga.edu/duplication/) and showed the whole genome duplication history of 15 species. Green and blue stars indicate whole genome duplication and triplication, respectively. Columns in right panel in **(A)** indicates the contributions of tandem, segmental duplications and mobile elements to the expansion of the *SuSy*, *SPS*, *SPP* and *UDPGP* families in 15 genomes. The last column showed the summary of all identified family members in 15 species. The star “*” indicates mobile elements including *LTR*-retrotransposon, retrogene, *Pack*-*Mule*, *hAT*, *Helitron* and *CACTA* elements. Figures **(B–E**) show different types of expansion of family members by transposition. **(B)** A whole gene fragment was duplicated (type 1). A maize gene *GRMZM2G342226* was expanded by a *CACTA* element in chromosome 4, which was characterized with typical *CACTA* structures. TSD, target site duplication; TIR, terminal inverted repeat. **(C)** Only 3′-region of a gene was duplicated (type 2). The 3′-region of a maize gene *GRMZM2G139157* was duplicated by the *Helitron* element on chromosome 10 with typical *Helitron* features. The *Helitron* begins with “TC” and ends with CTAG, which were conserved sequence motifs (indicated by bold uppercase letters). These two motifs form parts of 11 bp palindromic sequences (underlined). *Helitron* sequences are in uppercase letters and the invariant host nucleotides where the *Helitrons* insert are in lowercase letters. The inverted repeats at the 3′ termini are in blue fonts. The 336 bp fragment is the duplicated gene fragment. **(D)** The 5′-region of a gene was duplicated (type 3). The strawberry gene *gene00357-v1.0-hybrid* was duplicated by a *LTR*-retrotransposon. The figure indicates structural features of this retrotransposon including 5′- and 3′-*LTR*, both of which start with TG and end with CA. Both PPT (polypurine tract) and PBS (primer binding site) are also indicated. **(E)** The middle region of a gene was duplicated (type 4). The middle region of a rice gene *LOC_Os01g15910* was duplicated by a rice Pack-Mule on chromosome 1. TSD, target site duplication.

To evaluate the patterns of expansion and evolutionary history of these four gene families, we broke down the phylogeny tree into ancestral units and estimated the most recent common ancestor (MRCA) among species according to the method described by Shiu *et al.* (2004)^38^. Due to possible gene losses and pseudogenes, which were excluded in the phylogenetic trees, the MRCA members may be under-estimated. Only one gene with locus name *Cre12.g524000* was identified in the green algae species *C. reinhardtii* and the MRCA among the 15 species was estimated as 1 (Fig. 1E). Since no member was detected in the algae species for the *SPS* family, no MRCA exit among analyzed 15 species (Fig. 1E). The MRCA among 14 embryophyte species still had only 1 gene encoding SuSy and SPS, respectively. One more gene was expanded in the MRCA among 13 angiosperm species. During the divergence between monocot and dicot plants, the expansion rate varied. More *SuSy* and *SPS* genes were required for monocot plants when compared with dicot plants. The MRCA among 4 monocot plants was estimated at 4 and 5 members for *SuSy* and *SPS*, respectively whereas only 3 *SuSy* and 2 *SPS* genes were detected in the MRCA among 9 dicot plants (Fig. 1E). We further surveyed the remaining two families including *SPP* and *UDPGP*. Both *SPP* and *UDPGP* are ancient families with at least 1 and 3 members at the MRCA among 15 organisms (Fig. 1E). Following this era, no expansion occurred until the divergence between dicot and monocot plants. After divergence between dicot and monocot plants, no expansion was detected in dicot plants. However, in monocot plants, additional one for *SPP* and two for *UDPGP* members were required, making a total of 2 *SPP* and 5 *UDPGP* members in the MRCA among the monocot species. These data demonstrated that both monocot and dicot plants experienced differences in their expansion history for these gene families. Interestingly, the comparison of the general phylogenetic tree of 15 species and their encoded *SuSy*, *SPS*, *SPP* and *UDPGP* genes also suggested that these species experienced a differentiated family expansion history (Fig. 2A). A relatively large scale of gene expansion should have occurred for some species during species divergence from the MRCA among either monocot or dicot plants. The examples include the expansion of *SuSy* and *UDPGP* genes from maize, barrel medic, soybean and apple (Table 1 and Fig. 2A). In apple (*M. domestica*), a total of 31 *SuSy*, 7 *SPS*, 4 *SPP* and 14 *UDPGP* genes were detected with at least triple times of expansion from the MRCA among dicot plants. In its closely related species *F. vesca* (strawberry), only 6 *SuSy*, 4 *SPS*, 5 *SPP* and 5 *UDPGP* genes were encoded (Fig. 2A). The data suggested that a large scale of expansion occurred after the divergence of apple from strawberry. A similar situation was also observed in the maize species. However, for soybean and barrel medic (*M. truncatula*), the *SuSy* gene expansion might occur before the divergence of these two species and the whole-genome duplication might contribute to the expansion (Fig. 2A). On the other hand, our data also showed that most of species required only small families of *SuSy*, *SPS*, *SPP* and *UDPGP* with less than 10 members each.

### Contributions of duplication and transposition to family size

To explore the mechanisms of these four gene family expansion, we investigated the contributions of various gene expansion ways to the family size. These mechanisms include tandem, segmental and the whole genome duplication, transposition and retrotransposintion. We first surveyed the contribution of tandem duplication to the gene expansion. We examined the physical positions of the family members on corresponding genome chromosomes. The results showed that tandem duplication contributed to the family expansion in 6 of 15 species (Fig. 2A). It mainly contributed to the expansion of both *SuSy* and *UDPGP* gene families. However, in the apple genome, it was the main way for the expansion of all four gene families. More than 50% of *SuSy*, *SPS*, *SPP* and *UDPGP* genes were expanded by tandem duplication. We then analyzed the contribution of segmental duplication to the family expansion. Our data showed that segmental duplication significantly contributed to the expansion of *SuSy*, *SPS*, *SPP* or *UDPGP* genes in at least 11 species (Fig. 2A). The contribution rates varied depending on species and their encoded gene families, ie. *SuSy*, *SPS*, *SPP* or *UDPGP*. In *R. communis* and *M. truncatula*, only one and two out of the four gene families was expanded by segmental duplication, respectively. For the remaining species, segmental duplication played roles in the expansion of three or four gene families. In some gene families, majority of their expansion was due to segmental duplication. For example, 100% and 83.5% of *SuSy* genes were expanded by segmental duplication in poplar and soybean, respectively. Besides tandem and segmental duplication, we also surveyed the contribution of the whole genome duplication to the family expansion. Due to the difficulty in distinguishing segmental duplication from the whole-genome duplication followed by massive loss of duplicate genes^39^, we compared the events of genome doubling (green stars in Fig. 2A) or tripling (blue stars in Fig. 2A) with the family size of *SuSy*, *SPS*, *SPP* and *UDPGP*. The comparison implied that genome doubling or tripling also contributed to the expansion of these four gene families in some species. For example, *Z. mays* (maize) underwent one more genome doubling event when compared with *S. bicolor* (sorghum) and as a result, the maize genome encoded more members of *SuSy*, *SPS* and *UDPGP* (Fig. 2A).

On the other hand, we have also investigated the contribution of transposable elements (TEs) to the expansion of these four gene families. We have analyzed the roles of *LTR*-retrotransposon, *retrogene*, *Pack*-*Mule*, *hAT*, *Helitron* and *CACTA* elements in the expansion of the four gene families. Generally, TE-mediated family expansion has been detected in 13 out of 15 species (Fig. 2A). However, only one to four genes were expanded by mobile elements in each species for each gene family. Thus, TE-mediated expansion might not contribute to the large scale of family expansion. Further investigation showed that there exited 4 types of TE-mediated gene expansions as shown in Fig. 2B–D. In type 1, a whole gene fragment was duplicated and it accounted for around 40% of TE-expanded genes. For example, a 9797 bp long maize *CACTA* element carried a 7923 bp gene *GRMZM2G342226* (Fig. 2B), which encodes a UDPGP protein. In type 2, only 3′-region of a gene was duplicated by a TE, accounting for 17.1% of TE-related genes (Fig. 2C). An example is the maize *Helitron* element, which carried a 3′-region of the *SuSy* gene *GRMZM2G139157*. This 3′-region encodes an intact SuSy domain. In contrast, in type 3, 5′-region of a gene was duplicated by a TE and around 28.6% of TE-related genes were expanded by this type (Fig. 2D). For example, a strawberry *LTR* retrotransposon carried a 5′-region of the gene *gene00357-v1.0-hybrid*, which encodes a SPP protein. In type 4, a middle region of a gene was duplicated by a TE and up to 14.3% of TE-related genes were expanded by this type (Fig. 2E). An example is the rice *PACK*-*MULE* on chromosome 1, which carried a fragment of a *UDPGP* gene with the locus name *LOC*_*Os01g15910*.

### Different selection forces between monocot and dicot plants for the *SPP* family

**Figure 3:**
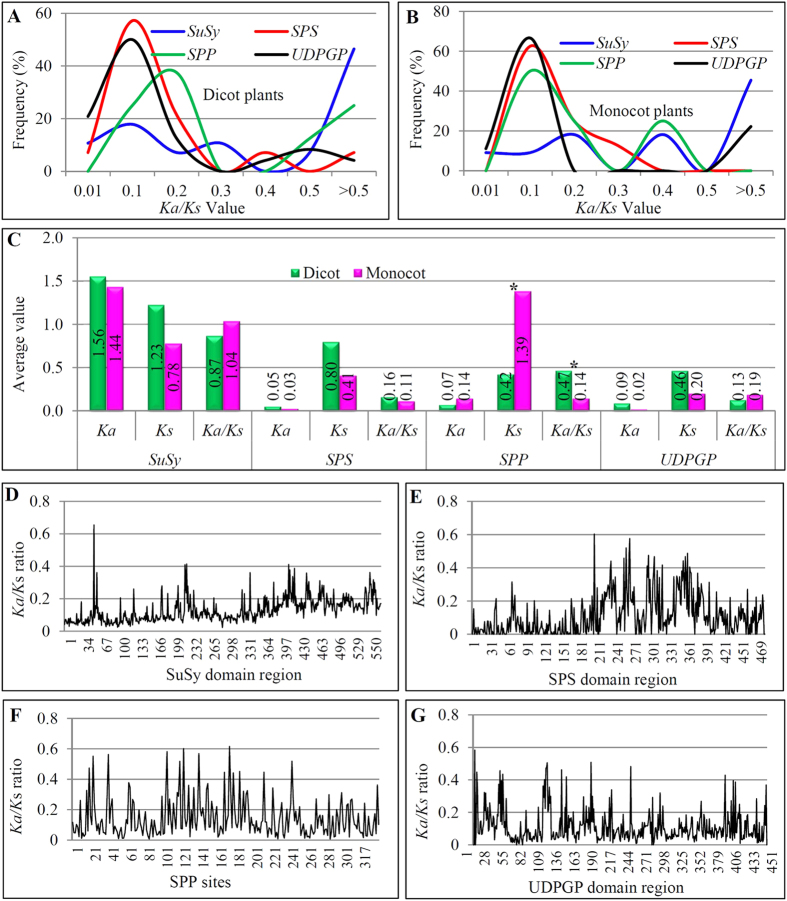
Frequency distributions of *Ka*/*Ks* ratios between expanded gene pairs and tests of sites with purifying/positive selection in the *SuSy*, *SPS*, *SPP* and *UDPGP* families. (**A,B**) Frequency distributions of *Ka*/*Ks* ratios were analyzed using expanded pairs from dicot and monocot plants, respectively. (**C**) The average *Ka*, *Ks* and their ratios in monocot and dicot pairs. Asterisks indicate significant differences between monocot and dicot plants at P, 0.05 (*) by *t*-test. (**D–G**) Screening for amino acid sites with purifying/positive selection in the four gene families by the SLR program as described in the Methods.

Since the distinct difference was observed in their expansion history among the four gene families or in the same family between dicot and monocot plants (Figs 1 and 2), we investigated whether they were under different selection forces. We first identified reciprocal best matches for all four gene family members either from dicot or monocot plants by phylogenetic analysis using corresponding domain regions. The domain regions from these identified matches were then used to calculate their *Ka* (nonsynonymous substitutions per site), *Ks* (synonymous substitutions per site) and their ratios according to the description in Methods. We first analyzed and compared the *Ka*/*Ks* frequency distribution of these four gene families between monocot and dicot plants (Fig. 3A,B). For the *SuSy*, *SPS* and *UDPGP* families, similar frequency distributions of the *Ka*/*Ks* ratios were observed between monocot and dicot plants although their distribution patterns showed differences. There was also no significant difference between monocot and dicot plants in the average *Ka*, *Ks* and *Ka*/*Ks* values for these three gene families (Fig. 3C). For the *SuSy* family, most of the mass were centered near *Ka*/*Ks* > 0.5 whereas most of the mass for both *SPS* and *UDPGP* were centered near *Ka*/*Ks* = 0.1. Furthermore, the *SuSy* family also has the higher average *Ka*/*Ks* ratios at 0.87 for dicot and 1.04 for monocot plants whereas the average ratios for both *SPS* and *UDPGP* families were less than 0.2 for either dicot or monocot plants (Fig. 3C). These data suggested that the *SuSy* family evolved faster than the *SPS* and *UDPGP* families. However, for the *SPP* family, significant difference was observed between monocot and dicot plants in their *Ka*/*Ks* frequency distribution (Fig. 3A,B). In dicot plants, most of the mass was centered near *Ka*/*Ks* = 0.2 whereas the peak was near 0.1 for monocot plants. The average *Ka*/*Ks* for dicot plants was 0.47, statistically higher than the ratio 0.14, which was calculated for monocot plants (Fig. 3C). To further analyze the reason why both dicot and monocot plants exhibited the difference in their evolutionary rate, we compared their *Ka* and *Ks* values separately. We found that the *Ka* value in monocots is twice the value in dicot plants whereas the *Ks* value in monocots is more than three times higher than that in dicots (Fig. 3C). As a result, dicots showed significantly higher *Ka*/*Ks* ratio than that in monocots. Thus, the *SPP* family in dicot plants evolved faster than that in monocot plants.

After analyzing the *Ka*/*Ks* ratios between the orthologous pairs, we further surveyed the selection forces in each amino acid site of corresponding domain regions of four gene families among 15 species. We submitted both aligned cDNA sequences deduced from domain amino acids and their phylogenetic tree to the sitewise likelihood-ratio (SLR) analysis^40^. For the *SuSy* family, *Ka*/*Ks* ratios in each site of the domain regions among tested 15 species ranged from 0.02 to 0.65 and no site showed the ratio higher than 1 (Fig. 3D). Similar results were observed for the *SPS* (Fig. 3E), *SPP* (Fig. 3F) and *UDPGP* (Fig. 3G) families. The data suggested that all domain amino acid sites from the four gene families were unusually conserved and were subjected to purifying selection.

### Rapid gene expansion and expression divergence

**Figure 4:**
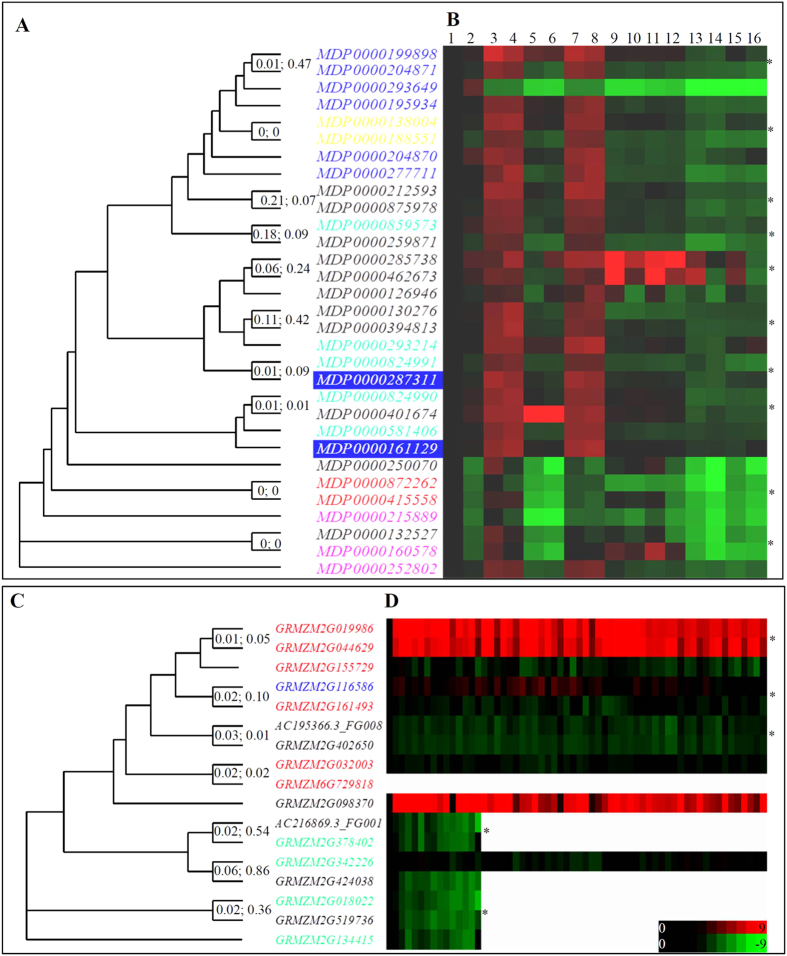
Expression patterns and divergence of both apple *SuSy* and maize *UDPGP* gene families among various tissues. (**A**) The phylogenetic tree of the *SuSy* family in apple. The apple family was expanded only by tandem duplication. Locus names with black fonts indicate that these genes were not duplicated. A total of 6 tandem clusters were identified and they were labelled with blue, brown, green, white with blue background, red and pink colors. **(B)** The expression profiling of this gene family among 16 tissues. Expression data were downloaded from the NCBI datasets (Methods) with accession number GSE42873. These issues were listed as below: 1, Flower_M67; 2, Fruit_M74_100 daa; 3, Flower_M74; 4, Fruit_M20_100 daa; 5, Leaf_M14; 6, Fruit_M20_harvest; 7, leaf_M49; 8, Fruit_M74_harvest; 9, Root_GD; 10, Stem_X8877; 11, Root_X8877; 12, Stem_GD; 13, Seedling_GD; 14, Seed_X4442 × X2596; 15, Seedling_X4102; and 16, Seed_X3069 × X922. **(C)** The phylogenetic tree of the *UDPGP* family in maize. Locus names with red and green fonts indicate that they were from segmental duplication and TE-mediated expansion, respectively. The locus name with blue fonts indicates that the gene was expanded by TE but was located in a segmentally duplicated block. **(D)** The expression analysis of two datasets, which were downloaded from the NCBI datasets with accession numbers GSE27004 and SRA012297. These two datasets consisted of 60 and 15 tissues, respectively and their names were listed in their databases. In (**A,B**) both *Ka* values and *Ka*/*Ks* ratios for these nodes between closely related genes were calculated and were shown in the left and right of semicolon of the node. Normalized expression values were calculated by Agilent GeneSpring GX 11.5 software and were used for heat map analyses as shown in the right panel. Red, black, and green colors indicated the normalized expression values with >0, = 0, and < 0, respectively, in the matrix. The stars “*” indicated the statistical difference in their expression level between these two genes.

Our data showed that the *SuSy*, *SPS*, *SPP* and *UDPGP* families were evolutionarily conserved under purifying selection (Fig. 3). Our data also showed that gene expansion occurred in all the four gene families (Fig. 2). How did these expanded genes survive with limited functional divergence? We further investigated the expression profiling of these genes. We selected two gene families for these analyses. One of them is the *SuSy* family from the apple genome and another one is the *UDPGP* family from maize. The *SuSy* family underwent the rapid expansion during the divergence of *M. domestica* from *F. vesca*. In *F. vesca*, a total of 6 members were identified whereas up to 31 members were annotated in the apple genome (Fig. 2A). Tandem duplication played a key role in the expansion. After duplication, very low divergence was detected. We have selected 10 closely related pairs for *Ka*/*Ks* evaluation and the analysis showed that the *Ka*/*Ks* ratios were low and some of them had no non- synonymous substitution (Fig. 4A). Expression analysis showed that all of the 31 genes were expressed in at least one of sixteen tissues (Fig. 4B). There were a total of 6 tandem duplicated arrays as shown by different colors in Fig. 4A. Individuals from each array showed expression differentiation in at least one of sixteen detected tissues. We then analyzed the expression divergence between the 10 closely related pairs. The data showed that these pairs also exhibited expression differentiation in at least one out of sixteen tissues by statistical analysis (Fig. 4B).

Another selected family for expression analysis is the *UDPGP* family from the maize genome. The *UDPGP* family also experienced the rapid expansion during the divergence of maize from sorghum. In sorghum, only 5 genes were identified to encode *UDPGP* whereas the maize genome encodes up to 17 *UDPGP* genes. Our investigation showed that both segmental duplication and transposition/retrotransposition played important roles in the family expansion (Fig. 2). A total of 7 genes (41.2%) have been involved in segmental duplication and both *CACTA* and *LTR* retrotransposons have contributed to the expansion of 5 genes (29.4%) (Fig. 2). They were clustered into different groups (Fig. 4C). Microarray data consisting of 60 tissues and RNA_Seq data consisting of 15 tissues were employed for expression analysis. No probe is available or no RNA_seq signal was detected for the gene *GRMZM6G729818*. Expression evidence was presented for all the remaining 16 *UDPGP* genes (Fig. 4D). We also found that expression divergence was observed among segmentally duplicated genes and among *CACTA*/*LTR*-expanded genes (Fig. 4C,D). On the other hand, the phylogenetic analysis identified a total of 7 closely related pairs (Fig. 4C). Some of these pairs also showed *Ka*/*Ks* ratios as low as 0.01. However, all of these pairs exhibited significant expression divergence as shown by statistical analysis (Fig. 4D).

### Genome variations and expression profiling between grain and sweet sorghum species

**Figure 5:**
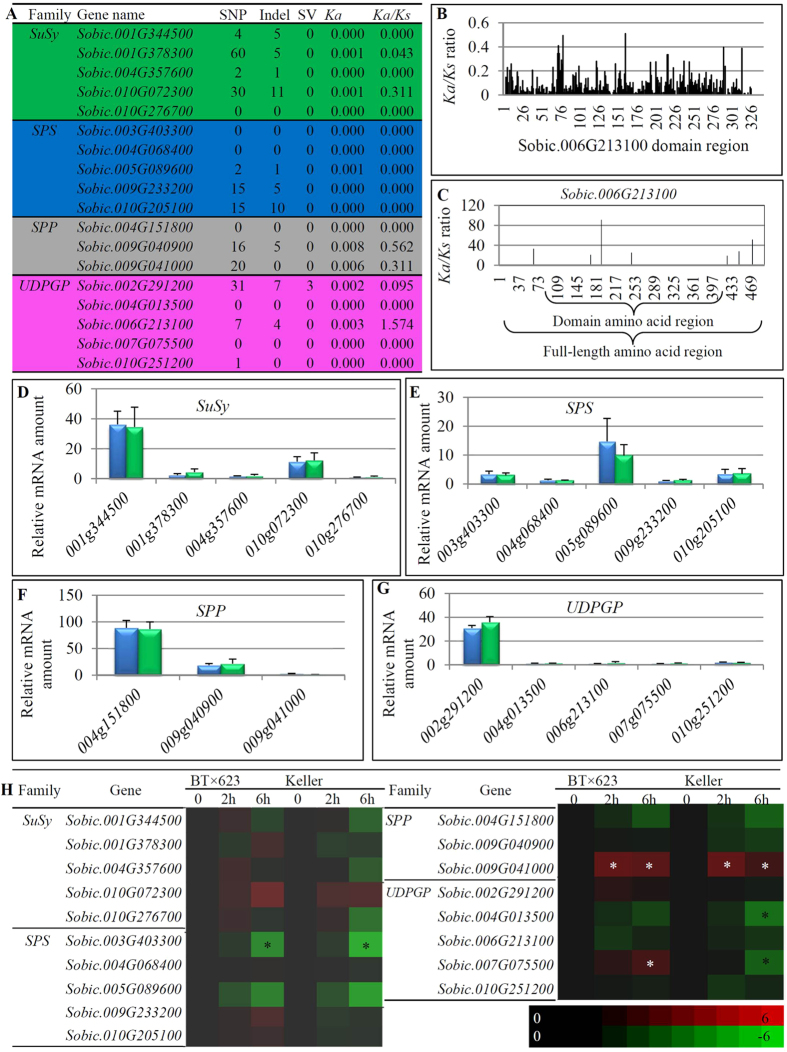
Genomic variations and expression divergence of the *SuSy*, *SPS*, *SPP* and *UDPGP* families between grain and sweet sorghum. (**A**) Detected SNP, Indel and SV between grain (BT × 623) and sweet (Keller) sorghum lines and their *Ka*/*Ks* analysis. (**B,C**) Screening for amino acid sites with purifying/positive selection for the gene *Sobic.006G213100* by the SLR program. (**B**) No positive selection was detected among 15 species (inter-species). (**C**) Positive selection was detected among 44 sorghum lines, which belong to the same species (intra-species). **(D**–**G**) Comparative expression analysis of *SuSy*, *SPS*, *SPP* and *UDPGP* family members between grain (blue column) and sweet (green column) sorghum lines, respectively, by qRT-PCR. The prefix “*Sobic*.” was omitted in each locus name. (**H**) Expression divergence of the *SuSy*, *SPS*, *SPP* and *UDPGP* gene families between grain and sweet sorghum lines under sucrose treatment. Red, black, and green colors indicated the normalized expression values with >0, = 0, and < 0, respectively, in the matrix. The stars “*” indicated the statistical difference in their expression level between these two genes at P < 0.05.

BT × 623 and Keller belong to the same species *S. bicolor*. However, they exhibited obviously different phenotypes^41^. The former is grain sorghum and the latter is sweet sorghum with higher sucrose content in its stem. Genome sequences for these two cultivars were publicly available^42^. Our HMM searches identified the same numbers of *SuSy*, *SPS*, *SPP* and *UDPGP* families in these two cultivars. Genome-wide variation analysis identified one *SuSy*, two *SPS*, one *SPP* and two *UDPGP* genes showed 100% homology in their genome sequences with no single nucleotide polymorphism (SNP), no insertion/deletion (Indel) and no structural variation (SV) (Fig. 5A). The other 11 genes contained either SNP or Indel and only one gene *Sobic.002G291200* has been detected with SV (Fig. 5A). The *Ka*/*Ks* analysis showed that up to 12 out of 18 genes from the four gene families showed no non-synonymous substitution with *Ka*/*Ks* = 0. Most of the remaining genes showed low ratios (0.043–0.562). Only one gene *Sobic.006G213100* exhibited *Ka*/*Ks* > 1 (1.574) (Fig. 5A), suggesting the positive selection for this locus in these two varieties. As our data have showed that no positive selection occurred for all the four gene families among 15 species (Fig. 3D–G), we were interested in whether the positive selection could be detected in other sorghum lines within the species. We have further confirmed that the gene *Sobic.006G213100* showed no positive selection among 15 different species (Fig. 5B). We then analyzed the selection force of this gene among 45 intra-species lines, which belong to the same species *S. bicolor*. As these lines are from the same species, we could align the full-length amino acid sequences instead of domain sequences for the *Ka*/*Ks* analysis. Our data showed that a total of 7 sites were under positive selection with *Ka*/*Ks* ratios larger than 1(P < 0.05) (Fig. 5C). Three of them were located on domain region and the remaining 4 sites were out of the domain (Fig. 5C). The *Ka*/*Ks* ratios in the remaining sites were zero with no nonsynonymous substitution. The data suggested that mutation occurred only in limited sites and positive selection of the gene *Sobic.006G213100* occurred during the divergence of intra-species.

Besides the genome divergence between BT × 623 and Keller, we also carried out the investigation of their expression profiling by quantitative reverse transcription PCR (qRT-PCR). We compared their expression abundance during seedling stage between BT × 623 and Keller (Fig. 5D–G). In each of analyzed four gene families, there was one gene which showed the most abundant expression level with at least 3 times higher than the remaining genes in that family. Further analysis showed that the highest expression level was higher than the sum of the remaining members. However, we did not observed any difference in their expression abundance at this growth stage between BT × 623 and Keller. We further analyzed the difference in expression regulation under sucrose treatment (Fig. 5H). Generally, for most of genes from these four gene families, no difference was observed between BT × 623 and Keller in their responses to sucrose stress. For example, in the *SuSy* family, no gene was detected with up- or down-regulation by sucrose treatment. In the *SPS* family, the gene *Sobic*.*003G403300* was down-regulated by sucrose in both BT × 623 and Keller. In contrast, the *SPP* gene *Sobic*.*009G041000* was up-regulated by sucrose in these two varieties. For the *UDPGP* family, two genes showed the difference in their responses to sucrose stress between these two varieties. One of them is *Sobic*.*004013500*, which was down-regulated by sucrose stress only in Keller. Another one is *Sobic*.*007G075500*, which was up-regulated by sucrose stress in BT × 623 but down-regulated in Keller (Fig. 5H).

### No visible phenotypic change by overexpression of sorghum *SuSy* genes in Arabidopsis

**Figure 6:**
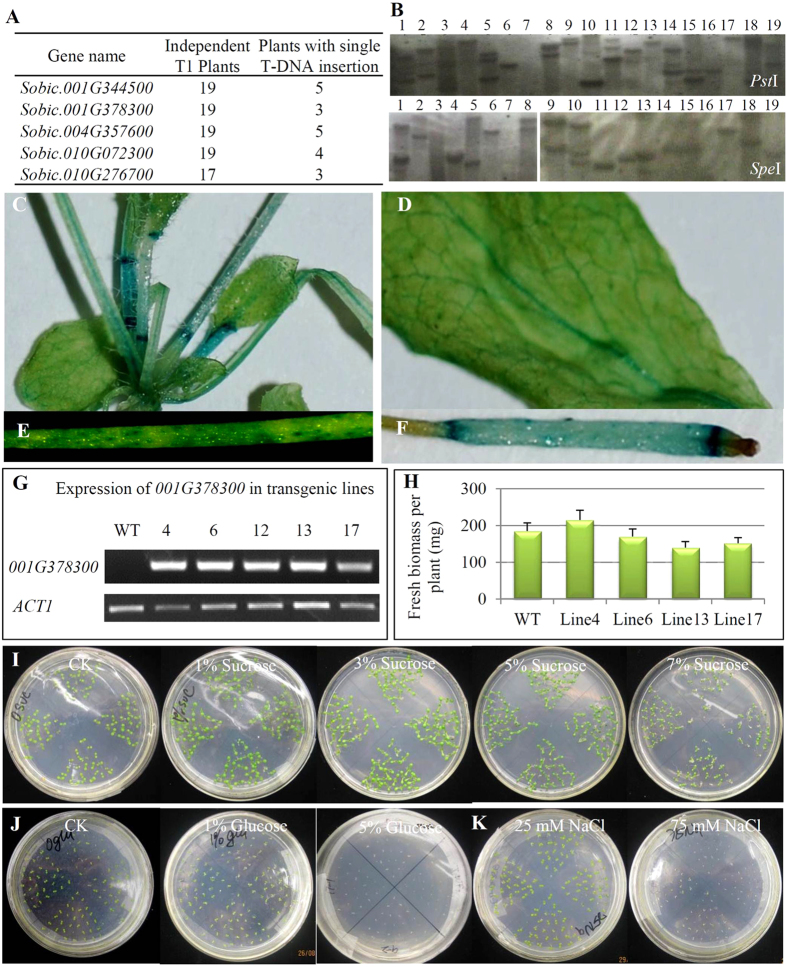
Overexpression of sweet sorghum *SuSy* genes in Arabidopsis. (**A**) A total of 5 sorghum SuSy genes were independently overexpressed in Arabidopsis under the maize ubiquitin promoter to generate at least 3 independent transgenic lines with single copy of T-DNA insertion in each construct. (**B**) An example of copy number detection by Southern blot hybridization. DNA samples were prepared from transgenic Arabidopsis plants overexpressing the sorghum gene *Sobic.001G378300*. Top and bottom panels show the results from DNA samples digested by *Pst*I and *Spe*I, respectively. **(C–F**) GUS activities in transgenic plants carrying the *Sobic.001G378300* promoter::*GUS* construct. **(C)** the whole Arabidopsis plants; **(D)** leaf; (**E**) root; **(F)** silique. **(G)** Expression analysis of transgenic plants overexpressing *Sobic.001G378300*. *ACT1*, an Arabidopsis gene encoding Actin-1 protein with locus name *At2g37620*. The prefix “*Sobic*.” was omitted in the locus name for convenience. (**H**) Investigation of fresh biomass of four independent transgenic plants with single copy of T-DNA insertion. Thirty-day plants grown on media were used for the survey. **(I–K**) Phenotypic observation of transgenic plants grown under different concentrations of sucrose, glucose and NaCl containing media, respectively.

To understand the biological functions of genes encoding these four gene families, we overexpressed these genes under the maize ubiquitin promoter in Arabidopsis. For the *SuSy* family, all the coding regions of 5 genes were isolated from the sweet sorghum Keller by RT-PCR and subcloned into the binary vector pCambio1300 for *Agrobacterium*-mediated transformation. A total of 17 to 19 independent transgenic T1 plants were propagated from T0 generation (Fig. 6A) based on Southern blot hybridization (Fig. 6B). At least 3 independent T2 plants with single T-DNA insertion were selected for further investigation (Fig. 6A,B). Our data showed that no visible phenotypic difference was observed between wild type (WT) Arabidopsis and overexpression transgenic plants. Here we further characterized the sorghum gene *Sobic.001G378300* by investigating its promoter activities and the effects of overexpressing this gene in Arabidopsis. We first analyzed its promoter activities in the promoter::*GUS* transgenic plants. Around 2 Kb of the promoter region upstream of the start codon of this gene was fused with the *GUS* reporter gene and was then delivered into the Arabidopsis genome. The GUS staining results showed that the activities of the promoter could be observed in the whole plants (Fig. 6C) including leaves (Fig. 6D), roots (Fig. 6E) and siliques (Fig. 6F). Further observation showed that the gene showed higher expression level in the phloem tissue of nodes and veins (Fig. 6C,D). We then characterized the transgenic Arabidopsis lines by overexpressing this gene. A total of 5 independent lines were subjected to expression analysis, which showed that the gene was highly expressed in these transgenic plants (Fig. 6G). However, these transgenic lines showed no statistical difference in their biomass yield (Fig. 6H). We also did not observe the difference in brix degree. We then subjected these lines into stress treatments. Under 1-7% sucrose stress, both transgenic plants and WT showed no obvious difference (Fig. 6I). Similarly, no difference was observed under both glucose and NaCl treatments (Fig. 6J,K). In general, no morphological phenotype was observed under normal or stress conditions after overexpression of the gene *Sobic.001G378300*.

### Overexpression of one of sorghum *SPS* genes retards seed germination in Arabidopsis under normal and sugar stress conditions

**Figure 7:**
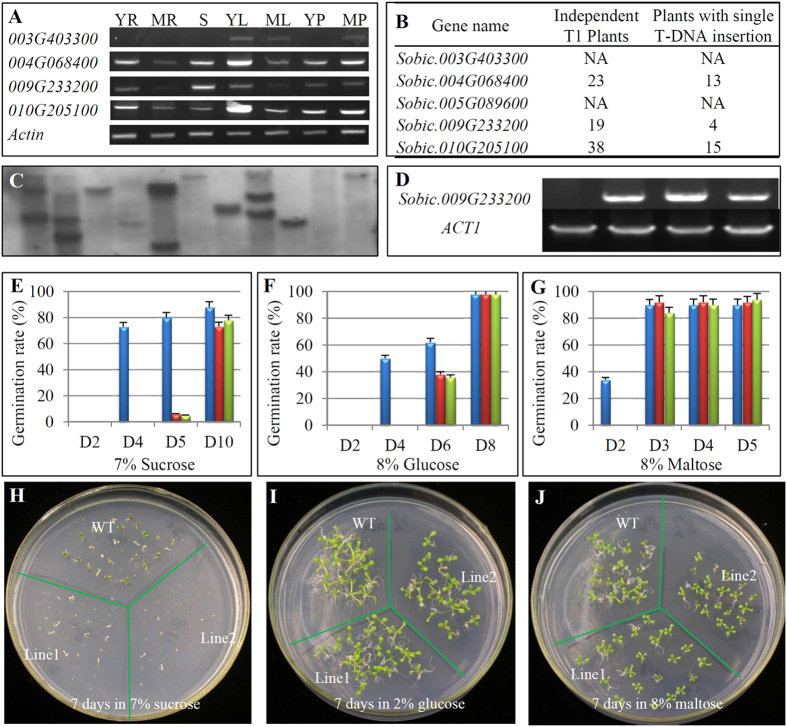
Molecular and phenotypic characterization of transgenic Arabidopsis plants overexpressing sweet sorghum *SPS* genes. (**A**) Expression patterns of 4 *SPS* genes in sweet sorghum Keller by RT-PCR analysis. Total RNA samples were prepared from 7 different tissues. YR, young root, from 14-day seedlings; MR, mature root, from 3-month plants; S, stem, from 30-day plants; YL, young leaf, from 14-day seedlings; ML, mature leaf, from 3-month plants; YP, young panicle, from un-flowering panicles; MP, mature panicle, from flowering panicles. The prefix “*Sobic*.” was omitted in each locus name. (**B**) Three of five sorghum SPS genes were independently overexpressed in Arabidopsis under the maize ubiquitin promoter to generate at least 4 independent transgenic lines with single copy of T-DNA insertion in each construct. NA, not available. **(C)** An example of copy number detection by Southern blot hybridization. DNA samples from 11 transgenic plant (*ubiquitin:: Sobic.009G233200*) were digested by *Pst*I for the hybridization using the probe prepared from the *hygromycin* gene. **(D)** Expression analysis of 3 independent transgenic plants (*ubiquitin::Sobic.009G233200*) with single copy of T-DNA insertion. *ACT1*, an Arabidopsis gene encoding Actin-1 protein with locus name *At2g37620*. **(E**–**G)** Effects of sucrose, glucose and maltose treatments, respectively, on germination rates of WT and transgenic plants. D2, D3, D4, D5, D6, D8 and D10 indicated 2, 3, 4, 5, 6, 8 and 10 days after inoculation on ½ MS media. **(H**–**J)** Phenotypic observation of transgenic plants treated by sucrose, glucose and maltose, respectively, by comparing with WT.

In sorghum, we have identified a total of 5 *SPS* genes. In most of cases, these genes were expressed in multiple tissues (Fig. 7A). However, in each gene, the expression abundance varied among different tissues. Three genes including *Sobic*.*003G403300*, *Sobic*.*004G068400* and *Sobic*.*010G205100* showed the highest transcript level at young leaves and the gene *Sobic.009G233200* exhibited the highest expression abundance in stem (Fig. 7A). To better understand their biological functions, coding regions from three genes were amplified from sweet sorghum by RT-PCR for overexpression under the maize ubiquitin promoter in Arabidopsis (Fig. 7B). By molecular characterization, we identified 13, 4, and 15 independent T2 lines from the genes *Sobic.004G068400*, *Sobic.009G233200* and *Sobic.010G205100*, respectively, which showed high expression level with single copy of T-DNA insertion (Fig. 7B). These lines were then subjected into phenotypic characterization. For both genes *Sobic.004G068400* and *Sobic.010G205100*, no obvious phenotypic variation was observed after overexpression in Arabidopsis when compared with WT plants. Here we focused on the gene *Sobic.009G233200*. Based on Southern blot hybridization, we have selected 4 independent lines with Single copy of T-DNA insertion (Fig. 7C). These lines showed high expression level for the overexpressed gene in Arabidopsis (Fig. 7D). We then subjected these lines into morphological characterization. Our data showed that overexpression of *Sobic.009G233200* significantly retarded seed germination (Fig. 7E–G and Supplementary Fig. S3 A-C). Under normal growth conditions, up to 93% of Arabidopsis WT seeds germinated after 2-day inoculation on ½ MS media whereas significantly lower percentages of seeds germinated for all identified 4 independent transgenic lines. Here we showed the results from two lines indicated by red and green cylinders, respectively in the Fig. 7E–G; Supplementary Figure S3 A-C. Their germination rates in these two lines were only 66% and 35%, respectively, under normal growth media (Supplementary Fig. S3A). Under 1–9% of sucrose stress, germination for both WT and transgenic seeds were significantly retarded. However, transgenic seeds showed more sensitive response to the stress. For example, after 7-day inoculation on 7% sucrose containing MS media, up to 80% of WT seeds germinated whereas only less than 10% seeds germinated for the transgenic lines (Fig. 7E,H). Similarly, germination retardation was also observed for the transgenic seeds when they were germinated under both glucose and maltose stresses (Fig. 7 F and G; Supplementary Fig. S3 B and C). Although the transgenic Arabidopsis plants showed retarded germination under either normal or sugar stress conditions during early days, these lines exhibited no significant difference after more days of incubation on ½ MS media (Fig. 7I,J). From two weeks after germination, we further measured plant height, root length, flowering time and biomass yield during the whole developmental stages and found that no statistical difference was detected between WT and transgenic lines.

As seed germination is regulated by both gibberellic acid (GA) and abscisic acid (ABA), we further surveyed the expression regulation of *Sobic.009G233200* under these two hormone treatments. Our data showed that the gene was upregualted by both GA3 and ABA treatments (Supplementary Fig. S4 A and B). We then examined the effects of GA3 and ABA on seed germination. The analysis showed that germination retardation was neither rescued by GA3 nor further postponed by ABA and no difference was observed between WT and transgenic plants (*ubiquitin::Sobic.009G233200*) under various concentrations of GA3 and ABA treatments after 9 days of germination (Supplementary Fig. S4 C and D).

### Overexpression of one of sorghum *SPP* genes retards seed germination in Arabidopsis only under high salinity stress conditions

**Figure 8:**
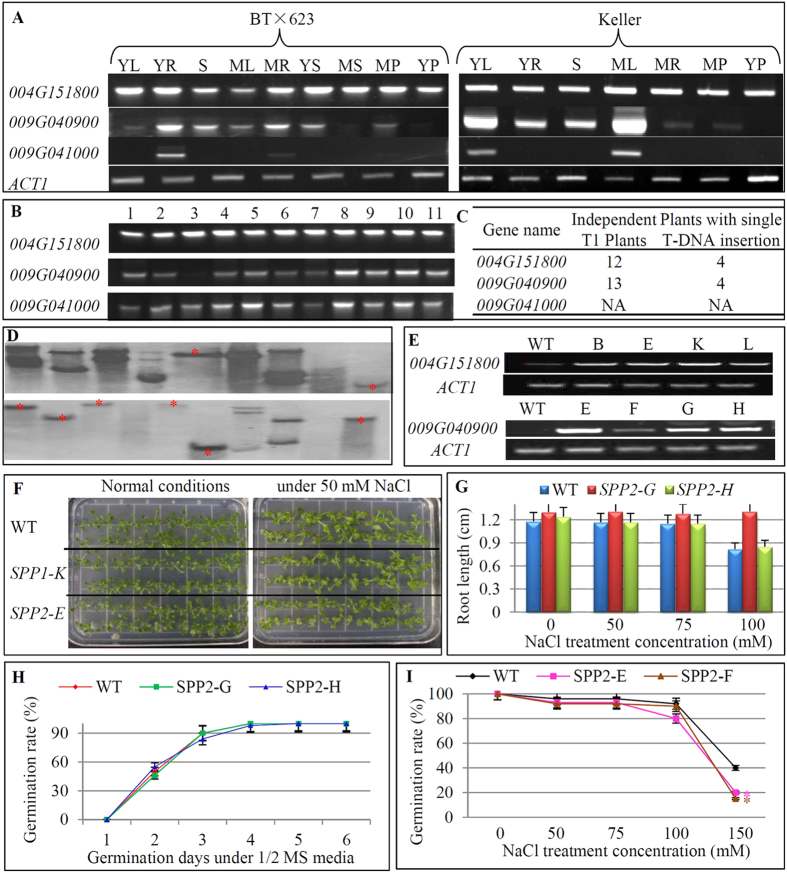
Molecular and phenotypic characterization of transgenic Arabidopsis plants overexpressing sweet sorghum *SPP* genes. (**A**) Comparative expression analysis of 3 sorghum SPP genes between grain (BT × 623) and sweet (Keller) sorghum varieties. YL, YR, S, ML, MR, MP and YP were defined as in Figure 7. YS, young seeds; MS, mature seeds. *ACT1*, an Arabidopsis gene encoding Actin-1 protein with locus name *At2g37620*. (**B**) Expression patterns of 3 SPP sorghum genes under various abiotic stresses and sugar treatments. 1, Control (under normal conditions); 2, 30% PEG 0.5 h; 3, 30% PEG 2 h; 4, 250 mM NaCl 2 h; 5, 250 mM NaCl 8 h; 6, Ice-water at 4 °C 2 h; 7, Ice-water at 4 °C8 h; 8, 5% glucose 2 h; 9, 5% glucose 6 h; 10, 5% sucrose 2 h; 11, 5% sucrose 6 h. **(C)** Two of three sorghum SPP genes were independently overexpressed in Arabidopsis under the maize ubiquitin promoter to generate at least 4 independent transgenic lines with single copy of T-DNA insertion in each construct. NA, not available. **(D)** An example of copy number detection by Southern blot hybridization. DNA samples from 19 transgenic plants (*ubiquitin::Sobic.004G151800*) were digested by *Pst*I for the hybridization using the probe prepared from the *HYGROMYCIN* gene. **(E)** Expression analysis of 4 independent transgenic plants (*ubiquitin::Sobic.*004G151800 and *ubiquitin::Sobic.009G040900*) with single copy of T-DNA insertion. In (**A–E**) the prefix “*Sobic*.” was omitted in each locus name for convenience. **(F)** Phenotypic observation of transgenic plants under normal and high salinity treatment by comparing with WT plants. SPP1 and SPP2, transgenic plants from *ubiquitin::Sobic.004G151800* and *ubiquitin::Sobic.009G040900*, respectively. (**G)** Measurement of root length under high salinity stress for transgenic plants overexpressing *SPP2*. **(H)** Investigation of germination rates between WT and transgenic plants *SPP2* under normal growth conditions. **(I)** Effects of high salinity treatments on germination rate in transgenic plants *SPP2*. The stars “*” in **(I)** indicated significant difference in germination rates between WT and transgenic plants.

In the sorghum genome, a total of 3 *SPP* genes have been identified (Table 1). We carried out the comparative expression analysis between grain (BT × 623) and sweet sorghum (Keller) lines by RT-PCR (Fig. 8A,B). The gene *Sobic.004G151800* showed the similar expression patterns between these two cultivars among tested tissues. This gene was constitutively expressed in all tested tissues with similar transcript abundance. In contrast, the gene *Sobic.009G040900* was not expressed in a few of tested tissues in both BT × 623 and Keller. In BT × 623, the strong expression was detected in young and mature roots as well as stems; no signal was detected in mature seeds and young panicles; the remaining 5 tissues showed relatively less expression level. In Keller, the strongest expression was detected in both young and mature leaves followed by young roots and stems. No expression in young panicles and very faint signal in mature roots and mature panicles were detected. More interestingly, the gene *Sobic.009G041000* was tissue-specific and exhibited the expression divergence between grain and sweet sorghum. In BT × 623, this gene was expressed only in young and mature roots; however, it was leaf-specific in keller (Fig. 8A,B). In addition, we further explored whether these three genes were regulated by some stresses in their expression. The gene *Sobic.004G151800* showed no difference in its expression level under various stresses including drought (polyethylene glycol, PEG), high salinity (NaCl), cold (Ice-water), glucose and sucrose (Fig. 8C). The gene *Sobic.009G040900* was down-regulated by 30% PEG treatment but was up-regulated by both glucose and sucrose. Its expression was not significantly regulated by both high salinity and cold stresses. Interestingly, the gene *Sobic.009G041000* was up-regulated by all the tested 5 stresses.

Currently, no data has been reported on functional characterization of these *SPP* genes in sorghum. We have isolated two of these *SPP* genes from sweet sorghum and overexpressed them separately in the Arabidopsis genome. Among these 12 and 13 transgenic T1 lines overexpressing *Sobic.004G151800* and *Sobic.009G040900*, respectively, we identified 4 independent lines for each gene that were with single T-DNA insertion (Fig. 8D). Expression analysis showed that the gene *Sobic.004G151800* was highly expressed in all 4 independent lines (Fig. 8E). We also detected weak expression signal in WT as this gene shows very high homology to the Arabidopsis ortholog in their coding regions and primers used for RT-PCR may also bind to the ortholog for amplification. Similarly, another gene *Sobic.009G040900* was also highly expressed in 4 independent transgenic lines after overexpression (Fig. 8E). All these 8 lines contained only single copy of T-DNA insertion as shown in Southern blot hybridization (Fig. 8D). All these independent 8 lines were then subjected into phenotypic characterization. We first grew these transgenic lines on ½ MS media together with WT plants and then characterized their phenotypes by measuring their plant height, root length and biomass yield. However, no statistical difference was detected between WT and transgenic plants (Fig. 8F). Since these two sorghum genes were regulated by various stresses (Fig. 8B), we then subjected these lines into high salinity stress. We also could not observe the difference (Fig. 8G; Supplementary Fig. S5A). For example, root length in both WT and transgenic plants from overexpressing either *Sobic.004G151800* (Supplementary Fig. S5A) or *Sobic.009G040900* (Fig. 8G) showed no significant difference under different concentrations of salinity stress. Similarly, no difference was also observed when these transgenic lines were subjected into sucrose, glucose and maltose treatments (Supplementary Fig. S6 A-C). In addition, both WT and transgenic lines also showed no difference in their germination rates (Fig. 8H; Supplementary Fig. S5B). Under the NaCl stress less than 100 mM, no effect was observed on seed germination when compared with WT (Fig. 8I; Supplementary Fig. S5C). However, under 150 mM NaCl-containing media, one transgenic line overexpressing *Sobic.004G151800* showed statistically lower germination rate but no difference was observed for the remaining transgenic lines (Supplementary Fig. S5C). Interestingly, under 150 mM NaCl-containing media, all transgenic lines over-expressing *Sobic.009G040900* showed significantly lower germination rates (Fig. 8I). The data suggested that the gene *Sobic.009G040900* might play a role in the seed germination under high concentration of salinity stress.

Similar to the *SPS* gene *Sobic.009G233200*, we surveyed the expression regulation of *Sobic.009G040900* under GA3 and ABA treatments. The expression analysis showed that the gene was also upregulated by both GA3 and ABA treatments (Supplementary Fig. S7 A and B). Furthermore, we examined the effects of GA3 and ABA on seed germination in WT and transgenic plants *ubiquitin::Sobic.009G040900*. The investigation showed that no difference was observed under various concentrations of GA3 and ABA treatments (Supplementary Fig. S7 C and D).

### Transgenic plants by overexpressing one of sorghum *UDPGP* genes in Arabidopsis increased biomass yield and flowered earlier

**Figure 9:**
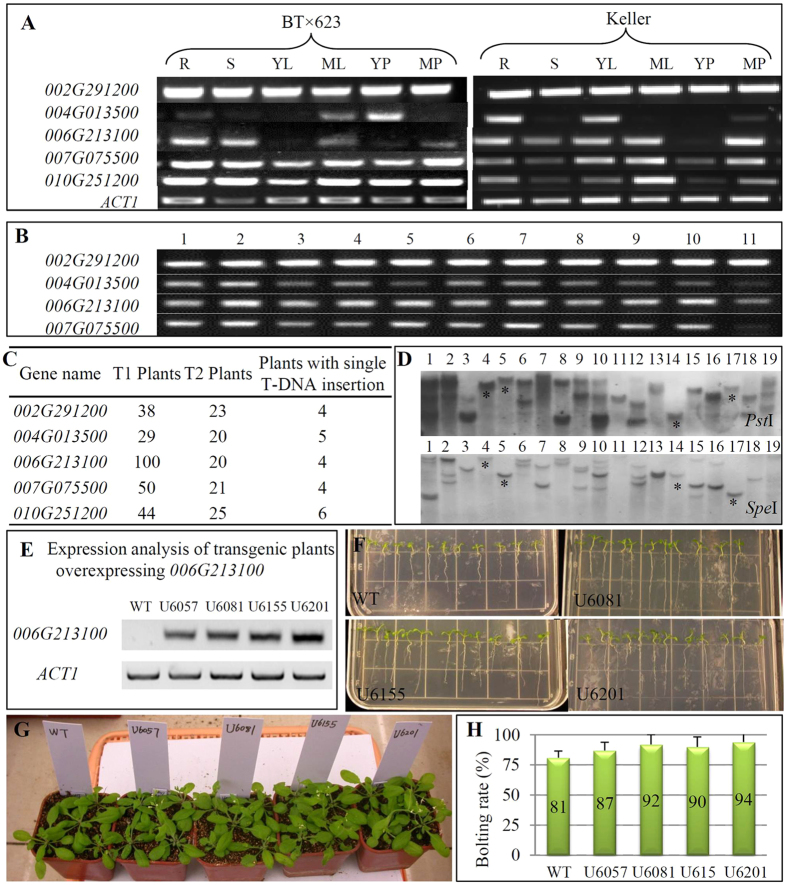
Molecular and functional characterization of the sorghum genes encoding UDPGPs. (**A**) Comparative expression analysis of sorghum *UDPGP* genes between grain (BT × 623) and sweet (Keller) sorghum lines by RT-PCR. R, root; S, stem; YL, young leaf; ML, mature leaf; YP, young panicle; MP, mature panicle. (**B**) Expression regulation of sorghum *UDPGP* genes under various abiotic stresses and sugar treatments. 1, control; 2, 30% PEG-0.5 h; 3, 30% PEG-2 h; 4, 250 mM Nacl-2 h; 5, 250 mM Nacl-4 h; 6, 4 °C Cold-2 h; 7, 4 °C Cold-8 h; 8, 5% Glucose-2 h; 9, 5% Glucose-6 h; 10, 5% Sucrose-2 h; 11, 5% Sucrose-6 h. **(C)** A summary information of transgenic Arabidopsis plants by overexpressing sorghum *UDPGP* genes under the control of the maize promoter ubiquitin. **(D)** Copy number detection of T-DNA insertion in 19 T2 plants by Southern blot hybridization. These lines were generated by overexpressing the sorghum gene *Sobic.006G213100* in the Arabidopsis genome. The DNA samples were digested by either *Pst*I or *Spe*I and were then transferred into nylon membrane for hybridization using the *HYGROMYCIN* probe. The 4 independent T2 lines with single copy of T-DNA insertion in both enzymes were labelled by the star “*”. **(E)** Expression analysis of the *ubiquitin::Sobic.006G213100* transgenic plants. In **(A–E**) the prefix “*Sobic*.” was omitted in each locus name for convenience. **(F)** Phenotypic observation of three independent transgenic lines under normal growth conditions. The data were collected after 8-day growth on ½ MS media. **(G)** Observation of flowering time in 4 independent lines. **(H)** Bolting rates of 4 independent lines.

The sorghum genome encodes 5 members of the *UDPGP* family (Fig. 2A). We investigated their expression patterns by RT-PCR using total RNA samples from both BT × 623 and Keller (Fig. 9A). Except for the gene *Sobic.002G291200*, which was expressed in all tested six tissues including roots, stems, young and mature leaves, young and mature panicles, all the remaining 4 genes showed differential expression between BT × 623 and Keller in either tissues or transcript abundance. For example, the gene *Sobic.004G013500* was mainly expressed in both mature leaves and young panicles in BT × 623 whereas its expression in Keller was mainly detected in both roots and young leaves. Another example is for the gene *Sobic.010G251200*, which was constitutively expressed in all 6 tissues with similar abundance in BT × 623. On contrast, in Keller, the gene was differentially expressed with obviously variable abundance in these 6 tissues. On the other hand, we also carried out the expression analysis of these genes under various stresses. We surveyed the expression regulation under drought (30% PEG), high salinity (250 mM NaCl), cold (ice-water), glucose (5%) and sucrose (5%). Under drought, high salinity, cold and glucose treatments, all analyzed four genes showed no significant difference in their expression level (Fig. 9B). However, under sucrose treatment, both genes *Sobic.004G013500* and *Sobic.007G075500* were down-regulated in their transcript abundance (Fig. 9B).

To further understand the biological functions of these 5 sorghum *UDPGP* genes, we overexpressed them in Arabidopsis. We have generated more than 20 independent T2 plants for each gene, from which we have selected at least 4 independent lines with single copy of T-DNA insertion and with high level of expression abundance (Fig. 9C). All of these lines were subjected into phenotypic characterization. However, our preliminary data showed that no obvious difference was observed in these transgenic lines when compared with WT plants in plant height, root length, flowering time and biomass yield for four genes including *Sobic.002G291200*, *Sobic.004G013500*, *Sobic.007G075500* and *Sobic.010G251200*. We therefore focused on the remaining gene *Sobic.006G213100*. A total of 19 T2 plants were subjected into Southern blot hybridization for T-DNA copy number detection, from which we have identified 4 independent lines with single copy of T-DNA insertion (Fig. 9D). The gene *Sobic.006G213100* showed high transcript abundance after overexpression in these four transgenic lines (Fig. 9E). These lines were grown on ½ MS media for phenotypic measurement, which showed that transgenic roots grew faster (Fig. 9F). The root length from all the 4 independent lines was statistical longer than that in WT plants (Supplementary Fig. S8A). The root length was 2.61 cm on average in WT while the average root length in transgenic lines ranged from 3.49 to 3.70 cm. Similarly, the average fresh weight in 15-day-old transgenic plants ranged from 5.64 to 7.04 mg/plant, significantly heavier than that in WT plant (3.32 mg/plant) (Supplementary Fig. S8B). However, at the mature stage (65-day-old), no significant difference was observed in their fresh weight between WT and transgenic plants (Supplementary Fig. S8C). Interestingly, at the flowering stage, transgenic plants flowered earlier than WT plants (Fig. 9G). When the bolting rate of WT plants reached 81%, the rate in transgenic plants ranged from 87% to 94% (Fig. 9H). The data implied that the gene might play a role in flower timing.

## Discussion

### Evolutionary origins of *SuSy*, *SPS*, *SPP* and *UDPGP* gene families

In this study, we have genome-widely identified four gene families in 15 sequenced genomes. Besides these, we have also genome-widely identified these families in additional 35 species including 2 moss, 6 algae and 27 dicot/monocot plant species, whose whole genome sequences are available (Supplementary Tables S1-S4). In addition, we also detected the distribution of these four gene families in nature by searching both Pfam and Interpro databases. Based on our searching results, the *SuSy*, *SPS* and *SPP* gene families generally presents in higher plants, mosses and bacteria, in which the organelle for photosynthesis has been evolved. All the three family members have been found in bacteria. However, their genomes usually encode only one member in each family and not all bacteria required these family proteins. All tested higher plants including mosses were found to encode at least one member of *SuSy*, *SPS* or *SPP* families. Thus, *SuSy*, *SPS* and *SPP* members are ubiquitous in higher plants. The *SPP* family also ubiquitously exists in algae. However, no *SPS* member was detected in all tested algae species. For the *SuSy* family, we genome-widely identified the family members in 6 sequenced algae genomes including *Chlamydomonas reinhardtii*, *Coccomyxa subellipsoidea C-169*, *Micromonas pusilla* CCMP1545, *Micromonas* sp. RCC299, *Ostreococcus lucimarinus* and *Volvox carteri*. However, only two species *Chlamydomonas reinhardtii* and *Coccomyxa subellipsoidea* C-169 encoded *SuSy* genes and no *SuSy* gene was detected in the remaining 4 genomes. On the other hand, the *SPP* family was also found in a few of fungus species and both *SuSy* and *SPP* members can be detected in Archaea. By searching the Pfam and Interpro databases, we obtained one sequence from marine *thaumarchaeote* (archaea) with accession number A0A075I6C7, which encodes an ornithine carbamoyltransferase. The sequence showed high homology to the *SuSy* domain. We have also detected two sequences from *archaea* encoding the SPP domain. The presence of the *SPP* family was also detected in some species of fungi. However, no sequence was detected in either archaea or fungi to encode a SPS protein (Supplementary Table S5). SPS proteins contain both domains with Pfam ID PF00534 and PF05116 (Methods). In green algae, both proteins with Pfam ID either PF00534 or PF05116 were detectable. However, no protein was identified to have these two domains. Thus, no SPS existence in green algae might be due to that no domain combination occurred in these two domains. On the contrary, the *UDPGP* family exists not only in monocot/dicot plants, mosses, algae and bacteria but also in animals and fungi although we could not detect any member in both archaea and virus (Table S5).

Previous data showed that archaea genomes did not encode any members of these sucrose synthesis related families^17^. However, we have detected homologous sequences in archaea to encode both SuSy and SPP domains (Supplementary Table S5). On the other hand, *SuSy* and *SPP* were widely detected in photosynthetic bacteria. Thus, based on our data, the *SuSy* and *SPP* families might be originated from archaea and then singular cell organism with the organelle for photosynthesis. Comparing to *SuSy*, *SPS* and *SPP* families, the *UDPGP* family exhibited distinct difference in its evolutionary history. It presents not only in plants but also in animals (Supplementary Table S5). Thus, our data showed that these four gene families underwent different origin and evolutionary histories.

For all four gene families, a genome from archaea, bacterium or fungus usually encodes only one member in each family and higher plant genomes encode various sizes of family members. At the early stage of evolutionary history, very low expansion occurred and a genome generally encodes a few members. This situation existed until the divergence between monocots and dicots (Fig. 1). A large scale of expansion occurred only for some species during the divergence from one species to another. As a result, different species encode different sizes of family members ranging from 4 to more than 30 genes (Fig. 1 and Table 1). During the evolution, domain combinations were frequently observed among the domains from SuSy, SPS and SPP. Domain combinations were the processes that generated new genes and functional divergences^43^ (Apic *et al.* 2001). One of the examples is the evolution of SPP enzymes, which consist of at least two domains. One is named as S6PP (PF05116) and another is Glycos_transf_1 (PF00534). The former comes from SPP enzymes and the latter comes from glycosyl transferases. Their combination led to the birth of *SPS* genes. Although both *SuSy* and *SPP* genes were detected in both algae and archaea, no *SPS* gene was evolved as no domain combination occurred between S6PP and Glycos_transf_1 domains in algae or archaea.

### Expansion history and mechanisms of *SuSy*, *SPS*, *SPP* and *UDPGP* gene families

Although four gene families exhibited the difference in their expansion patterns, they also showed some similarities. Before the divergence between monocot and dicot plants, four gene families experienced very low expansion and a MRCA genome encoded only one (for *SPP*), two (for *SuSy* and *SPS*) or three (for *UDPGP*) genes. After the divergence between dicots and monocots, monocot plant expanded faster than dicot plants as more members were detected in the MRCA among 4 monocot species than that among dicot species (Fig. 1). However, a large scale of expansion occurred during species divergence for both dicot and monocot plants as their MRCA family sizes were very small when compared with current family sizes (Fig. 1 and Table 1). The data suggested the recently expansion and evolutionary events for these gene families. Furthermore, the expansion was also detected during the divergence of sub-species. One of the examples is the expansion of additional *UDPGP* member in indica rice 93–11. Generally, the japonica rice Nipponbare genome encodes a total of 6 *UDPGP* genes while a total of 7 genes are encoded in the indica genome. The additional member *BGIOSGA003207* was recently tandem duplicated from *BGIOSGA003204* after the divergence between indica and japonica. Different sub-species might experience different expansion patterns. As a result, some members might be expanded in a sub-species but not in another sub-species, which might contribute to intra-species divergence and the formation of sub-species. For example, the japonica rice genome encodes 7 *SuSy* genes including *LOC_Os02g58480*, *LOC_Os03g22120*, *LOC_Os03g28330*, *LOC_Os04g17650*, *LOC_Os04g24430*, *LOC_Os06g09450* and *LOC_Os07g42490*. However, the indica rice genome encodes 8 members. Phylogenetic analysis showed that the MRCA between two sub-species contained 6 *SuSy* genes (Supplementary Fig. S9A). During the divergence of these two sub-species, the member located on chromosome 2 tandemly duplicated and gave birth to two members while only one member was detected in the japonica genome (Supplementary Fig. S9 A and B). A similar situation was also observed on chromosome 6 (Supplementary Fig. S9 A and C). However, on chromosome 4, one more gene was expanded, which occurred in the japonica genome (Supplementary Fig. S9 A and D). As a result, the indica rice genome encodes 6 *SuSy* genes instead of 7 members in japonica.

Many mechanisms have been involved in gene expansion. Among them, genome-wide duplication significantly contributed to gene expansion^44^. In this study, we have detected the contribution of genome-wide duplication to the expansion of both *SuSy* and *UDPGP* gene families in soybean, apple and maize (Fig. 2A,B). Besides the whole genome duplication, both tandem and segmental duplications also significantly contributed to gene expansion^45^. Our data showed that segmental duplication contributed to the expansion of four gene families in most of species. However, tandem duplication contributed to the gene expansion only in a few species. In addition to these, we also explored the contribution of transposons/retrotransposons to the expansion of these four gene families. We found that transposons/retrotransposons played roles in the expansion of a few genes in some gene families. They might not contribute to the large scale of expansion of these four gene families. Generally, multiple molecular mechanisms have been involved in the expansion of these four gene families. Comprehensive expansion patterns have been observed, which varied among different gene families and different species. The data might imply that species-specific functions have been evolved in some gene families.

Expanded genes from various expansion mechanisms also frequently lost during long evolution due to redundant functions^45^. The fact might explain why both *SPS* and *SPP* gene families were not obviously expanded. After gene expansion, extra copies of genes might evolve into pseudogenes or genes with subfunctions/new functions. The *Ka/Ks* analysis showed that different gene families exhibited different rates of *Ka/Ks* ratios, suggesting the different selection force among different families/species (Fig. 3). The newly born genes might also keep the similar functions but survive with differential expression patterns compared with their parental genes. Our data showed that no same expression pattern was observed among members from the same family (Fig. 4). These data suggested the contribution of expression divergence to the survival of genes after expansion.

### Recent positive selection occurred only during intra-species divergence in the sorghum *UDPGP* family

Our data showed that the divergence of genes from 4 gene families among distantly related species has been driven by purifying selection (Fig. 3). We have also surveyed the divergence of these genes within the species *Sorgum bicolor*, which indicated that most of these genes were under purifying selection with *Ka/Ks* less than 1 (Fig. 5). However, we detected one of *UDPGP* genes *Sobic.006G213100*, which was under positive selection with *Ka/Ks* = 1.574 (Fig. 5A). To further investigate the presence of the positive selection among a larger population within the species, we analyzed a total of 44 sorghum lines, in which the whole genome re-sequencing data are available^46^. We used BT × 623 as the reference genome and found 19 alleles with *Ka/Ks* > 1 whereas the remaining 25 alleles with *Ka/Ks* far less than 1. We have detected a total of 7 positively selected sites in the whole coding region (Fig. 5C). Among them, 3 sites were within the UDPGP domain region. The data suggested that the gene *Sobic.006G213100* should have been suffered from positive selection during the divergence within the species, which, in turn, implied the role of this allele in divergence of sub-functions of this gene. Gene variants referring to adaptive phenotypes are positively selected^47^. Positively selected genes were frequently reported to play roles in signal transduction, cell-cell recognition, immune response, sexual reproduction, membrane and intracellular transporters^48^. For the *UDPGP* family genes, positive selection was also detected in the species *Barbarea vulgaris*^49^. In the species, two genes *UGT73C10* and *UGT73C11* were positively selected and they played roles in saponin-mediated insect resistance. Besides sorghum and *Barbarea*, we also investigated the presence or absence of positive selection of the *UDPGP* family genes in the *Oryza sativa* species as re-sequencing data from hundreds of thousands of lines from this species are available. We have analyzed a total of 1485 rice lines, whose re-sequencing data are available. However, no positive selection was detected in the whole coding region of a total of 6 *UDPGP* rice genes. Thus, positive selection in the *UDPGP* family occurred only during adaptive divergence within certain species. For these species, for example, both sorghum and *Barbarea*, positive selection should play important roles in adaptive divergence and evolution within species.

### Newly revealed biological functions of *SPS*, *SPP* and *UDPGP* gene families

Sweet sorghum has been recognized widely as potential alternative source of bio-fuel due to its high fermentable sugar content in the stalk. However, little is known on sucrose metabolism related genes and their families in sorghum. In this study, we first genome-widely identified all genes encoding SuSy, SPS, SPP and UDPGP proteins and investigated their biological functions by overexpressing these genes in the Arabidopsis genome. For the sorghum *SuSy* gene family, we have generated transgenic Arabidopsis plants by overexpressing 5 *SuSy* genes (Fig. 6). However, no visible phenotypic variation was observed in these transgenic lines under normal or sugar stress conditions (Fig. 6). Bieniawska *et al.* (2007) analyzed biological functions of 6 Arabidopsis *SuSy* genes using corresponding T-DNA insertion mutants^25^. Their data showed that no obvious difference was observed between WT and mutants in growth phenotypes as well as in starch, sugar and cellulose content, seed weight or seed composition. Further investigation showed that sucrose synthase activity in the *sus1/sus2/sus3/sus4* Arabidopsis mutant is sufficient to support normal cellulose and starch production^50^. Thus, knockout of one *SuSy* gene by T-DNA insertion could be complemented by other *SuSy* genes and as a result, exhibited no obvious phenotype. In addition, multiple studies were reported on overexpression of exogenous *SuSy* genes in the Arabidopsis or other genomes^18^,^19^,^20^. For example, overexpression of cotton *SuSy* gene in poplar increased cellulose content and crystallinity^19^. However, overexpression of the same gene in tobacco did not observe the similar phenotypes^18^. Thus, our and others’ data suggested that plants might grow normally without visible change when one of their *SuSy* gene was knocked out or a *SuSy* gene was overexpressed.

Both SPS and SPP are key enzymes for sucrose synthesis. Overexpression of *SPS* or *SPP* significantly improved sucrose content and as a result, led to various phenotypes including biomass accumulation, promoting growth^27^, carbon metabolism^28^ and improved fiber quality^26^. Reduced expression of *SPS*/*SPP* genes by RNAi or knockout by T-DNA insertion resulted in abnormal development^13^,^14^,^29^. In this study, we have overexpressed 3 *SPS* and 2 *SPP* sorghum genes in the Arabidopsis genome. We found that overexpression of a sorghum *SPS* gene *Sobic.009G233200* in Arabidopsis retarded seed germination under normal and sugar stress conditions (Fig. 7). However, overexpression of a sorghum *SPP* gene *Sobic*.*009G040900* in Arabidopsis postponed seed germination only under high salinity stress (Fig. 8). No effect was observed for the remaining analysed *SPS*/*SPP* genes. To our knowledge, no data has been reported on the effect of *SPS*/*SPP* genes on seed germination. Thus, our data might provide some evidence for the new biological functions of *SPS*/*SPP* genes in seed germination.

Besides *SuSy* and *SPS*/*SPP* genes, we have also surveyed the biological functions of genes encoding UDPGP. We have generated transgenic Arabidopsis plants from 5 constructs by overexpressing 5 sorghum *UDPGP* genes (Fig. 9). Transgenic plants from 4 constructs showed no obvious difference when compared with WT plants. Only the transgenic plants by overexpressing *Sobic.006G213100* showed higher biomass yield and flowered earlier than WT plants (Fig. 9). The increased biomass accumulation during vegetative growth stage was also observed in transgenic Arabidopsis plants by overexpressing an exogenous *UDPGP* gene from *Larix gmelinii*^37^. The earlier flowering by overexpressing an *UDPGP* gene was not yet reported. However, delayed flowering by silencing an *UDPGP* gene through RNAi has been observed^33^. In addition to the function in biomass accumulation and flowering, other functions of *UDPGP* genes have been reported including the roles in pollen callose deposition and male fertility^33^,^34^, which were not observed in this study.

In general, we have generated transgenic Arabidopsis plants by overexpressing 15 sorghum genes encoding SuSy, SPS, SPP or UDPGP enzymes. Most of the transgenic plants showed no obvious phenotype when compared with WT plants under either normal or sugar/salinity stress conditions. These might be partially due to that most of the enzymes catalyze reversible reactions, which might result in limited increase in sucrose synthesis. However, we still observed some phenotypes by overexpressing sorghum *SPS*, *SPP* and *UDPGP* genes in Arabidopsis. Both *SPS* and *SPP* genes showed the biological functions in seed germination, which were not reported previously and suggested the newly discovered function of sorghum *SPS*/*SPP* genes. The sorghum *UDPGP* gene *Sobic.006G213100* was under positive selection and its overexpression in Arabidopsis exhibited its functions in biomass accumulation and shortening flowering time. The gene might play a role in adaptive evolution for changed ecological conditions during intra-species divergence. Thus, sucrose metabolism related genes play roles in not only sucrose synthesis and utilization but also being involved in other biological processes as well as adaptive evolution.

## Methods

### Plant materials and growth conditions

Both grain (BT × 623) and sweet (Keller) sorghum cultivars were collected from National Plant Germplasm System, United States Department of Agriculture (http://www.ars-grin.gov/npgs/). They were planted in greenhouse and were grown under natural light and temperature conditions.

### Abiotic stress and sugar treatments

The 14-day-old seedlings from both BT × 623 and Keller were collected from pots. After careful washing out soil from roots, the whole plants were subjected into sugar or various abiotic stress treatments. For sugar treatments, the whole seedlings were subjected to 5% glucose and sucrose solutions, respectively. Samples were then collected at 0, 2, and 8 h intervals for glucose treatment and 0, 2, and 6 h intervals for sucrose treatment for total RNA preparation. For drought, high salinity and cold stresses, seedlings were treated with 30% PEG, 250 mM NaCl solution or ice-containing water, respectively. Samples were collected at different time intervals (0, 0.5 and 2.0 h for drought and 0, 2 and 9 h for salinity and cold stresses) for total RNA isolation.

### Total RNA preparation and gene expression analysis

To survey the expression patterns among various developmental stages, tissues from different stages were collected including young leaf and root from 14-day-old seedlings, mature leaf, root and stem from 3-month plants, young and mature panicle from un-flowering and flowering plants, respectively. Samples from these tissues and various abiotic and sugar treatments were used for total RNA isolation using a QIAGEN (Hilden, Germany) RNeasy Mini Kit. The first-strand cDNAs were then synthesized from the total RNA samples using an Invitrogen kit. The synthesized cDNA samples were used for detecting expression patterns among different tissues or among various treatments. All polymerase chain reaction (PCR) reactions were carried out in 20 μL reaction mixtures (20 ng cDNA, 200 μM dNTPs, 2.5 mM MaCl_2_, 0.5 μM primers and 1 unit QIAGEN Taq DNA polymerase and 1 × PCR buffer). The temperature profile for PCR is as follows: 94 °C for 2 min followed by 30 cycles at 94 °C for 10 s, 55–65 °C for 30 s (depending on annealing temperature of primer sets) and 7 °C for 1 min. The reaction was stopped by a 5 min extension step at 72 °C. PCR products were visualized by ethidium bromide staining in agarose gels. Photographs of the gels were recorded using Gel Documentation System from Bio-Rad. The qRT-PCR analysis was carried out using total RNA samples from both BT × 623 and Keller seedlings (two-week-old) according to our previous description^41^. Primer sequences used for qRT-PCR were listed in Supplementary Table S6.

### Gene isolation, vector construction for promoter characterization and overexpression and *Agrobacterium*-mediated transformation

Protein coding regions of 15 genes were amplified by RT-PCR with total RNA samples from the whole seedling plants (Keller) as templates. RT-PCRs were performed using QIAGEN One-Step RT-PCR Kit. The designed primer sets were then subjected to the phytozome sorghum database (http://phytozome.jgi.doe.gov/pz/portal.html#!info?alias = Org_Sbicolor) for a BLAST search to eliminate the non-specific primers. The Supplementary Table S6 lists all the primer sequences used for RT-PCR. PCR products were purified from agarose gels by QIAGEN QIAquick Gel Extraction Kit for sequencing. After sequencing verification, the amplified coding region fragments were subcloned into pCAMBIA1300 Ti-derived binary vector (CAMBIA, Canberra, Australia; http://www.cambia.org.au) under the control of the maize ubiquitin promoter. For promoter::*GUS* analysis, around 2 Kb of the sorghum *Sobic.001G378300* promoter was amplified from the sorghum (Keller) genomic DNA using the primer set listed in the Supplementary Table S6. After verification by sequencing, the fragment was cloned in front of *GUS* reporter gene and then subcloned into pCAMBIA1300 Ti-derived binary vector. All the constructs were transformed into the *Arabidopsis thaliana* Columbia genome by the *Agrobacterium*-mediated method^51^.

For histochemical analysis, the GUS staining solution was composed of 0.02 M 5-bromo-4-chloro-3-indolyl-bb-D-glucuronide, 0.1 M NaH_2_PO4, 0.25 M ethylenediaminetetraacetic acid (EDTA), 5 mM potassium ferricyanide, 5 mM potassium ferrocyanide and 1.0% (v/v) Triton X-100 (pH 7.0). Various stages of leaves, flowers and roots were freshly collected from the promoter::*GUS* transgenic Arabidopsis plants (T1 and T2 generations) for the detection of GUS activities. These samples were incubated with the staining solution for overnight at 37 °C. Samples were then subjected to GUS activity observation and photographing after being decolorized by a series concentrations of alcohol.

### Molecular and phenotypic characterization of transgenic plants

The copy numbers of T-DNA insertion in transgenic Arabidopsis plants were analyzed by Southern blot hybridization. A total of 6 μg DNA samples were digested with restriction enzymes *Pst*I or *Spe*I and were then separated by 0.7% agarose gels. The separated DNA samples were transferred onto nylon membranes for probe hybridization. Probes were synthesized by PCRs with the DIG Probe Synthesis Kit (Roche), using the primers listed in Supplementary Table S6. Southern blot hybridizations were carried out according to the manufacturer’s instructions (Boehringer Mannheim). Chemiluminescence was detected using CDP Star substrate (Roche Applied Science).

The transgenic lines with single copy of T-DNA insertion were subjected to expression analysis by RT-PCR using the gene-specific primer sets listed in Supplementary Table S6. Only these lines with single T-DNA insertion and with high expression level for the targeted gene were subjected to phenotypic investigation. Both WT and transgenic lines were germinated and grown under MS media with or without abiotic /sugar stresses. For biomass analysis, both WT and transgenic lines were also grown in soil pots under similar growth conditions. We have surveyed various phenotypes including germination rate, root length, plant height, biomass weight and flowering time according to the description.

### Publicly available expression data and their processes

To investigate the expression divergence of *SuSy*, *SPS*, *SPP* and *UDPGP* genes in apple (*M. domestica*) and maize (*Z. mays*), some of microarray and RNA_Seq datasets were downloaded from the NCBI database (http://www.ncbi.nlm.nih.gov). For the apple Borkh, dataset with accession number GSE42873 were used, which represented transcriptional profiling of 32 different tissues. For maize B73, microarray data with accession number GSE27004 was downloaded, which consisted of 180 samples. As no microarray probe is available for some of maize *SuSy*, *SPS*, *SPP* and *UDPGP* genes, we also downloaded RNA_seq data with accession number SRA012297 for expression analysis of these genes with no microarray probe. After downloaded, both microarray and RNA_Seq data were processed and analyzed by using the CLC Genomics Workbench software (CLC Bio, Swansea, UK). Expression divergence between most recently expanded genes was determined by Student’s *t*-test. These genes with a statistical difference at P < 0.05 were regarded as differentially expressed genes.

### Genome sequences and their annotation as well as re-sequencing data

A total of 50 whole genome sequences and their annotation data were downloaded from the Phytozome database (Phytozome 10.1, http://phytozome.jgi.doe.gov/pz/portal.html). A total of 44 sorghum lines were re-sequenced^46^ and their re-sequencing data were downloaded from the NCBI Short Read Archive database under accession numbers SRS378430 to SRS378473. For rice, the genome sequence data from a total of 1483 rice germplasm lines were available for download from the RiceVarMap database^52^ (http://ricevarmap.ncpgr.cn/) and we used the data for our analysis.

### Genome-wide identification of genes encoding *SuSy*, *SPS*, *SPP* and *UDPGP*

All the SuSy and UDPGP proteins contain a conserved domain with Pfam (http://pfam.sanger.ac.uk/) ID PF00862 and PF01704, respectively. All SPS proteins contain both domains with Pfam ID PF00534 and PF05116. However, SPP proteins have only one of them with Pfam ID PF05116. Thus, we identified these four gene families based on their domain constitute and sequences. We have employed both profile HMM and BLAST searches to genome-widely identify all putative genes encoding SuSy, SPS, SPP and UDPGP enzymes in all the 50 genomes. To build HMM profiles, representative domain protein sequences for PF00862, PF01704, PF05116 and PF00534 were retrieved from the Pfam database. These domain sequences were then aligned using ClustalX 2.0 (www.clustal.org) and were then used to generate HMM profiles for HMM searches with E-value cutoff of 1.0 against annotated protein databases from all the 50 genomes. Besides the HMM searches, BLASTP searches were also performed using the domain sequences as queries to figure out more members in corresponding genomes. These identified members from both HMM and BLASTP searches were then submitted to domain searches to confirm the presence of corresponding domains with E-value = 0.01 as cutoff level.

### Domain amino acid sequence alignment and phylogenetic analysis

The program ClustalX 2.0 was used for the domain amino acid sequence alignment, which was used for the construction of phylogenetic trees according to the description by Jiang *et al.* (2008)^53^. For the SuSy, SPP and UDPGP families, amino acid sequences from only one corresponding domain were used for alignment and tree construction. For the SPS family, both domains PF00534 and PF05116 were used for sequence alignment and phylogenetic analysis.

### Detection of tandemly and segmentally duplicated genes in 15 genomes

Tandemly duplicated genes were determined by sequence identity and physical positions of corresponding genes. They should meet the following criteria: (1) aligned regions should cover at least 30% of query sequences by BLAST search using BLOSUM62 matrix with an E value of less than 0.01; (2) sequence identity should be 70% or greater; (3) the duplicated genes should be less than or equal to 10 genes apart; and (4) tandem pairs should be within 100 Kb for algae and Arabidopsis and 350 Kb for the remaining genomes as suggested by Lehti-Shiu *et al.* (2009)^54^.

Segmentally duplicated genes were determined if these genes were located on segmentally duplicated chromosome blocks. Sequence regions franking 50 kb upstream and downstream of targeted genes were submitted to the identification of segmentally duplicated blocks by using the DAGchainer program^55^. A minimum of 5 gene pairs was used to define a block.

### Detection of transposon/retrotransposon-related expansion of *SuSy*, *SPS*, *SPP* and *UDPGP* genes

To survey the contribution of retrotransposons and DNA transposons to the expansion of the *SuSy*, *SPS*, *SPP* and *UDPGP* gene families, we identified all these mobile elements in the flanking genomic sequence regions of the 50 kb upstream and downstream of targeted genes. We only investigated the contribution of full-length retrotransposons to the gene family expansion as it is difficult to determine the contribution of non-intact retrotransposons. We identified the full-length retrotransposons by the LTR_FINDER program^56^. If a gene was located within a full-length retrotransposon the gene was regarded as retrotransposon related gene. Besides retrotransposons, we also surveyed the contribution of retrogenes to the family expansion. To detect possible retrogenes in these four gene families, genes with only single exon were selected and their amino acid sequences were subjected to BLASTP searches against all of the remaining proteins encoded by genes with two or more exons. Base on the BLASTP results, retrogenes were identified according to the criteria as described by Wang *et al.* (2006)^57^.

To determine the contribution of DNA transposons to the gene family expansion, the same flanking genomic sequences of targeted genes were submitted to the identification of four major transposon family members, which were *mutator-like transposable element (MULE)*, *hAT*, *CACTA* and *Helitron* families. We identified these elements according to the previous description^58^.

### Estimation of *Ka/Ks* ratios and detection of amino acid sites with purifying/positive selections

To estimate *Ka*, *Ks* and their ratios of a pair of genes, which were regarded as an expanded pair, amino acid sequences were aligned and were subsequently transferred to the original cDNA sequences. Pairs were primarily selected according to phylogenetic trees. Only alignments longer than 150 bp with at least 70% identity were selected when incomplete overlap were found in a pair. Both *Ka* and *Ks* values were then estimated from their aligned cDNA sequences using the yn00 program of the PAML4b package^59^.

To detect purifying/positively selected amino acid sites, phylogenetic trees were first constructed. The amino acid alignment, which was used for tree construction, was then transferred to the original cDNA sequences. Both phylogenetic trees and cDNA sequence alignment were used to detect corresponding amino acid sites with purifying/positive selection according to the “sitewise likelihood-ratio" (SLR) method^40^.

To determine positive selection within intra-species, re-sequence data from 44 sorghum lines^46^ and 1483 rice germplasm lines^52^ were used to achieve cDNA sequences and their corresponding protein sequences for all the four gene family members. Based on these sequence data, the positively selected sites were detected by the SLR program.

## Additional Information

**How to cite this article**: Jiang, S.-Y. *et al.* Sucrose metabolism gene families and their biological functions. *Sci. Rep.* **5**, 17583; doi: 10.1038/srep17583 (2015).

## Supplementary Material

Supplementary Information

## Figures and Tables

**Table 1:** Genome-wide identification of *SuSy*, *SPS*, *SPP* and *UDPGP* encoding genes in the 15 completely sequenced plant genomes.

Classification	Species name	Common Name	Genome size (Mb)	Annotated loci	*SuSy*	*SPS*	*SPP*	*UGPGP*
Algae	*C. reinhardtii*	Green algae	111.1	17741	1	0	4	3
Spikemoss	*S. moellendorffii*	Spikemoss	212.5	22273	1	3	5	3
Monocots	*B. distachyon*	Purple false brome	272	31694	6	5	2	5
Monocots	*O. sativa*	Rice	372	55986	7	5	3	6
Monocots	*S. bicolor*	Sorghum	726.6	33032	5	5	3	5
Monocots	*Z. mays*	Maize	2300	32000	20	7	2	17
Dicots	*A. thaliana*	Thale cress	135	27416	6	4	4	6
Dicots	*F. vesca*	Strawberry	240	32831	6	4	5	5
Dicots	*G. max*	Soybean	978	56044	13	7	3	15
Dicots	*M. domestica*	Apple	881.3	63514	31	7	4	14
Dicots	*M. guttatus*	Monkey flower	321.7	26718	4	4	1	4
Dicots	*M. truncatula*	Barrel medic	241	50894	15	3	2	7
Dicots	*P. trichocarpa*	Poplar	422.9	41335	8	6	3	9
Dicots	*R. communis*	Castor bean	400	31221	6	3	1	5
Dicots	*S. lycopersicum*	Tomato	900	34727	7	4	2	6
Total	15	–	–	–	136	67	44	110
