# Supplementary material for: Sucrose metabolism gene families and their biological functions
Source: Sci Rep. 2015 Nov 30;5:17583. doi: 10.1038/srep17583 (PMC4663468; doi:10.1038/srep17583)
Supplement: Supplementary Information [file srep17583-s1.pdf]

Sucrose metabolism gene families and their biological functions

Shu-Ye Jiang [1, 2], Yun-Hua Chi [2], Ji-Zhou Wang [2], Jun-Xia Zhou [2], Yan-Song Cheng [2], Bao-Lan Zhang [2], Ali Ma [2], Jeevanandam Vanitha [2], Srinivasan Ramachandran [1, 2]*

[1] Genome Structural Biology Group, Temasek Life Sciences Laboratory, National University of Singapore, Singapore 117604

[2] TLL-IOB Joint R&D Laboratory, Institute of Botany, Chinese Academy of Sciences, Beijing 100093, China

***Corresponding author:** Srinivasan Ramachandran (sri@tll.org.sg)

Supplementary information

Supplementary Figure S1. Sucrose metabolism pathway.

Supplementary Figure S2. Enlarged phylogenetic analyses of the *SuSy* (A), *SPS* (B), *SPP* (C) and *UDPGP* (D) families.

Supplementary Figure S3. Effects of various sugar stresses on seed germination of transgenic plants overexpressing *Sobic.009G233200*

Supplementary Figure S4. Expression regulation of *Sobic.009G233200* in sorghum under Gibberellic acid (GA) and abscisic acid (ABA) and their effects on seed germination in transgenic Arabidopsis plants.

Supplementary Figure S5. Phenotypic characterization of transgenic plants overexpressing *Sobic.004G151800 (SPP1).*

Supplementary Figure S6. Phenotypic investigation of transgenic plants by overexpressing sorghum gene *Sobic.004G151800* or *Sobic.009G040900* under sugar treatment.

Supplementary Figure S7. Expression regulation of *Sobic.009G040900* in sorghum under Gibberellic acid (GA) and abscisic acid (ABA) and their effects on seed germination in transgenic Arabidopsis plants.

Supplementary Figure S8. Phenotypic characterization of transgenic plants overexpressing *Sobic.006G213100* in Arabidopsis.

Supplementary Figure S9. Differentiated expansion of the *SuSy* gene family between indica and japonica genomes.

Supplementary Table S1. Genome-wide identification of *SuSy* genes in 50 sequenced genomes.

Supplementary Table S2. Genome-wide identification of *SPS* genes in 50 sequenced genomes.

Supplementary Table S3. Genome-wide identification of *SPP* genes in 50 sequenced genomes.

Supplementary Table S4. Genome-wide identification of *UDPGP* genes in 50 sequenced genomes.

Supplementary Table S5. Distribution of *SuSy*, *SPS*, *SPP* and *UDPGP* gene families.

Supplementary Table S6. Primer sequences used for this study.

Supplementary Figure S1.

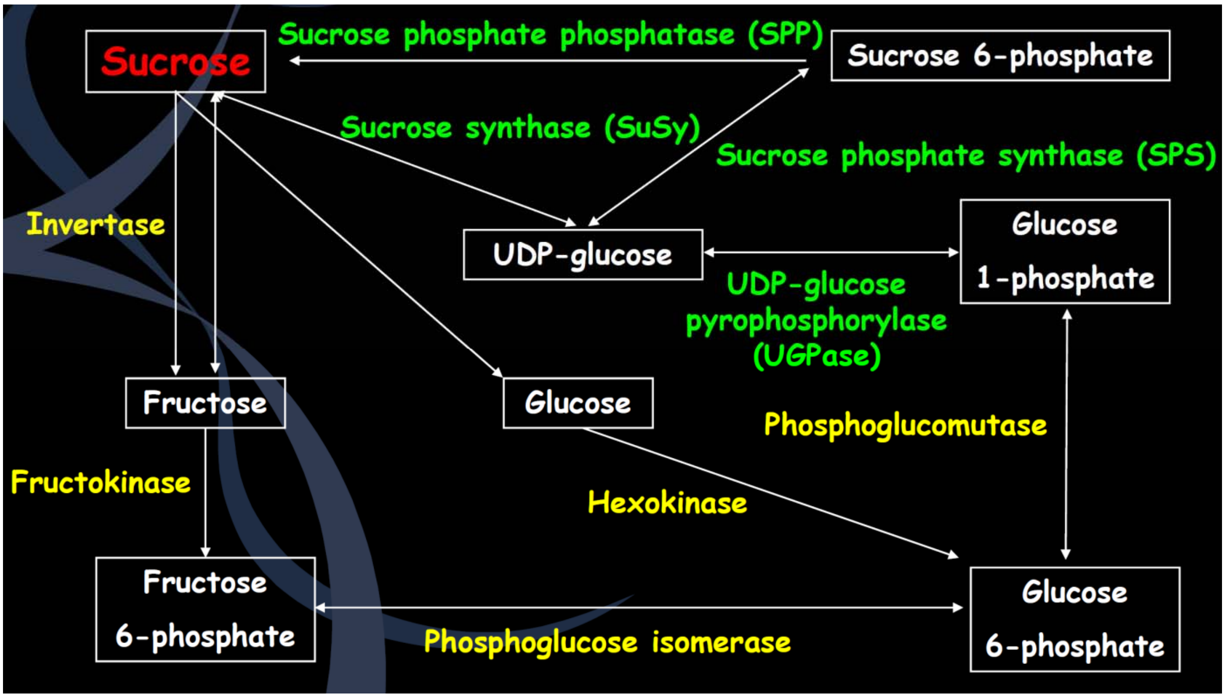

Supplementary Figure S1. Sucrose metabolism pathway. This pathway was modified according to the description in the reference (Grafahrend-Belau, E. *et al.* MetaCrop: a detailed database of crop plant metabolism, Nucleic Acids Res., **36 (Database issue)**, D954-D958 (2008)). At least 9 enzymes have been involved in sucrose metabolism.

Supplementary Figure S2A.

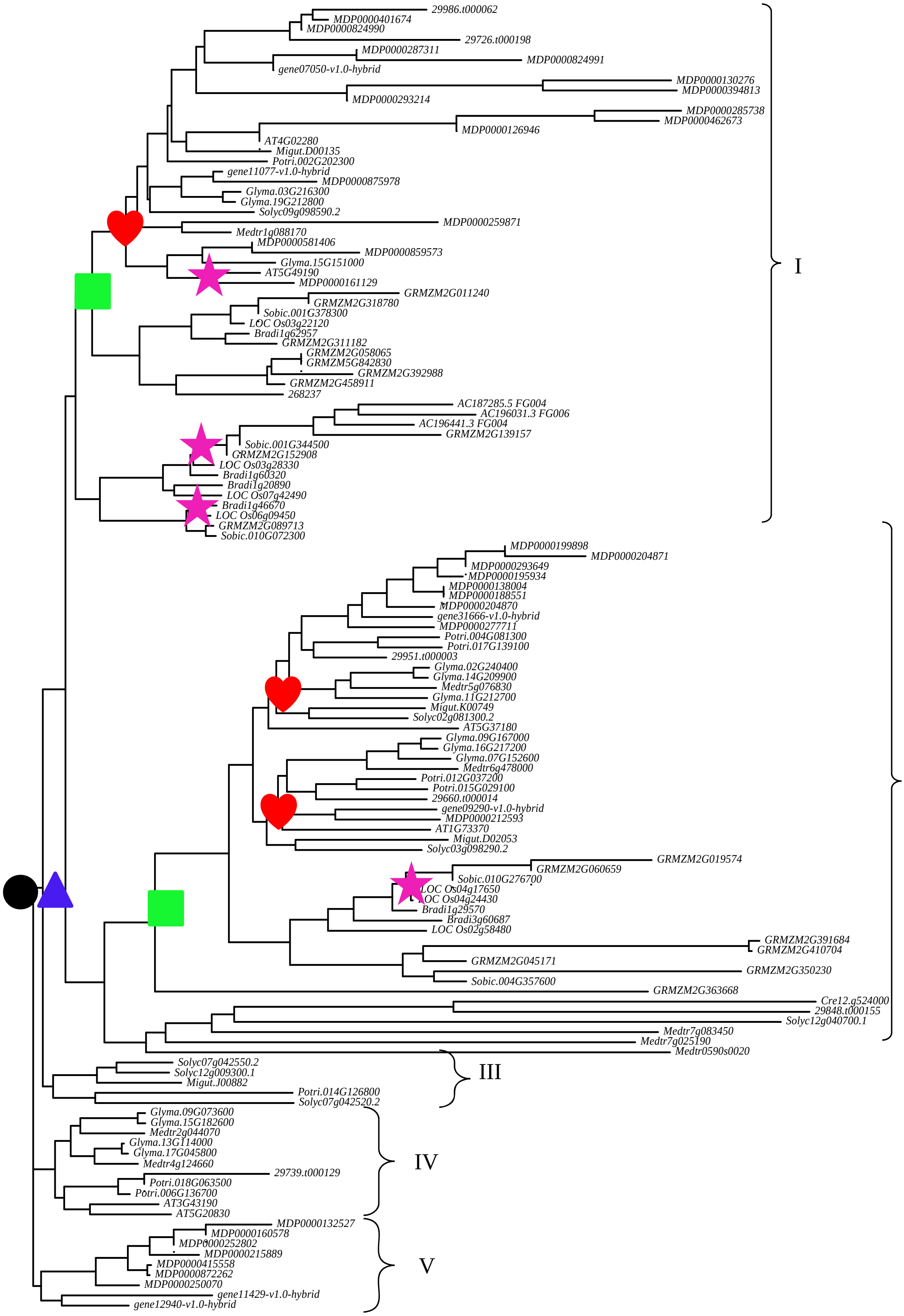

Supplementary Figure S2B.

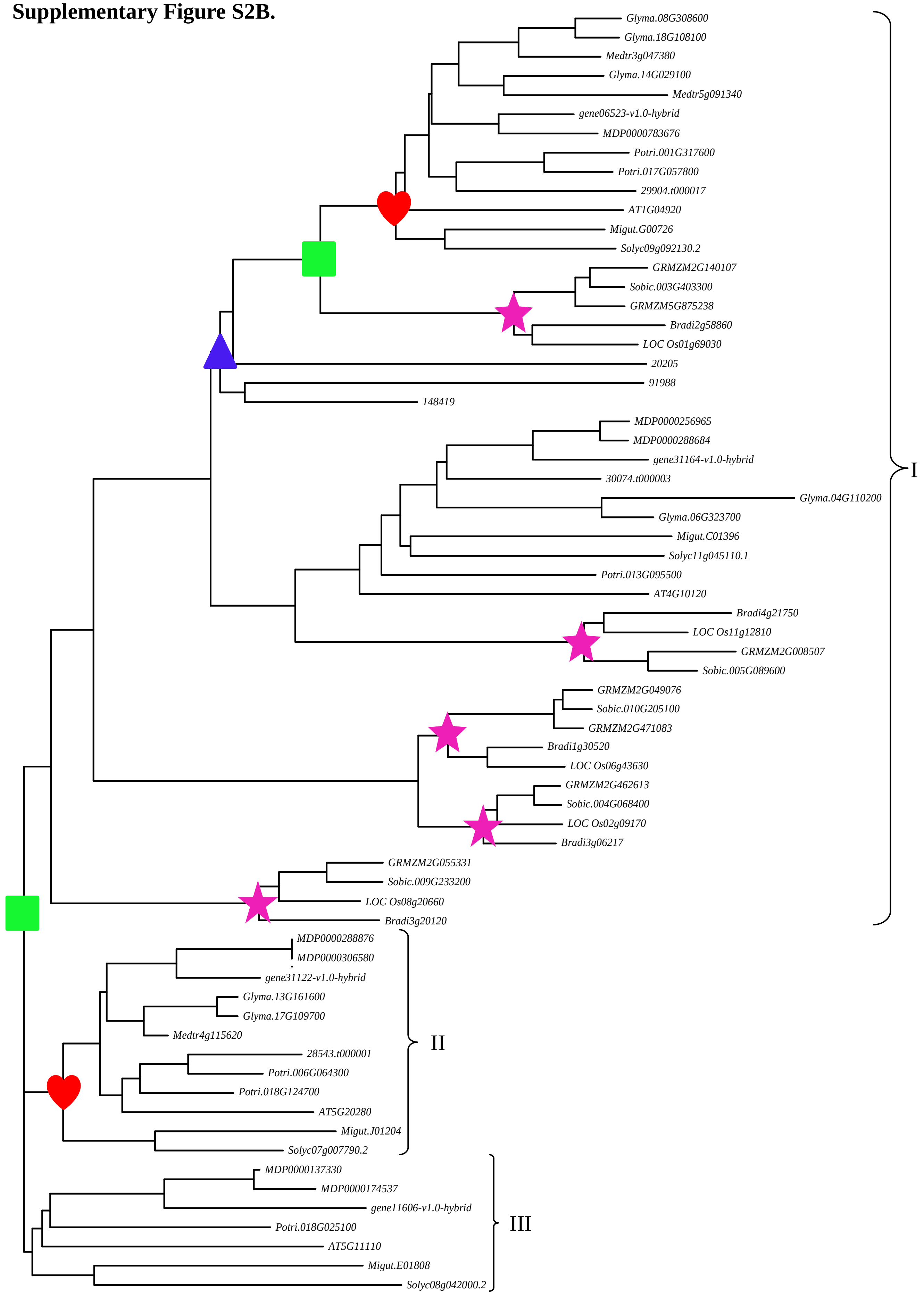

Supplementary Figure S2C.

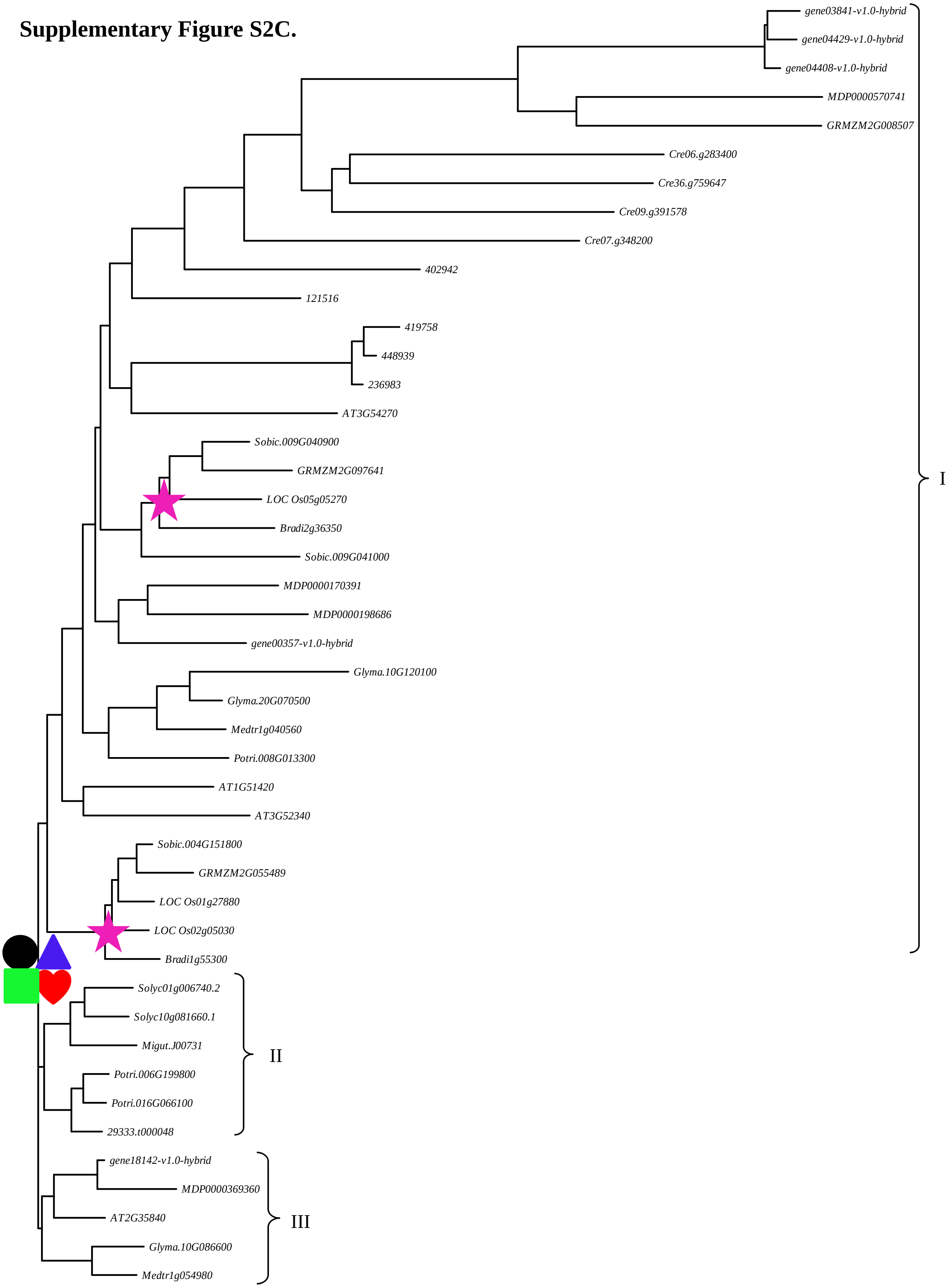

Supplementary Figure S2D.

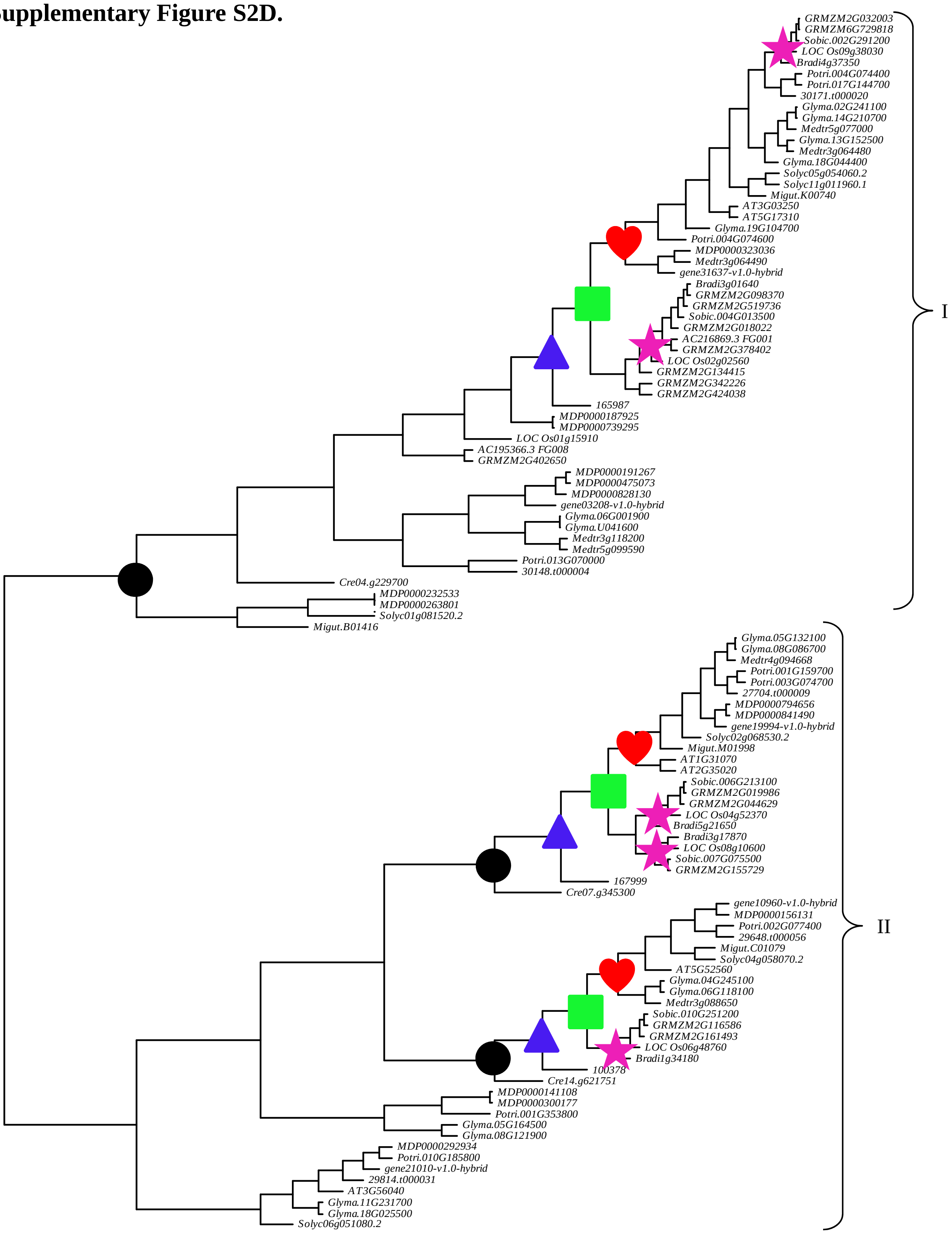

Supplementary Figure S2. Enlarged phylogenetic analyses of the SuSy **(A)**, SPS **(B)**, SPP **(C)** and UDPGP **(D)** families. Domain amino acid sequences from 15 species including 4 monocot and 9 dicot plants as well as 1 spikemoss and 1 algaewere employed to construct phylogenetic trees using the bootstrap method with a heuristic search of the PAUP 4.0b8 program. The results were confirmed by the Bayesian analyses. We defined ancestral units according to the description in this study.

Supplementary Figure S3

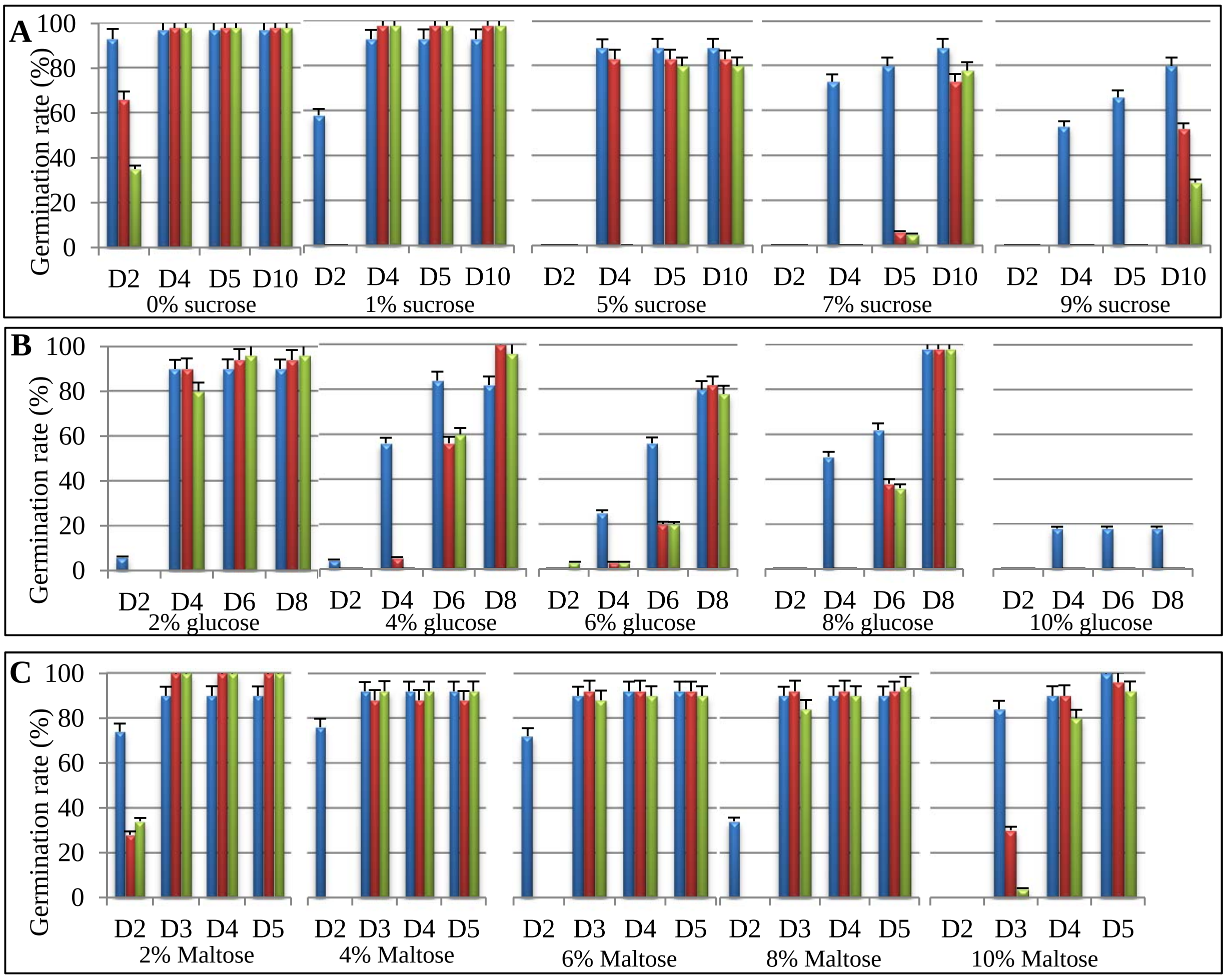

Supplementary Figure S3. Effects of various sugar stresses on seed germination of transgenic plants overexpressing *Sobic.009G233200*. **(A)** to **(C)** Effects of sucrose, glucose and maltose treatments on germination rates of WT and transgenic plants, respectively. Blue, red and green bars indicated WT, transgenic line 1 and line 2, respectively. D2, D3, D4, D5, D6, D8 and D10 indicated 2, 3, 4, 5, 6, 8 and 10 days after inoculation on 1/2 MS media.

Supplementary Figure S4

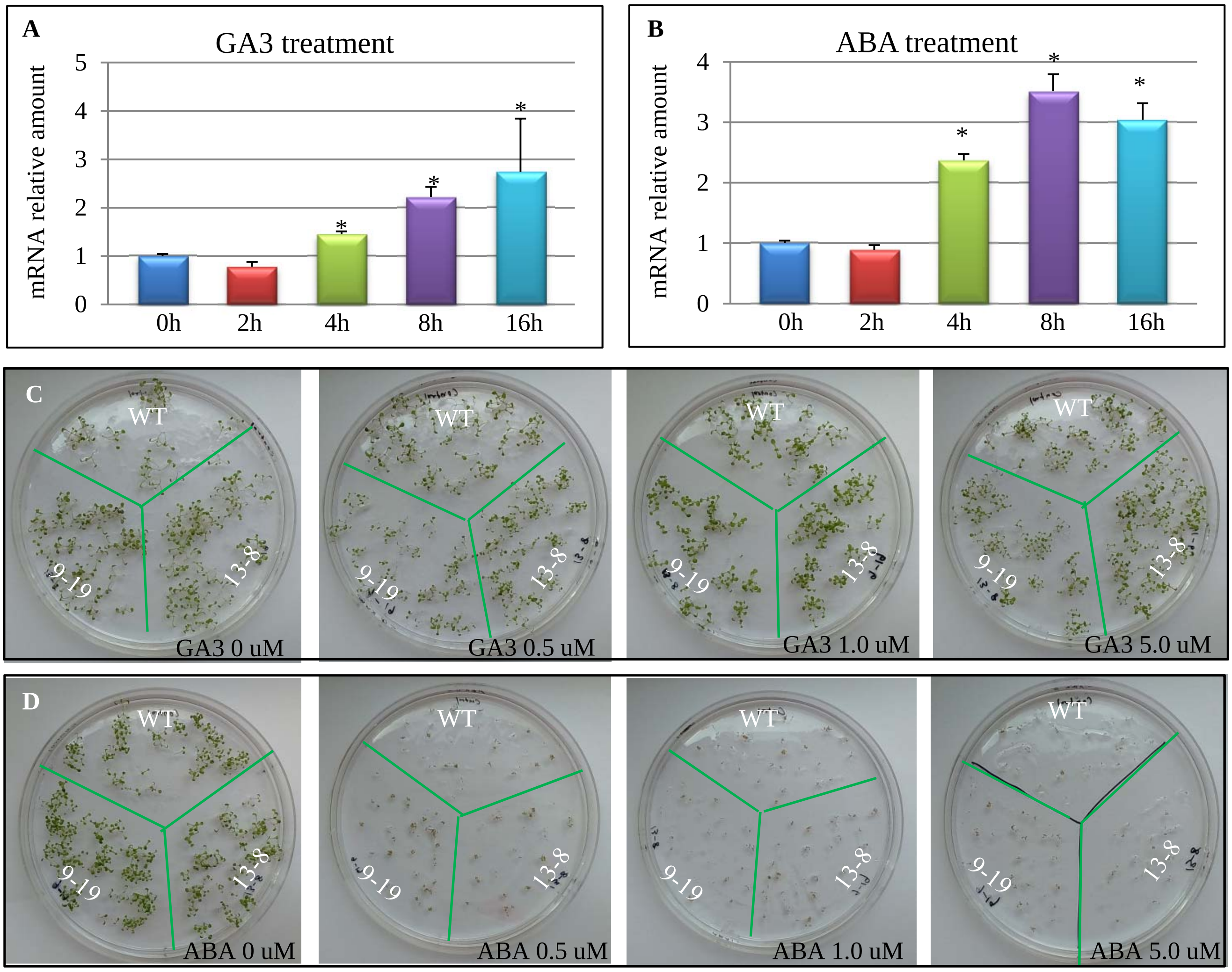

Supplementary Figure S4. Expression regulation of *Sobic.009G233200* in sorghum under Gibberellic acid (GA) and abscisic acid (ABA) and their effects on seed germination in transgenic Arabidopsis plants. **(A)** and **(B)** qRT-PCR analysis of the *SPS* gene *Sobic.009G233200* under GA3 and ABA treatments, respectively. Two-week-old seedlings were subjected to 100 uM GA3 or ABA treatments followed by qRT-PCR analysis using total RNA samples prepared from different time points of seedling samples. The stars "*" (A) and (B) indicated statistical difference at $P<0.05$ in expression level between control (0h) and treatments. **C)** and **(D)** Phenotypic observation of both WT and transgenic seeds overexpressing *Sobic.009G233200 (SPS)* under various concentrations of GA3 and ABA treatments, respectively. WT and transgenic Arabidopsis seeds were germinated on MS media supplemented with various concentrations of GA3 or ABA. Photos were taken after 9 days of germination.

Supplementary Figure S5

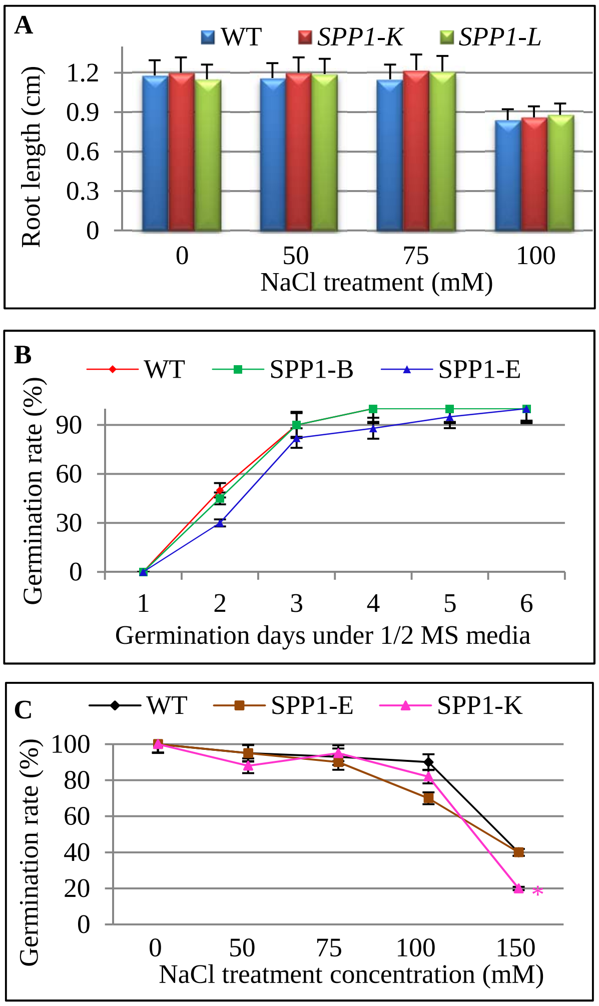

Supplementary Figure S5. Phenotypic characterization of transgenic plants overexpressing *Sobic.004G151800* (*SPP1*). **(A)** Measurement of root length under salinity stress in both WT and transgenic plants. **(B)** Investigation of germination rates between WT and transgenic plants under normal growth conditions. **(C)** Effects of high salinity treatments on germination rate in transgenic plants. The star "*" in **(C)** indicated significant difference in germination rates between WT and transgenic plants by statistic analysis.

Supplementary Figure S6

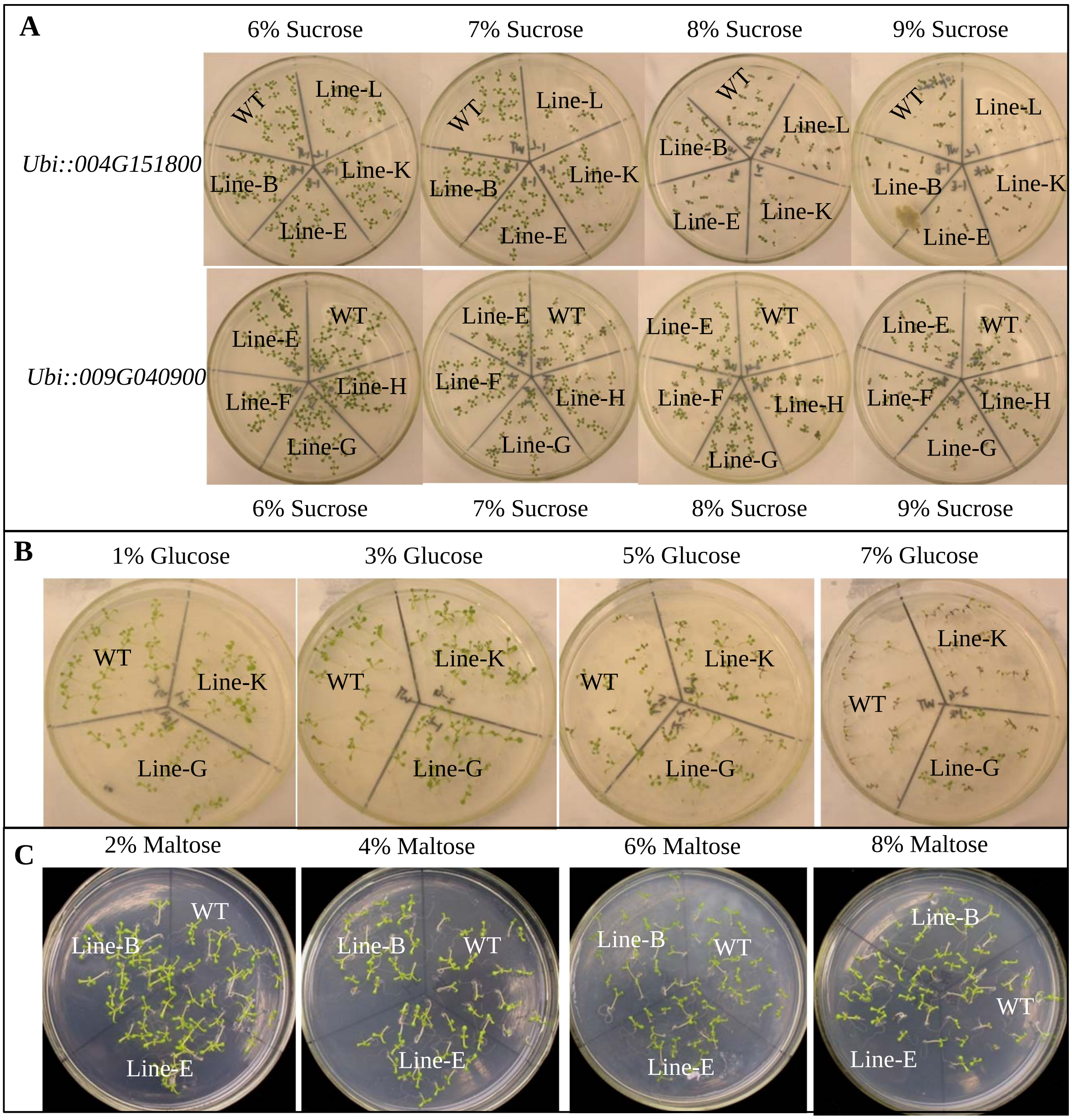

Supplementary Figure S6. Phenotypic investigation of transgenic plants by overexpressing sorghum gene *Sobic.004G151800* or *Sobic.009G040900* under sugar treatment. **(A)** The effect of sucrose treatment on seed germination and seedling development. The prefix "Sobic." of a sorghum gene locus name was omitted in each gene for convenience. Ubi: the maize ubiquitin promoter. **(B)** The effect of glucose treatment on seed germination and seedling development. Line-K and line-G, transgenic lines from *Ubi::004G151800* and *Ubi::009G040900*, respectively. **(C)** The effect of maltose treatment on seed germination and seedling development. Line-B and line-E, transgenic lines from *Ubi::004G151800* and *Ubi::009G040900*, respectively. All the pictures were taken after 7-day growth on ½ MS media.

Supplementary Figure S7

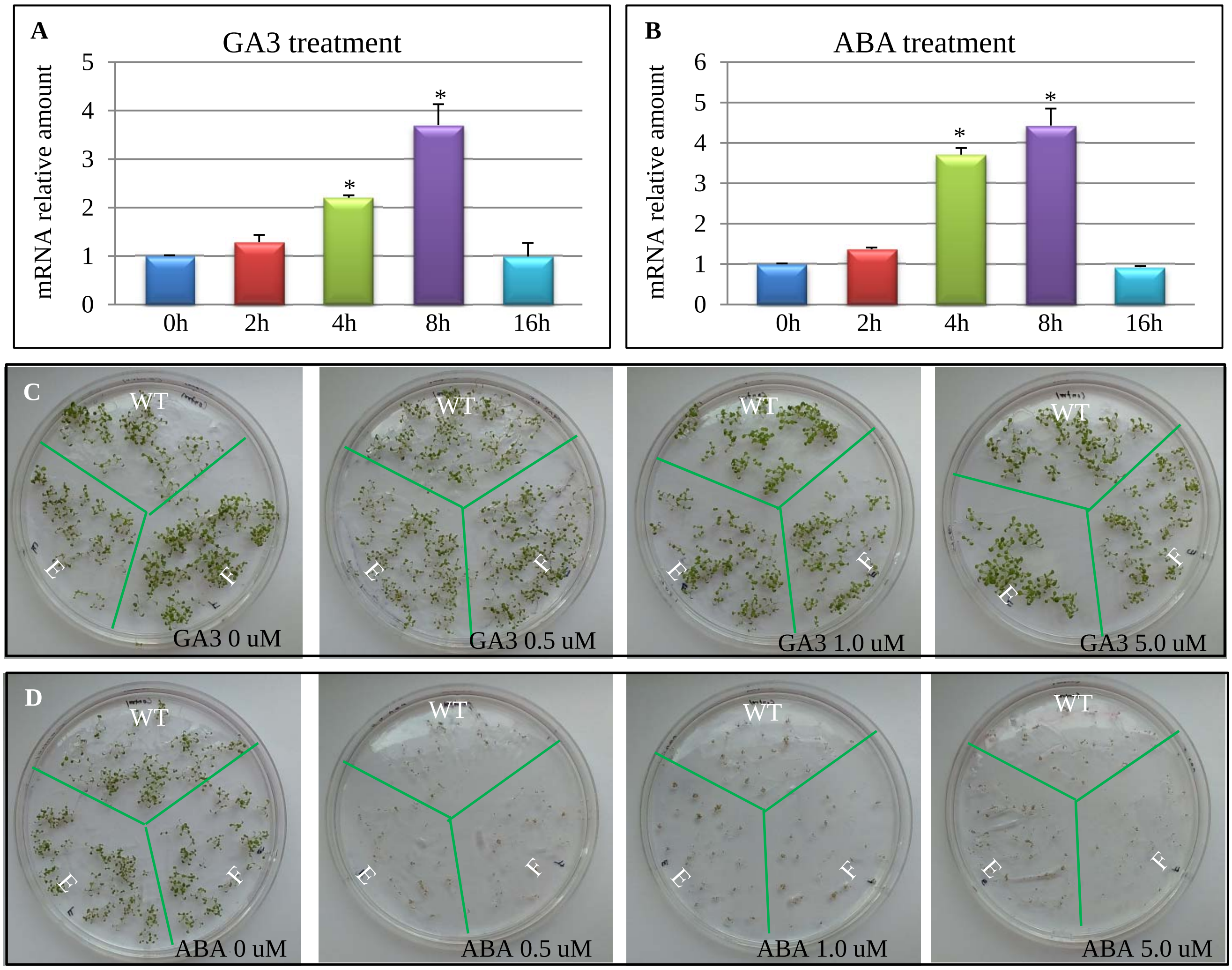

Supplementary Figure S7. Expression regulation of *Sobic.009G040900* in sorghum under Gibberellic acid (GA) and abscisic acid (ABA) and their effects on seed germination in transgenic Arabidopsis plants. **(A)** and **(B)** qRT-PCR analysis of the *SPP* gene *Sobic.009G040900* under GA3 and ABA treatments, respectively. Two-week-old seedlings were subjected to 100 uM GA3 or ABA treatments followed by qRT-PCR analysis using total RNA samples prepared from different time points of seedling samples. The stars "*" (A) and (B) indicated statistical difference at $P<0.05$ in expression level between control (0h) and treatments. **(C)** and **(D)** Phenotypic observation of both WT and transgenic seeds overexpressing *Sobic.009G040900 (SPP)* under various concentrations of GA3 and ABA treatments, respectively. WT and transgenic Arabidopsis seeds were germinated on MS media supplemented with various concentrations of GA3 or ABA. Photos were taken after 9 days of germination.

Supplementary Figure S8

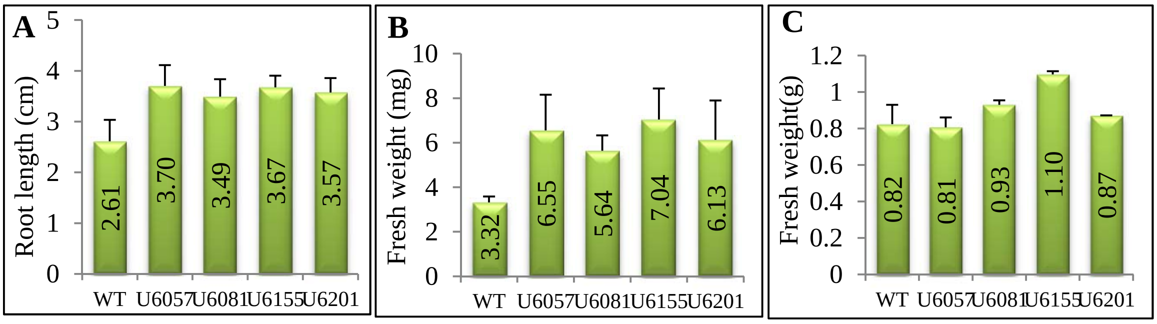

Supplementary Figure S8. Phenotypic characterization of transgenic plants overexpressing *Sobic.006G213100* in Arabidopsis. **(A)** Root length of 4 independent lines by comparing with WT plants after 8-day growth on ½ MS media. **(B)** Biomass analysis of 4 independent lines after 15-day growth on ½ MS media. **(C)** Biomass analysis of 4 independent lines after 65-day growth on ½ MS media.

Supplementary Figure S9

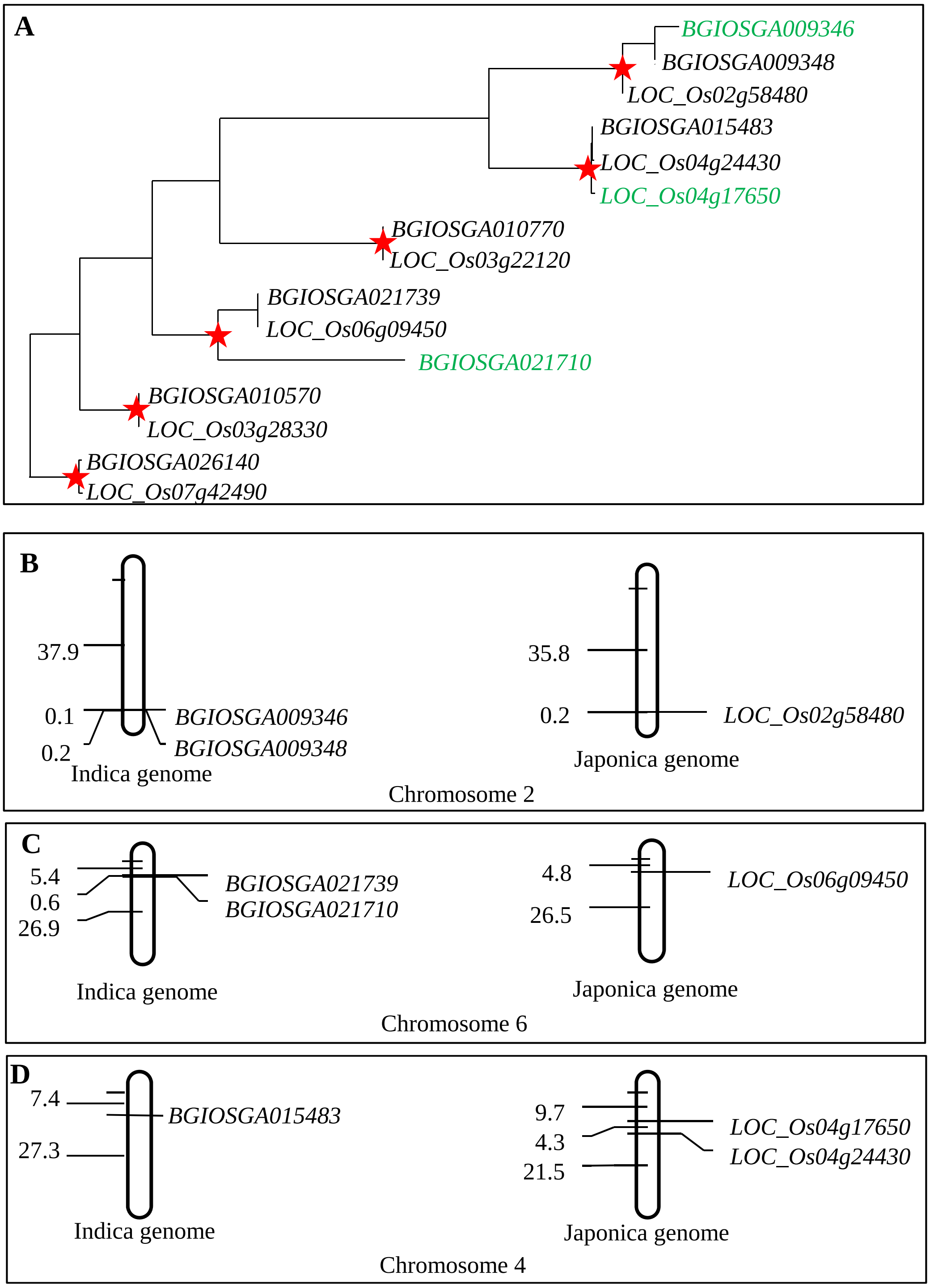

Supplementary Figure S9. Differentiated expansion of the *SuSy* gene family between indica and japonica genomes. **(A)** Phylogenetic tree based on all the SuSy members from both indica and japonica rice. Domain amino acid sequences were employed to construct phylogenetic trees using the bootstrap method with a heuristic search of the PAUP 4.0b8 program. Gene names with green fonts indicated that the gene was expanded only in either indica or japonica. The red stars indicated the MRCA units of the *SuSy* gene family between indica and japonica genomes. Indica genes begin with BGIOSGA and japonica genes begin with LOC_Os. **(B)** to **(C)** Differentially expanded genes by tandem duplication between indica and japonica and their chromosome localization.

Supplementary Table S1. Primer sequences used for this study

Targeted gene	Forward Primer	Reverse Primer	Remark
Sobic.004G151800	ATGCAAAGGAAGCCTCTT	GATTCAGCCTTTAGAACCAA	Expression analysis by RT-PCR
Sobic.004G151800	GCGGATCCATGGATAAGCTCAGTGGT	GCGAATTCCTACAGCTTAGACGCCTGCTC	Coding region amplification
Sobic.009G040900	GCGGATCCATGGATAGGCTCGACGGT	GCGAATTCCTACAAGATGAACGTGT	Coding region amplification
Sobic.009G040900	GCTCAATTCATACGAGAAACCA	TGGAGTCTCAGGCTTGATATT	Expression analysis by RT-PCR
Sobic.009G041000	GTTAAGTATCATCAATCCTGC	CAGGAGTGTCTGACTTCATATTC	Expression analysis by RT-PCR
Sobic.001G378300	ATGTCTGCCCCGAAGCTGGACC	CTACTGTGGCTGGTCAATTGCAAG	Promoter amplification
Sobic.001G344500	TTACTTCTGCAGCCCatgggggaagctgccggt	ACTAGTGGATCCCCCtcacttgctggagggctct	Coding region amplification
Sobic.001G378300	TTACTTCTGCAGCCCatgtctgccccgaagct	ACTAGTGGATCCCCCctactgtggctggtcaatt	Coding region amplification
Sobic.010G072300	TTACTTCTGCAGCCCatggctgccaagttgact	ACTAGTGGATCCCCCctagtcgtaggacaagg	Coding region amplification
Sobic.010G276700	TTACTTCTGCAGCCCatggcctccaagctgagt	ACTAGTGGATCCCCCctattgttgagaagtagaag	Coding region amplification
Sobic.004G357600	GCGGATCCCGAGGCGCTGCGTCAG	GCGAATTCTGGACAGCCGACAGCCCCTCA[illegible]	Coding region amplification
Sobic.001G378300	CATTCAATGCCTCAGTCCCACG	GCTGTATCACCCCAACCTTTCT	Expression analysis by RT-PCR
Sobic.002G291200	GCGGATCCTGCTCCCTCGCCACACC	GCGAATTCTCGAGCACCATGCATGTACC	Coding region amplification
Sobic.002G291200	GAGATTCCAGATGGAGCTGTAC	GACGGCGTTACCTAGGGAATATG	Expression analysis by RT-PCR
Sobic.004G013500	GCGGATCCGCGGGTAACGGGCTGTC	GCACTAGTTGCTGGGACAAGATTCTTTG	Coding region amplification
Sobic.004G013500	CTGAGGACCTTTAGATGGGAATC	GAACTGGATATAGAGCTACTGGAAC	Expression analysis by RT-PCR
Sobic.006G213100	GCGGATCCCGCCCGGTATATAAACGC	GCACTAGTTACACAACTGCTGCTCAGCTG	Coding region amplification
Sobic.006G213100	TGCTACTCCGCCTCCATAGCAG	CCACGGCTTATCAGCCAAATGAAC	Expression analysis by RT-PCR
Sobic.007G075500	AGGGAATATGGGCATCTGCCGGC	GGCTTCTAGATTCTCTCCAGCATAAG	Coding region amplification
Sobic.007G075500	CCATGCACCAAGTGAGATTTCATTC	GTGATGTGCAAGTGTTACCTTACCTG	Expression analysis by RT-PCR
Sobic.010G251200	CTTCCTCGCCCACGATGGCTTCG	GGAAGCTACTGGTCAATCAAGCAAATC	Coding region amplification
Sobic.001G378300	TTGTGCCTCCCTGGCTATAC	TGCCTGAAGACAAATTGCAG	qRT-PCR
Sobic.001G344500	GTTTCCTGGATGGGATGTTG	CTGCAATGTGAACCACAAGG	qRT-PCR
Sobic.006G213100	TTGCTTCACCTGTGTTGCTC	CATTTGAGAGCCATTCAGCA	qRT-PCR
Sobic.010G251200	GCAAAGTGCTTCACCAATCA	TTGCTTGATTCAGTGCCAAG	qRT-PCR
Sobic.010G205100	CATTCTAGGAGGGGTGCCTT	CCACAAGACAGGTTGGAAAAA	qRT-PCR

Supplementary Table S1. Primer sequences used for this study (to be continued)

Targeted gene	Forward Primer	Reverse Primer	Remark
Sobic.010G072300	TTCATTCCGACTGATGGTGA	TGGCACAGCAACCTTTGATA	qRT-PCR
Sobic.010G276700	ATGCATGATCTCCTGAAGCC	CCATCCATCTCTCCAATCAA	qRT-PCR
Sobic.009G233200	TTGCAAGACTCAGTGATGGC	CAGATCTCCGCACTGCAATA	qRT-PCR
Sobic.009G041000	TCACATCAGTCGTCTCCTGC	TGATGCTATGGTCAATTCTCTGA	qRT-PCR
Sobic.009G040900	ATTGCTGCCGCACTACTCTT	CTCCCTGCAGAGTGTCCTTC	qRT-PCR
Sobic.004G357600	CTTCATTTCAGGAAGCTGGC	TCTTTCTTTTGGCCTTGGTG	qRT-PCR
Sobic.004G013500	TCCATTCCTCTGAAATTGGG	TGCCCCTTTTGAACTGGATA	qRT-PCR
Sobic.004G151800	GGAGAGGATTTGGTGCGTAA	GATATTAGGTTGGGGCCGTT	qRT-PCR
Sobic.004G068400	AGCAAAAATGGTTTGGAACG	GCCACGGTAACTACCCACAT	qRT-PCR
Sobic.007G075500	AGGTTTGCACCATCACCTGT	TGGAATGACTGGAATGACCA	qRT-PCR
Sobic.002G291200	AGTGAAAGGGCACAAAATGG	ATTCGAACAAGCAGGAGCAT	qRT-PCR
Sobic.003G403300	GAACAGTACCGCGGGTGTAT	CAATCTTGTATGCTCGGGCT	qRT-PCR
Sobic.005G089600	CCACACTCTGCTGGGGTTAT	AGCCCTGTTTTCTGTCGCTA	qRT-PCR
HPT	ATGGTGAAGCCTGCAGCCGTC	TTAATACAACCAATTTGTACTTTTAGC	Probe
ACT1	TGTTTCCTAGTATTGTGGGTCGTC	CAGCGCATCCGTGAGGTC	Control for expression analysis in transgenic lines
SbUBQ5	ACCACTTCGACCGCCACTACT	GCGATTACGCCTTCTGGTT	an internal control for qRT-PCR

Supplementary Table S2. Genome-wide identification of *SuSy* genes in 50 sequenced genomes

Organism Name	Chromosome Name	Strand	Gene Name	Gene Start (bp)	Gene End (bp)
Chlamydomonas reinhardtii	chromosome_12	1	*Cre12.g524000*	4752775	4766776
CsubellipsoideaC_169	scaffold_11	1	*estExt_fgenesh1_pg.C_110186*	1243454	1261780
CsubellipsoideaC_169	scaffold_21	-1	*fgenesh1_pm.21_#_36*	674944	681514
P. patens	Chr01	-1	*Phpat.001G136000*	23598095	23603736
P. patens	Chr05	1	*Phpat.005G072500*	13944790	13951419
P. patens	Chr10	1	*Phpat.010G044300*	7610372	7615598
P. patens	Chr19	-1	*Phpat.019G020100*	3269871	3275682
S. moellendorffii	scaffold_40	1	*268237*	543660	547339
A. coerulea	scaffold_11	-1	*Aquca_011_00009*	376138	378410
A. coerulea	scaffold_2	-1	*Aquca_002_01365*	9804747	9810320
A. coerulea	scaffold_20	-1	*Aquca_020_00566*	3914125	3918631
A. coerulea	scaffold_3	1	*Aquca_003_00666*	8468592	8474693
A. coerulea	scaffold_48	-1	*Aquca_048_00019*	1276105	1278377
A. coerulea	scaffold_59	1	*Aquca_059_00018*	297984	300194
A. halleri	Scaffold11605	1	*Araha.11605s0001*	796	6041
A. halleri	Scaffold12632	-1	*Araha.12632s0001*	3569	7358
A. halleri	Scaffold16265	1	*Araha.16265s0001*	1788	6193
A. halleri	Scaffold23785	-1	*Araha.23785s0005*	16089	20949
A. halleri	Scaffold38479	-1	*Araha.38479s0008*	23509	27605
A. halleri	Scaffold6394	1	*Araha.6394s0003*	15784	18466
A. lyrata	scaffold_2	-1	*316284*	15792582	15796418
A. lyrata	scaffold_5	-1	*484788*	10006834	10011904
A. lyrata	scaffold_6	-1	*941635*	8825947	8829399
A. lyrata	scaffold_6	-1	*490342*	23811624	23815771
A. lyrata	scaffold_7	-1	*493691*	19838177	19842815
A. lyrata	scaffold_8	-1	*494980*	12784912	12788825
A. thaliana	Chr1	-1	*AT1G73370*	27584425	27588464
A. thaliana	Chr3	-1	*AT3G43190*	15179020	15183989
A. thaliana	Chr4	1	*AT4G02280*	994927	998967
A. thaliana	Chr5	-1	*AT5G20830*	7050226	7054120
A. thaliana	Chr5	1	*AT5G37180*	14718238	14722913
A. thaliana	Chr5	-1	*AT5G49190*	19943282	19947189
A. trichopoda	AmTr_v1.0_scaffold00044	-1	*evm_27.TU.AmTr_v1.0_scaffold00044.21*	420024	427185
A. trichopoda	AmTr_v1.0_scaffold00106	-1	*evm_27.TU.AmTr_v1.0_scaffold00106.5*	80681	87957
B. rapa	A03	-1	*Brara.C00954*	4450582	4454736
B. rapa	A05	-1	*Brara.E01375*	8601077	8605848
B. rapa	A06	1	*Brara.F03015*	24153728	24157985
B. rapa	A07	1	*Brara.G02318*	19835972	19839883
B. rapa	A07	1	*Brara.G02800*	22543825	22545785
B. rapa	A07	1	*Brara.G03267*	25091028	25094772
B. rapa	A09	1	*Brara.I00137*	786338	790678
B. rapa	A10	1	*Brara.J01546*	13464462	13469451
B. stricta	Scaffold10273	1	*Bostr.10273s0020*	135339	139287
B. stricta	Scaffold15774	-1	*Bostr.15774s0050*	781202	785567
B. stricta	Scaffold16335	-1	*Bostr.16335s0023*	163479	167194
B. stricta	Scaffold16335	-1	*Bostr.16335s0025*	175746	180773
B. stricta	Scaffold16335	-1	*Bostr.16335s0026*	183677	187563
B. stricta	Scaffold26527	1	*Bostr.26527s0127*	838451	842632
B. stricta	Scaffold556	-1	*Bostr.0556s0667*	4209383	4213851
B. stricta	Scaffold9638	1	*Bostr.9638s0039*	352868	358839
C. clementina	scaffold_1	1	*Ciclev10010343m.g*	24769195	24772566
C. clementina	scaffold_1	-1	*Ciclev10007483m.g*	24814539	24820057
C. clementina	scaffold_3	-1	*Ciclev10018889m.g*	46011238	46017249
C. clementina	scaffold_6	1	*Ciclev10011062m.g*	21404402	21408079
C. clementina	scaffold_9	1	*Ciclev10004341m.g*	1499634	1505333
C. grandiflora	Scaffold13232	-1	*Cagra.13232s0001*	896	4760
C. grandiflora	Scaffold2328	-1	*Cagra.2328s0002*	10123	12479
C. grandiflora	Scaffold3134	-1	*Cagra.3134s0017*	84399	89145
C. grandiflora	Scaffold4894	1	*Cagra.4894s0013*	60828	64784

Supplementary Table S2. Genome-wide identification of *SuSy* genes in 50 sequenced genomes (to be continued)

Organism Name	Chromosome Name	Strand	Gene Name	Gene Start (bp)	Gene End (bp)
C. grandiflora	Scaffold6431	1	*Cagra.6431s0002*	12097	15650
C. grandiflora	Scaffold753	-1	*Cagra.0753s0010*	36658	40104
C. papaya	contig_29609	1	*evm.TU.contig_29609*	7075	11827
C. papaya	supercontig_151	-1	*evm.TU.supercontig_151.21*	177298	184954
C. papaya	supercontig_178	-1	*evm.TU.supercontig_178.35*	313413	317811
C. papaya	supercontig_217	-1	*evm.TU.supercontig_217.19*	229216	235701
C. papaya	supercontig_50	1	*evm.TU.supercontig_50.160*	1526912	1532495
C. papaya	supercontig_82	1	*evm.TU.supercontig_82.65*	1095128	1098675
C. rubella	scaffold_2	-1	*Carubv10019738m.g*	11145808	11149800
C. rubella	scaffold_5	-1	*Carubv10016702m.g*	5228102	5231651
C. rubella	scaffold_6	-1	*Carubv10000208m.g*	7074984	7078886
C. rubella	scaffold_6	-1	*Carubv10000249m.g*	15685055	15689230
C. rubella	scaffold_7	1	*Carubv10006192m.g*	14629575	14634893
C. rubella	scaffold_8	-1	*Carubv10025867m.g*	5803325	5807967
C. sativus	scaffold00919	1	*Cucsa.096950*	2398136	2403454
C. sativus	scaffold00953	-1	*Cucsa.112470*	1031520	1035774
C. sativus	scaffold01079	-1	*Cucsa.142060*	378520	383413
C. sativus	scaffold01209	1	*Cucsa.173660*	336069	341436
C. sinensis	scaffold00001	1	*orange1.1g003492m.g*	4880666	4887107
C. sinensis	scaffold00013	1	*orange1.1g003661m.g*	1377734	1382723
C. sinensis	scaffold00013	-1	*orange1.1g003947m.g*	1422592	1426092
C. sinensis	scaffold00016	-1	*orange1.1g003726m.g*	362782	368287
C. sinensis	scaffold00091	1	*orange1.1g036539m.g*	79725	83042
C. sinensis	scaffold00674	1	*orange1.1g002909m.g*	65197	69161
E. grandis	Chr02	-1	*Eucgr.B01577*	22603562	22608737
E. grandis	Chr03	1	*Eucgr.C00769*	13006267	13012052
E. grandis	Chr03	1	*Eucgr.C03199*	64344566	64348440
E. grandis	Chr03	1	*Eucgr.C03201*	64383343	64387905
E. grandis	Chr03	1	*Eucgr.C03204*	64409314	64412913
E. grandis	Chr03	1	*Eucgr.C03205*	64435446	64439168
E. grandis	Chr03	1	*Eucgr.C03207*	64476971	64481348
E. grandis	Chr06	1	*Eucgr.F01010*	14252073	14256271
E. grandis	Chr08	1	*Eucgr.H01094*	12669981	12679293
E. grandis	Chr08	1	*Eucgr.H03496*	48052732	48056940
E. grandis	Chr08	1	*Eucgr.H03515*	48520676	48524230
E. grandis	Chr10	1	*Eucgr.J01640*	20610185	20614355
E. grandis	Chr11	1	*Eucgr.K00816*	9668792	9672040
E. grandis	Chr11	1	*Eucgr.K02305*	30949634	30954626
E. grandis	Chr11	-1	*Eucgr.K03505*	43483154	43489403
E. salsugineum	scaffold_14	1	*Thhalv10027639m.g*	8203443	8208035
E. salsugineum	scaffold_2	1	*Thhalv10012643m.g*	3598450	3602812
E. salsugineum	scaffold_2	-1	*Thhalv10012719m.g*	7199478	7204709
E. salsugineum	scaffold_3	1	*Thhalv10028435m.g*	1073258	1077482
E. salsugineum	scaffold_6	1	*Thhalv10003667m.g*	4709550	4713485
E. salsugineum	scaffold_9	1	*Thhalv10018094m.g*	3196714	3200705
F. vesca	LG1	-1	*gene11429-v1.0-hybrid*	4790008	4802072
F. vesca	LG1	-1	*gene12940-v1.0-hybrid*	6534488	6537776
F. vesca	LG1	1	*gene31666-v1.0-hybrid*	10093857	10097563
F. vesca	LG2	-1	*gene11077-v1.0-hybrid*	16107092	16112349
F. vesca	LG4	-1	*gene07050-v1.0-hybrid*	20160747	20165599
F. vesca	LG5	-1	*gene09290-v1.0-hybrid*	9513235	9523248
G. max	Chr02	1	*Glyma.02G240400*	42892680	42898279
G. max	Chr03	-1	*Glyma.03G216300*	42037913	42044153
G. max	Chr07	-1	*Glyma.07G152600*	18519523	18521811
G. max	Chr09	-1	*Glyma.09G073600*	7809992	7816199
G. max	Chr09	-1	*Glyma.09G167000*	39103764	39109664
G. max	Chr11	-1	*Glyma.11G212700*	30547238	30552421
G. max	Chr13	-1	*Glyma.13G114000*	22767704	22773231
G. max	Chr14	1	*Glyma.14G209900*	47515899	47521687
G. max	Chr15	-1	*Glyma.15G151000*	12497126	12504804
G. max	Chr15	-1	*Glyma.15G182600*	17910131	17916426

Supplementary Table S2. Genome-wide identification of *SuSy* genes in 50 sequenced genomes (to be continued)

Organism Name	Chromosome Name	Strand	Gene Name	Gene Start (bp)	Gene End (bp)
G. max	Chr16	-1	*Glyma.16G217200*	37414228	37419838
G. max	Chr17	1	*Glyma.17G045800*	3404918	3410491
G. max	Chr19	-1	*Glyma.19G212800*	46633685	46639818
G. raimondii	Chr01	-1	*Gorai.001G017700*	1633649	1638690
G. raimondii	Chr01	-1	*Gorai.001G083400*	8827369	8831426
G. raimondii	Chr04	-1	*Gorai.004G142700*	40279285	40284709
G. raimondii	Chr07	-1	*Gorai.007G047100*	3273020	3276978
G. raimondii	Chr09	-1	*Gorai.009G038000*	2794061	2797932
G. raimondii	Chr10	1	*Gorai.010G091800*	14533649	14537174
G. raimondii	Chr10	-1	*Gorai.010G092300*	14665936	14670130
G. raimondii	Chr13	1	*Gorai.013G222400*	54230175	54234656
L. usitatissimum	scaffold123	1	*Lus10041979.g*	212432	217497
L. usitatissimum	scaffold133	1	*Lus10001468.g*	80059	85126
L. usitatissimum	scaffold149	1	*Lus10012454.g*	259027	262359
L. usitatissimum	scaffold157	1	*Lus10008204.g*	26616	27018
L. usitatissimum	scaffold157	1	*Lus10008205.g*	27156	29013
L. usitatissimum	scaffold230	1	*Lus10013417.g*	114063	118050
L. usitatissimum	scaffold302	1	*Lus10007372.g*	211098	214756
L. usitatissimum	scaffold303	1	*Lus10020791.g*	703184	706944
L. usitatissimum	scaffold44	-1	*Lus10020506.g*	747271	750680
L. usitatissimum	scaffold687	1	*Lus10017984.g*	215843	220928
L. usitatissimum	scaffold732	-1	*Lus10010308.g*	128448	132511
M. domestica	MDC000687.579	-1	*MDP0000462673*	1148	1342
M. domestica	MDC000971.390	-1	*MDP0000824990*	782	1042
M. domestica	MDC000971.390	-1	*MDP0000824991*	1196	1607
M. domestica	MDC002418.288	1	*MDP0000859573*	1372	2253
M. domestica	MDC002418.292	-1	*MDP0000161129*	1751	6367
M. domestica	MDC002776.293	1	*MDP0000160578*	3603	7039
M. domestica	MDC005119.811	1	*MDP0000277711*	140	8871
M. domestica	MDC009859.246	-1	*MDP0000285738*	3128	4412
M. domestica	MDC010568.273	-1	*MDP0000188551*	2948	6852
M. domestica	MDC010658.589	-1	*MDP0000138004*	5466	9370
M. domestica	MDC010779.409	-1	*MDP0000287311*	5311	10299
M. domestica	MDC010818.345	1	*MDP0000250070*	28226	31888
M. domestica	MDC012015.232	-1	*MDP0000581406*	59	2725
M. domestica	MDC012052.408	-1	*MDP0000293214*	4936	9560
M. domestica	MDC012304.216	1	*MDP0000126946*	14164	19499
M. domestica	MDC012545.277	1	*MDP0000204870*	1228	4959
M. domestica	MDC012545.277	1	*MDP0000204871*	7004	10868
M. domestica	MDC012545.291	-1	*MDP0000199898*	6	3267
M. domestica	MDC012545.293	1	*MDP0000293649*	72	4130
M. domestica	MDC012545.297	-1	*MDP0000195934*	41	2664
M. domestica	MDC012905.242	-1	*MDP0000401674*	106	279
M. domestica	MDC013646.294	-1	*MDP0000875978*	5453	6128
M. domestica	MDC014107.433	-1	*MDP0000394813*	10796	11089
M. domestica	MDC015207.315	-1	*MDP0000872262*	6808	10265
M. domestica	MDC015369.215	1	*MDP0000252802*	1922	4886
M. domestica	MDC015369.234	-1	*MDP0000415558*	2844	6301
M. domestica	MDC016112.100	1	*MDP0000212593*	30420	34488
M. domestica	MDC016795.84	1	*MDP0000259871*	3246	4656
M. domestica	MDC018143.375	1	*MDP0000130276*	4335	5088
M. domestica	MDC021971.120	-1	*MDP0000215889*	2465	5901
M. domestica	MDC021971.181	-1	*MDP0000132527*	6661	10100
M. esculenta	scaffold01945	1	*cassava4.1_026466m.g*	445017	451285
M. esculenta	scaffold03264	-1	*cassava4.1_001283m.g*	428169	432574
M. esculenta	scaffold03975	1	*cassava4.1_001871m.g*	229806	235130
M. esculenta	scaffold04457	1	*cassava4.1_001840m.g*	279819	286827
M. esculenta	scaffold07520	-1	*cassava4.1_027790m.g*	503583	507435
M. esculenta	scaffold09702	-1	*cassava4.1_001874m.g*	31494	36022
M. esculenta	scaffold10114	1	*cassava4.1_001867m.g*	126431	131442
M. guttatus	scaffold_10	1	*Migut.J00882*	5309732	5315109

Supplementary Table S2. Genome-wide identification of *SuSy* genes in 50 sequenced genomes (to be continued)

Organism Name	Chromosome Name	Strand	Gene Name	Gene Start (bp)	Gene End (bp)
M. guttatus	scaffold_11	-1	*Migut.K00749*	5922455	5927403
M. guttatus	scaffold_4	1	*Migut.D00135*	614650	618812
M. guttatus	scaffold_4	1	*Migut.D02053*	17739045	17742886
M. truncatula	chr1	1	*Medtr1g088170*	39395678	39401597
M. truncatula	chr2	-1	*Medtr2g044070*	19151293	19157303
M. truncatula	chr3	-1	*Medtr3g064610*	29116823	29122075
M. truncatula	chr4	1	*Medtr4g124660*	51648884	51653878
M. truncatula	chr4	-1	*Medtr4g133550*	55866365	55867138
M. truncatula	chr5	1	*Medtr5g076830*	32773395	32778891
M. truncatula	chr6	1	*Medtr6g478000*	28772794	28779662
M. truncatula	chr6	1	*Medtr6g478030*	28792237	28799904
M. truncatula	chr7	-1	*Medtr7g025190*	8348938	8349222
M. truncatula	chr7	-1	*Medtr7g025200*	8354575	8358751
M. truncatula	chr7	1	*Medtr7g077170*	29143346	29144285
M. truncatula	chr7	-1	*Medtr7g083450*	32093272	32099019
M. truncatula	chr7	-1	*Medtr7g106150*	43123223	43124039
M. truncatula	chr7	-1	*Medtr7g108930*	44513813	44520053
M. truncatula	scaffold0590	-1	*Medtr0590s0020*	5019	7798
P. hallii	scaffold_210	1	*Pahal.0210s0038*	209167	213628
P. hallii	scaffold_24	1	*Pahal.0024s0429*	2623795	2627774
P. hallii	scaffold_426	-1	*Pahal.0426s0015*	81840	87910
P. hallii	scaffold_49	-1	*Pahal.0049s0140*	1108752	1113143
P. hallii	scaffold_56	-1	*Pahal.0056s0208*	1521849	1533615
P. patens	Chr01	-1	*Pp3c1_33460*	23598154	23603738
P. patens	Chr05	1	*Pp3c5_19770*	13944801	13951470
P. patens	Chr10	1	*Pp3c10_11330*	7610372	7615598
P. patens	Chr19	-1	*Pp3c19_5850*	3269919	3275670
P. persica	scaffold_1	1	*ppa001845m.g*	9912782	9917014
P. persica	scaffold_1	1	*ppa001573m.g*	9918131	9923492
P. persica	scaffold_3	1	*ppa017606m.g*	149555	153309
P. persica	scaffold_5	1	*ppa001135m.g*	18201753	18206006
P. persica	scaffold_7	1	*ppa001535m.g*	18751831	18757844
P. persica	scaffold_8	-1	*ppa002723m.g*	21434978	21439332
P. trichocarpa	Chr02	1	*Potri.002G202300*	16415744	16422183
P. trichocarpa	Chr04	-1	*Potri.004G081300*	6705500	6709825
P. trichocarpa	Chr06	1	*Potri.006G136700*	11285440	11290774
P. trichocarpa	Chr12	1	*Potri.012G037200*	3315693	3319802
P. trichocarpa	Chr14	1	*Potri.014G126800*	9738844	9740178
P. trichocarpa	Chr15	1	*Potri.015G029100*	2355672	2360072
P. trichocarpa	Chr17	1	*Potri.017G139100*	14732088	14736491
P. trichocarpa	Chr18	-1	*Potri.018G063500*	7953559	7957693
P. vulgaris	Chr01	-1	*Phvul.001G209600*	47309963	47315951
P. vulgaris	Chr03	1	*Phvul.003G127500*	31014499	31019973
P. vulgaris	Chr04	-1	*Phvul.004G142800*	42207786	42213699
P. vulgaris	Chr06	-1	*Phvul.006G087300*	20615139	20619848
P. vulgaris	Chr08	-1	*Phvul.008G241300*	55557595	55562802
P. vulgaris	Chr09	1	*Phvul.009G223800*	33128754	33134026
P. vulgaris	Chr09	-1	*Phvul.009G250800*	36376972	36384586
R. communis	29660	1	*29660.t000014*	105100	109047
R. communis	29726	-1	*29726.t000198*	1126767	1131842
R. communis	29739	1	*29739.t000129*	806117	811347
R. communis	29848	1	*29848.t000155*	898924	903741
R. communis	29951	1	*29951.t000003*	78434	83092
R. communis	29986	-1	*29986.t000062*	402754	408435
S. lycopersicum	SL2.40ch02	-1	*Solyc02g081300.2*	39893591	39897878
S. lycopersicum	SL2.40ch03	1	*Solyc03g098290.2*	54082963	54087172
S. lycopersicum	SL2.40ch07	-1	*Solyc07g042520.2*	53140157	53143939
S. lycopersicum	SL2.40ch07	-1	*Solyc07g042550.2*	53300389	53305994
S. lycopersicum	SL2.40ch09	1	*Solyc09g098590.2*	67652867	67658231
S. lycopersicum	SL2.40ch12	-1	*Solyc12g009300.1*	2573935	2577879
S. lycopersicum	SL2.40ch12	-1	*Solyc12g040700.1*	40295366	40298524

Supplementary Table S2. Genome-wide identification of *SuSy* genes in 50 sequenced genomes (to be continued)

Organism Name	Chromosome Name	Strand	Gene Name	Gene Start (bp)	Gene End (bp)
S. polyrhiza	pseudo0	1	*Spipo0G0124100*	8526416	8531273
S. polyrhiza	pseudo1	1	*Spipo1G0051200*	3317980	3321738
S. polyrhiza	pseudo18	1	*Spipo18G0002500*	313711	316455
S. polyrhiza	pseudo18	-1	*Spipo18G0002900*	361845	367021
S. polyrhiza	pseudo2	1	*Spipo2G0031600*	2867518	2872698
S. polyrhiza	pseudo20	-1	*Spipo20G0008000*	775661	779195
S. polyrhiza	pseudo3	-1	*Spipo3G0031400*	1880371	1883249
S. purpurea	chr02	-1	*SapurV1A.0494s0030*	18727262	18733391
S. purpurea	chr04	1	*SapurV1A.1584s0040*	8982329	8987081
S. purpurea	chr06	1	*SapurV1A.0249s0050*	12679932	12687684
S. purpurea	chr13	-1	*SapurV1A.2015s0010*	11985057	11992085
S. purpurea	chr15	1	*SapurV1A.0074s0060*	3935280	3939486
S. purpurea	chr17	1	*SapurV1A.0549s0120*	16811120	16815684
S. purpurea	chr18	1	*SapurV1A.0121s0110*	5763110	5770370
S. purpurea	Scaffold5061	-1	*SapurV1A.5061s0010*	1450	5975
S. tuberosum	chr02	-1	*PGSC0003DMG400016730*	58194476	58197232
S. tuberosum	chr07	1	*PGSC0003DMG400013546*	38512004	38518368
S. tuberosum	chr07	1	*PGSC0003DMG400013547*	38546068	38549700
S. tuberosum	chr12	1	*PGSC0003DMG400002895*	3087152	3091456
T. aestivum	ta_iwgsc_1al_v2_1028742	-1	*Traes_1AL_AAF8D8497*	8	1664
T. aestivum	ta_iwgsc_2al_v1_6333913	-1	*Traes_2AL_8E8343BA5*	395	4875
T. aestivum	ta_iwgsc_2as_v1_5216970	1	*Traes_2AS_F2967D6F7*	3637	7546
T. aestivum	ta_iwgsc_2bl_v1_8026696	1	*Traes_2BL_C963272C8*	1584	7743
T. aestivum	ta_iwgsc_2bs_v1_5178880	-1	*Traes_2BS_96ECE84C2*	1991	7956
T. aestivum	ta_iwgsc_2dl_v1_9853726	1	*Traes_2DL_22482812B*	9880	13321
T. aestivum	ta_iwgsc_2ds_v1_5360578	-1	*Traes_2DS_ECBFB4D8C*	403	6531
T. aestivum	ta_iwgsc_3b_v1_10458486	-1	*Traes_3B_55B913FD2*	1	4097
T. aestivum	ta_iwgsc_3dl_v1_6951494	1	*Traes_3DL_A2EB73B1D*	466	3257
T. aestivum	ta_iwgsc_4al_v2_2713402	-1	*Traes_4AL_2BC235062*	1	5085
T. aestivum	ta_iwgsc_4as_v2_5984059	-1	*Traes_4AS_EF48BBCCF*	2389	13537
T. aestivum	ta_iwgsc_4bl_v1_6986922	-1	*Traes_4BL_D1AA966DE*	333	3091
T. aestivum	ta_iwgsc_4bl_v1_7007100	-1	*Traes_4BL_B49C2A51C*	5488	7741
T. aestivum	ta_iwgsc_4dl_v3_14349308	-1	*Traes_4DL_F08462AD2*	2730	5982
T. aestivum	ta_iwgsc_4dl_v3_14460089	1	*Traes_4DL_9F7C8C343*	1599	4053
T. aestivum	ta_iwgsc_5al_v1_2708214	-1	*Traes_5AL_DC621E0E3*	3	6222
T. aestivum	ta_iwgsc_6al_v1_5742333	-1	*Traes_6AL_02ECEFFB7*	7710	12583
T. aestivum	ta_iwgsc_6as_v1_4373103	1	*Traes_6AS_A00425DAD1*	6305	10168
T. aestivum	ta_iwgsc_6as_v1_4382840	-1	*Traes_6AS_A00425DAD*	1	3864
T. aestivum	ta_iwgsc_6bl_v1_4220015	-1	*Traes_6BL_FC54B7E8F*	5141	8017
T. aestivum	ta_iwgsc_6dl_v1_3210843	-1	*Traes_6DL_3C0C05516*	8069	10828
T. aestivum	ta_iwgsc_6ds_v1_2092108	-1	*Traes_6DS_573CAD5E0*	3	4216
T. aestivum	ta_iwgsc_7al_v1_4536617	-1	*Traes_7AL_3F2C16688*	5446	10216
T. aestivum	ta_iwgsc_7as_v1_4255196	-1	*Traes_7AS_2742DDF6C*	5079	11601
T. aestivum	ta_iwgsc_7as_v1_4255448	-1	*Traes_7AS_5D84FA56B*	104	2939
T. aestivum	ta_iwgsc_7as_v1_4256374	-1	*Traes_7AS_A80BE362A*	1	1783
T. aestivum	ta_iwgsc_7bl_v1_6538036	-1	*Traes_7BL_612EB6214*	511	5166
T. aestivum	ta_iwgsc_7bl_v1_6751305	-1	*Traes_7BL_FBEAF8C41*	1218	5910
T. aestivum	ta_iwgsc_7bs_v1_3131167	-1	*Traes_7BS_182F2A1F1*	4713	7144
T. aestivum	ta_iwgsc_7dl_v1_3364136	-1	*Traes_7DL_471B4134B*	3956	9268
T. aestivum	ta_iwgsc_7ds_v1_3893149	-1	*Traes_7DS_529BAB150*	257	5376
T. aestivum	ta_iwgsc_7ds_v1_3920687	1	*Traes_7DS_7094F3B4D*	5389	7846
T. cacao	scaffold_1	-1	*Thecc1EG004698*	33027559	33035420
T. cacao	scaffold_3	1	*Thecc1EG013507*	13708969	13714009
T. cacao	scaffold_4	-1	*Thecc1EG017585*	5066782	5086791
T. cacao	scaffold_4	-1	*Thecc1EG021253*	32351689	32357321
T. cacao	scaffold_9	1	*Thecc1EG037464*	3617395	3622520
T. cacao	scaffold_9	-1	*Thecc1EG037468*	3647921	3653541
V. vinifera	chr11	1	*GSVIVG01015018001*	490468	494415
V. vinifera	chr17	1	*GSVIVG01029388001*	15994779	15999389
V. vinifera	chr4	1	*GSVIVG01035210001*	10519232	10523018
V. vinifera	chr5	1	*GSVIVG01035106001*	1507786	1515522

Supplementary Table S2. Genome-wide identification of *SuSy* genes in 50 sequenced genomes (to be continued)

Organism Name	Chromosome Name	Strand	Gene Name	Gene Start (bp)	Gene End (bp)
V. vinifera	chr7	-1	*GSVIVG01028043001*	3380955	3389790
B. distachyon	Bd1	1	*Bradi1g20890*	16788479	16793794
B. distachyon	Bd1	1	*Bradi1g29570*	25158577	25163567
B. distachyon	Bd1	1	*Bradi1g46670*	45456402	45462514
B. distachyon	Bd1	1	*Bradi1g60320*	59905229	59910530
B. distachyon	Bd1	1	*Bradi1g62957*	62434219	62441460
B. distachyon	Bd3	1	*Bradi3g60687*	59616729	59621589
O. sativa	Chr2	1	*LOC_Os02g58480*	35754946	35761781
O. sativa	Chr3	-1	*LOC_Os03g22120*	12674462	12680657
O. sativa	Chr3	-1	*LOC_Os03g28330*	16301277	16306143
O. sativa	Chr4	-1	*LOC_Os04g17650*	9661934	9667057
O. sativa	Chr4	1	*LOC_Os04g24430*	14008245	14013440
O. sativa	Chr6	-1	*LOC_Os06g09450*	4796286	4802578
O. sativa	Chr7	1	*LOC_Os07g42490*	25429639	25435182
P. virgatum	Chr01b	1	*Pavir.Ab03343*	55176001	55180160
P. virgatum	Chr04a	1	*Pavir.Da01892*	42481187	42486619
P. virgatum	Chr04b	-1	*Pavir.Db02070*	45426643	45429853
P. virgatum	Chr09a	-1	*Pavir.Ia03182*	64736564	64743134
P. virgatum	Chr09a	1	*Pavir.Ia03415*	68412314	68418248
P. virgatum	Chr09b	-1	*Pavir.Ib01597*	17292865	17294916
P. virgatum	Chr09b	-1	*Pavir.Ib01813*	19841448	19848852
P. virgatum	contig02078	1	*Pavir.J01866*	14151	19020
P. virgatum	contig21457	1	*Pavir.J19702*	3841	8072
P. virgatum	contig228853	1	*Pavir.J21013*	223	1981
P. virgatum	contig30433	-1	*Pavir.J27132*	3983	6194
P. virgatum	contig78675	1	*Pavir.J38773*	110	4363
P. virgatum	contig99610	-1	*Pavir.J41086*	130	3156
S. bicolor	Chr01	1	*Sobic.001G344500*	56130406	56137242
S. bicolor	Chr01	1	*Sobic.001G378300*	59452296	59460141
S. bicolor	Chr04	1	*Sobic.004G357600*	67754723	67758468
S. bicolor	Chr10	-1	*Sobic.010G072300*	5810039	5816046
S. bicolor	Chr10	-1	*Sobic.010G276700*	60830698	60835335
S. italica	scaffold_1	1	*Si020148m.g*	41988015	41991404
S. italica	scaffold_4	1	*Si005859m.g*	2940746	2949224
S. italica	scaffold_4	-1	*Si005845m.g*	40061875	40065988
S. italica	scaffold_9	1	*Si034282m.g*	43202415	43208087
S. italica	scaffold_9	1	*Si034293m.g*	46886615	46892456
Z. mays	1	-1	*GRMZM2G410704*	17721176	17724401
Z. mays	1	-1	*GRMZM2G311182*	56758337	56763169
Z. mays	1	-1	*GRMZM2G011240*	56763340	56766439
Z. mays	1	-1	*GRMZM2G318780*	56787028	56789894
Z. mays	1	1	*GRMZM2G350230*	226979838	226981216
Z. mays	1	-1	*AC196441.3_FG004*	272267690	272268393
Z. mays	2	1	*GRMZM2G058065*	24027192	24028824
Z. mays	2	1	*GRMZM2G391684*	146834859	146839335
Z. mays	4	-1	*GRMZM2G019574*	66969155	66969914
Z. mays	4	-1	*GRMZM2G045171*	168773364	168776492
Z. mays	5	-1	*GRMZM2G060659*	64797860	64801915
Z. mays	6	1	*GRMZM2G363668*	29392045	29395597
Z. mays	7	1	*AC196031.3_FG006*	117388784	117392188
Z. mays	8	1	*AC187285.5_FG004*	106527529	106529403
Z. mays	8	1	*GRMZM2G392988*	124361368	124364917
Z. mays	9	1	*GRMZM5G842830*	1106705	1107826
Z. mays	9	1	*GRMZM2G089713*	11500945	11506749
Z. mays	9	-1	*GRMZM2G152908*	122479052	122485725
Z. mays	10	1	*GRMZM2G458911*	31602674	31603881
Z. mays	10	1	*GRMZM2G139157*	84614500	84621081

Supplementary Table S3. Genome-wide identification of *SPS* genes in 50 sequenced genomes

Organism Name	Chromosome Name	Strand	Gene Name	Gene Start (bp)	Gene End (bp)
S. moellendorffii	scaffold_4	1	20205	1233296	1237663
S. moellendorffii	scaffold_12	-1	91988	1370509	1374199
S. moellendorffii	scaffold_19	1	148419	786638	790712
A. lyrata	scaffold_1	-1	470477	1678193	1682519
A. lyrata	scaffold_6	1	487943	4449916	4454681
A. lyrata	scaffold_6	-1	488935	8579942	8585509
A. lyrata	scaffold_6	1	489859	20026862	20032113
R. communis	28543	1	28543.t000001	6207	13318
R. communis	29904	1	29904.t000017	120781	127015
R. communis	30074	1	30074.t000003	32607	39540
A. coerulea	scaffold_11	1	Aquca_011_00228	3434345	3444957
A. coerulea	scaffold_20	1	Aquca_020_00040	324227	330561
A. coerulea	scaffold_34	-1	Aquca_034_00099	863235	869025
A. coerulea	scaffold_114	-1	Aquca_114_00017	273220	280934
A. halleri	Scaffold10896	-1	Araha.10896s0004	7755	12233
A. halleri	Scaffold12073	-1	Araha.12073s0006	20944	28438
A. halleri	Scaffold1252	1	Araha.1252s0005	19405	24922
A. halleri	Scaffold3305	-1	Araha.3305s0001	1	5121
A. thaliana	Chr1	-1	AT1G04920	1391502	1395864
A. thaliana	Chr4	1	AT4G10120	6314789	6319936
A. thaliana	Chr5	1	AT5G11110	3536226	3541133
A. thaliana	Chr5	-1	AT5G20280	6844714	6850154
B. stricta	Scaffold25219	1	Bostr.25219s0480	2192134	2199256
B. stricta	Scaffold25463	-1	Bostr.25463s0304	2545492	2550869
B. stricta	Scaffold26527	1	Bostr.26527s0171	1165789	1171691
B. distachyon	Bd1	-1	Bradi1g30520	25881941	25889855
B. distachyon	Bd2	-1	Bradi2g58860	56691342	56697005
B. distachyon	Bd3	1	Bradi3g06217	4486990	4495350
B. distachyon	Bd3	1	Bradi3g20120	19198456	19208103
B. distachyon	Bd4	1	Bradi4g21750	25419918	25425558
B. rapaFPsc	A02	-1	Brara.B00877	4121142	4126838
B. rapa	A02	1	Brara.B02532	17684287	17688976
B. rapa	A03	1	Brara.C00452	2134812	2139395
B. rapa	A06	1	Brara.F02728	22475221	22480184
B. rapa	A10	-1	Brara.J00308	1751056	1756207
B. rapa	A10	1	Brara.J01584	13667774	13673319
C. grandiflora	Scaffold179	-1	Cagra.0179s0012	39753	45617
C. grandiflora	Scaffold1671	-1	Cagra.1671s0111	426577	430813
C. grandiflora	Scaffold2761	-1	Cagra.2761s0005	15184	20071
C. grandiflora	Scaffold6692	-1	Cagra.6692s0007	18852	23766
C. rubella	scaffold_6	1	Carubv10000112m.g	13011205	13016636
C. rubella	scaffold_6	1	Carubv10000113m.g	3505601	3510431
C. rubella	scaffold_1	-1	Carubv10008160m.g	1468884	1473118
M. esculenta	scaffold12793	1	cassava4.1_000732m.g	116382	124592
M. esculenta	scaffold00847	-1	cassava4.1_000744m.g	1549109	1553611
M. esculenta	scaffold09788	-1	cassava4.1_000827m.g	97441	105505
M. esculenta	scaffold07595	1	cassava4.1_000839m.g	105359	112183
M. esculenta	scaffold03332	1	cassava4.1_024105m.g	878777	884984
C. clementina	scaffold_9	1	Ciclev10004221m.g	15981287	15989471
C. clementina	scaffold_1	-1	Ciclev10007311m.g	25880824	25887976
C. clementina	scaffold_1	1	Ciclev10007312m.g	1634245	1639542
C. clementina	scaffold_3	1	Ciclev10018655m.g	23001026	23009675
C. sativus	scaffold00888	-1	Cucsa.088480	768834	775469
C. sativus	scaffold02209	1	Cucsa.249370	113278	121251
C. sativus	scaffold02352	-1	Cucsa.266070	493174	499634
E. grandis	Chr03	-1	Eucgr.C01715	30041024	30047311
E. grandis	Chr03	-1	Eucgr.E03524	25091202	25098398
E. grandis	Chr06	-1	Eucgr.F02931	41473400	41482052

Supplementary Table S3. Genome-wide identification of *SPS* genes in 50 sequenced genomes (to be continued)

Organism Name	Chromosome Name	Strand	Gene Name	Gene Start (bp)	Gene End (bp)
E. grandis	Chr08	1	Eucgr.H00041	4175646	4183999
C. papaya	supercontig_142	-1	evm.TU.supercontig_142.9	54165	61663
C. papaya	supercontig_46	-1	evm.TU.supercontig_46.57	467907	475242
C. papaya	supercontig_83	-1	evm.TU.supercontig_83.22	281575	286949
A.trichopoda	AmTr_v1.0_scaffolc	-1	evm_27.TU.AmTr_v1.0_scaffold00033.17(	3827404	3850841
A.trichopoda	AmTr_v1.0_scaffolc	1	evm_27.TU.AmTr_v1.0_scaffold00099.85	1282420	1303191
F. vesca	LG4	-1	gene06523-v1.0-hybrid	16876681	16882157
F. vesca	unanchored	1	gene11606-v1.0-hybrid	9174890	9180436
F. vesca	LG1	-1	gene31122-v1.0-hybrid	2640599	2646376
F. vesca	LG2	1	gene31164-v1.0-hybrid	7925299	7930502
G. max	Chr04	1	Glyma.04G110200	12143112	12149594
G. max	Chr06	1	Glyma.06G323700	51145662	51154901
G. max	Chr08	1	Glyma.08G308600	42724400	42731453
G. max	Chr13	-1	Glyma.13G161600	27715861	27722364
G. max	Chr14	-1	Glyma.14G029100	2126633	2133000
G. max	Chr17	1	Glyma.17G109700	8602474	8609046
G. max	Chr18	-1	Glyma.18G108100	12308018	12314522
G. raimondii	Chr04	-1	Gorai.004G292800	62058128	62063615
G. raimondii	Chr09	1	Gorai.009G262100	21660522	21667243
G. raimondii	Chr10	-1	Gorai.010G116100	22615477	22621179
G. raimondii	Chr11	1	Gorai.011G155900	27189106	27195620
G. raimondii	Chr13	-1	Gorai.013G235900	55387209	55394483
Z. mays	4	-1	GRMZM2G008507	10486291	10492230
Z. mays	9	1	GRMZM2G049076	95338846	95357615
Z. mays	4	-1	GRMZM2G055331	65287980	65306249
Z. mays	3	1	GRMZM2G140107	161295516	161301138
Z. mays	5	1	GRMZM2G462613	96392076	96399771
Z. mays	6	1	GRMZM2G471083	97070014	97080958
Z. mays	8	1	GRMZM5G875238	161465833	161471194
V. vinifera	chr11	-1	GSVIVG01012825001	5766519	5776338
V. vinifera	chr5	-1	GSVIVG01020928001	16983726	16990038
V. vinifera	chr4	-1	GSVIVG01035882001	5217811	5232786
V. vinifera	chr18	1	GSVIVG01037186001	28208996	28216173
O. sativa	Chr1	-1	LOC_Os01g69030	40101404	40107104
O. sativa	Chr2	1	LOC_Os02g09170	4708493	4716880
O. sativa	Chr6	-1	LOC_Os06g43630	26242005	26250269
O. sativa	Chr8	1	LOC_Os08g20660	12411932	12424769
O. sativa	Chr11	-1	LOC_Os11g12810	7255766	7263164
L. usitatissimum	scaffold896	1	Lus10008056.g	275695	280891
L. usitatissimum	scaffold915	1	Lus10016657.g	495657	500636
L. usitatissimum	scaffold465	1	Lus10022570.g	809557	811833
L. usitatissimum	scaffold475	1	Lus10038119.g	1733203	1738910
L. usitatissimum	scaffold280	1	Lus10041041.g	677146	686075
M. domestica	MDC008037.203	1	MDP0000137330	2041	6764
M. domestica	MDC004632.398	-1	MDP0000174537	19489	24938
M. domestica	MDC006970.126	-1	MDP0000256965	5784	11369
M. domestica	MDC010427.133	-1	MDP0000288684	5953	11327
M. domestica	MDC008863.329	-1	MDP0000288876	1629	7105
M. domestica	MDC016631.38	-1	MDP0000306580	1629	7106
M. domestica	MDC001262.252	1	MDP0000783676	16461	21536
M. truncatula	chr3	1	Medtr3g047380	15793866	15803225
M. truncatula	chr4	-1	Medtr4g115620	47752792	47759989
M. truncatula	chr5	1	Medtr5g091340	39809072	39814155
M. guttatus	scaffold_3	1	Migut.C01396	18795230	18800065
M. guttatus	scaffold_5	-1	Migut.E01808	24196412	24200676
M. guttatus	scaffold_7	1	Migut.G00726	5173298	5180524
M. guttatus	scaffold_10	1	Migut.J01204	11630193	11637560
C. sinensis	scaffold02200	-1	orange1.1g001492m.g	1015	8972

Supplementary Table S3. Genome-wide identification of *SPS* genes in 50 sequenced genomes (to be continued)

Organism Name	Chromosome Name	Strand	Gene Name	Gene Start (bp)	Gene End (bp)
C. sinensis	scaffold00013	1	orange1.1g001541m.g	369034	375974
C. sinensis	scaffold00051	-1	orange1.1g001557m.g	618418	623689
C. sinensis	scaffold00603	1	orange1.1g001705m.g	39884	48183
P. hallii	scaffold_1	-1	Pahal.0001s0188	3183527	3189256
P. hallii	scaffold_78	-1	Pahal.0078s0109	713309	719629
P. hallii	scaffold_111	-1	Pahal.0111s0014	429900	439699
P. hallii	scaffold_111	-1	Pahal.0111s0066	1345841	1351194
P. hallii	scaffold_117	1	Pahal.0117s0038	320640	329193
P. virgatum	Chr01a	-1	Pavir.Aa03125	65570089	65577636
P. virgatum	Chr06a	1	Pavir.Fa00990	15265505	15273118
P. virgatum	Chr06b	-1	Pavir.Fb01074	25833017	25840490
P. virgatum	Chr08b	-1	Pavir.Hb01394	39182911	39187834
P. virgatum	contig08668	1	Pavir.J05550	1057	10560
P. virgatum	contig08865	-1	Pavir.J05632	5587	13476
P. virgatum	contig35764	1	Pavir.J30592	137	6282
P. virgatum	contig47741	1	Pavir.J34574	1	5683
P. virgatum	contig63414	1	Pavir.J36866	2	4901
S. tuberosum	chr09	1	PGSC0003DMG400026428	51280330	51289317
S. tuberosum	chr07	-1	PGSC0003DMG400027936	4385121	4393975
S. tuberosum	chr08	1	PGSC0003DMG400029892	19874594	19878226
S. tuberosum	chr11	1	PGSC0003DMG402019060	16237013	16243564
P. patens	Chr09	-1	Phpat.009G095500	17040135	17045667
P. patens	Chr15	-1	Phpat.015G092800	15582046	15589211
P. vulgaris	Chr03	-1	Phvul.003G170100	38063134	38070587
P. vulgaris	Chr05	-1	Phvul.005G002600	191956	199903
P. vulgaris	Chr06	1	Phvul.006G031700	13270984	13278092
P. trichocarpa	Chr01	-1	Potri.001G317600	32261122	32267596
P. trichocarpa	Chr06	1	Potri.006G064300	4724719	4731781
P. trichocarpa	Chr13	-1	Potri.013G095500	10293919	10304711
P. trichocarpa	Chr17	-1	Potri.017G057800	5245839	5251789
P. trichocarpa	Chr18	1	Potri.018G025100	1995179	2001769
P. trichocarpa	Chr18	1	Potri.018G124700	14774433	14782711
P. persica	scaffold_1	-1	ppa000622m.g	12254827	12260723
P. persica	scaffold_1	-1	ppa000636m.g	39315271	39321798
P. persica	scaffold_7	-1	ppa000639m.g	21553364	21558937
P. persica	scaffold_8	-1	ppa000716m.g	302823	308439
S. purpurea	chr18	1	SapurV1A.0034s0100	13522878	13531610
S. purpurea	chr18	1	SapurV1A.0034s0120	13539492	13543561
S. purpurea	chr13	-1	SapurV1A.0109s0050	11587818	11593038
S. purpurea	chr18	1	SapurV1A.0192s0260	2187318	2194744
S. purpurea	chr06	1	SapurV1A.0197s0290	4743642	4749193
S. purpurea	chr17	-1	SapurV1A.0412s0230	5039047	5046066
S. italica	scaffold_5	-1	Si000130m.g	44828497	44833956
S. italica	scaffold_4	1	Si005783m.g	32007425	32016125
S. italica	scaffold_6	-1	Si013170m.g	16670386	16680956
S. italica	scaffold_1	1	Si016229m.g	5697446	5705143
S. italica	scaffold_8	-1	Si025907m.g	9448362	9453752
S. bicolor	Chr03	-1	Sobic.003G403300	71135756	71141978
S. bicolor	Chr04	1	Sobic.004G068400	5592103	5599224
S. bicolor	Chr05	1	Sobic.005G089600	12955277	12961424
S. bicolor	Chr09	-1	Sobic.009G233200	57284131	57297240
S. bicolor	Chr10	-1	Sobic.010G205100	54483017	54493428
S. lycopersicum	SL2.40ch07	1	Solyc07g007790.2	2438924	2447625
S. lycopersicum	SL2.40ch08	-1	Solyc08g042000.2	28598289	28605520
S. lycopersicum	SL2.40ch09	-1	Solyc09g092130.2	66617015	66625125
S. lycopersicum	SL2.40ch11	-1	Solyc11g045110.1	36639062	36646330
S. polyrhiza	pseudo10	1	Spipo10G0053200	4214844	4221826
S. polyrhiza	pseudo13	1	Spipo13G0027900	1746611	1752896

Supplementary Table S3. Genome-wide identification of *SPS* genes in 50 sequenced genomes (to be continued)

Organism Name	Chromosome Name	Strand	Gene Name	Gene Start (bp)	Gene End (bp)
S. polyrhiza	pseudo7	-1	Spipo7G0045000	4100810	4104674
S. polyrhiza	pseudo9	1	Spipo9G0051300	3336068	3340324
T. cacao	scaffold_4	1	Thecc1EG020273	27608104	27615091
T. cacao	scaffold_8	1	Thecc1EG036061	15625629	15632373
T. cacao	scaffold_9	-1	Thecc1EG038167	7453565	7474773
T. cacao	scaffold_9	1	Thecc1EG046907	1171958	1178143
E. salsugineum	scaffold_5	1	Thhalv10006639m.g	14219701	14223755
E. salsugineum	scaffold_2	-1	Thhalv10012540m.g	6985604	6991362
E. salsugineum	scaffold_3	-1	Thhalv10028384m.g	7327439	7332564
T. aestivum	ta_iwgsc_3al_v1_42	-1	Traes_3AL_D3A1F0D64	664	5561
T. aestivum	ta_iwgsc_3as_v1_33	-1	Traes_3AS_6505083E0	2917	11220
T. aestivum	ta_iwgsc_3b_v1_103	1	Traes_3B_32D611152	67	5709
T. aestivum	ta_iwgsc_3b_v1_107	1	Traes_3B_35D6F6CE7	11	3367
T. aestivum	ta_iwgsc_3ds_v1_26	-1	Traes_3DS_9B89BAD5A	3719	11264
T. aestivum	ta_iwgsc_4bs_v1_49	-1	Traes_4BS_D4D9B5E3E	2192	8236
T. aestivum	ta_iwgsc_6bs_v1_29	1	Traes_6BS_9E57D74AC	1	7107
T. aestivum	ta_iwgsc_6ds_v1_93	1	Traes_6DS_DAA89D248	2	3050
T. aestivum	ta_iwgsc_7al_v1_45	-1	Traes_7AL_5F21F6932	1930	7198
T. aestivum	ta_iwgsc_7dl_v1_33	-1	Traes_7DL_9D207AC31	3661	6451

Supplementary Table S4. Genome-wide identification of *SPP* genes in 50 sequenced genomes

Organism Name	Chromosome Name	Strand	Gene Name	Gene Start (bp)	Gene End (bp)
S. moellendorffii	scaffold_65	-1	*121516*	455770	457331
S. moellendorffii	scaffold_329	1	*236983*	5567	7042
S. moellendorffii	scaffold_0	1	*402942*	5534224	5535832
S. moellendorffii	scaffold_43	-1	*419758*	1532640	1535771
S. moellendorffii	scaffold_144	1	*448939*	24769	29752
A. lyrata	scaffold_5	1	*485599*	16042026	16044304
A. lyrata	scaffold_1	-1	*914277*	27109867	27111732
A. lyrata	scaffold_4	1	*934210*	16982946	16984806
A. lyrata	scaffold_5	-1	*938346*	16931169	16933477
R. communis	29333	-1	*29333.t000048*	349361	353548
A. coerulea	scaffold_9	-1	*Aquca 009 00639*	3799195	3802565
A. coerulea	scaffold_9	-1	*Aquca 009 00640*	3804339	3808205
A. coerulea	scaffold_30	-1	*Aquca 030 00187*	1329657	1331121
A. coerulea	scaffold_30	1	*Aquca 030 00188*	1337204	1340662
A. halleri	Scaffold2044	1	*Araha.2044s0004*	24052	26122
A. halleri	Scaffold23752	1	*Araha.23752s0004*	23092	25701
A. halleri	Scaffold4647	1	*Araha.4647s0019*	59744	61612
A. halleri	Scaffold7562	-1	*Araha.7562s0009*	51999	53878
A. thaliana	Chr1	-1	*AT1G51420*	19064768	19066704
A. thaliana	Chr2	1	*AT2G35840*	15053634	15057426
A. thaliana	Chr3	1	*AT3G52340*	19407023	19409338
A. thaliana	Chr3	-1	*AT3G54270*	20087082	20089846
B. stricta	Scaffold697	-1	*Bostr.0697s0039*	207265	210482
B. stricta	Scaffold23794	-1	*Bostr.23794s0511*	3009918	3012614
B. stricta	Scaffold23997	-1	*Bostr.23997s0002*	2613	7221
B. stricta	Scaffold26675	1	*Bostr.26675s0053*	349141	351608
B. stricta	Scaffold6864	-1	*Bostr.6864s0011*	95609	97849
B. distachyon	Bd1	-1	*Bradi1g55300*	53990258	53994728
B. distachyon	Bd2	-1	*Bradi2g36350*	36715138	36718177
B. rapa	A03	1	*Brara.C01797*	8802831	8805021
B. rapa	A04	1	*Brara.D00476*	3533457	3536234
B. rapa	A04	-1	*Brara.D00597*	4668069	4670896
B. rapa	A04	1	*Brara.D02169*	17650448	17653037
B. rapa	A05	1	*Brara.E01630*	11061501	11064164
B. rapa	A08	-1	*Brara.H00238*	2142593	2145001
B. rapa	A09	-1	*Brara.I03697*	34284871	34287940
C. grandiflora	Scaffold562	-1	*Cagra.0562s0018*	53647	56465
C. grandiflora	Scaffold993	1	*Cagra.0993s0097*	329777	331957
C. grandiflora	Scaffold1889	1	*Cagra.1889s0022*	79975	82406
C. grandiflora	Scaffold2675	-1	*Cagra.2675s0012*	33460	35345
C. rubella	scaffold_1	-1	*Carubv10009241m.g*	17805133	17807030
C. rubella	scaffold_5	1	*Carubv10017320m.g*	9489857	9492282
C. rubella	scaffold_5	-1	*Carubv10019050m.g*	10262808	10265374
C. rubella	scaffold_4	1	*Carubv10023659m.g*	9990601	9993211
M. esculenta	scaffold02892	-1	*cassava4.1 008177m.g*	1185966	1189281
M. esculenta	scaffold08542	1	*cassava4.1 008254m.g*	559790	564944
M. esculenta	scaffold02369	1	*cassava4.1 011609m.g*	6475	10158
C. clementina	scaffold_6	1	*Ciclev10011822m.g*	19586148	19590349
C. clementina	scaffold_2	-1	*Ciclev10015425m.g*	645817	651088
C. reinhardtii	chromosome_6	-1	*Cre06.g283400*	5334229	5342395
C. reinhardtii	chromosome_7	-1	*Cre07.g348200*	5148450	5153785
C. reinhardtii	chromosome_9	1	*Cre09.g391578*	3798623	3805147
C. reinhardtii	scaffold_36	-1	*Cre36.g759647*	6598	14435
C. sativus	scaffold02047	-1	*Cucsa.242180*	1958113	1961168
C. sativus	scaffold02653	1	*Cucsa.282890*	188363	190842
C. subellipsoideaC_169	scaffold_11	-1	*e gw1.11.242.1*	619019	620900
C. subellipsoideaC_169	scaffold_7	-1	*e gw1.7.231.1*	632515	634251
E. grandis	Chr01	1	*Eucgr.A02547*	40693504	40699597
E. grandis	Chr07	-1	*Eucgr.G02659*	47155403	47160344
O. lucimarinus	Chr_1	-1	*eugene.0100010304*	510769	511710

Supplementary Table S4. Genome-wide identification of *SPP* genes in 50 sequenced genomes (to be continued)

Organism Name	Chromosome Name	Strand	Gene Name	Gene Start (bp)	Gene End (bp)
MspRCC299	Chr_03	1	*EuGene.0300010828*	1610180	1612123
C. papaya	supercontig_36	1	*evm.TU.supercontig 36.95*	742114	745805
A. trichopoda	AmTr_v1.0_scaffold00004	1	*evm_27.TU.AmTr_v1.0_scaffold00004.165*	2389854	2397072
C. subellipsoideaC_169	scaffold_2	-1	*fgenesh1 pg.2 # 381*	2655759	2659046
F. vesca	LG7	-1	*gene00357-v1.0-hybrid*	12511540	12517077
F. vesca	LG6	1	*gene03841-v1.0-hybrid*	33961580	33964468
F. vesca	LG6	1	*gene04408-v1.0-hybrid*	33519118	33521969
F. vesca	LG6	1	*gene04429-v1.0-hybrid*	33608935	33615883
F. vesca	LG6	1	*gene18142-v1.0-hybrid*	17922411	17927782
G. max	Chr10	-1	*Glyma.10G086600*	11135206	11138752
G. max	Chr10	1	*Glyma.10G120100*	30587542	30590466
G. max	Chr20	1	*Glyma.20G070500*	25094038	25100033
G. raimondii	Chr09	-1	*Gorai.009G301900*	27056548	27062424
G. raimondii	Chr09	-1	*Gorai.009G395300*	54676810	54680949
G. raimondii	Chr13	1	*Gorai.013G247000*	56583581	56585261
Z. mays	8	-1	*GRMZM2G055489*	115832258	115838487
Z. mays	10	1	*GRMZM2G097641*	89431021	89434142
V. vinifera	chr8	-1	*GSVIVG01022530001*	4430524	4436947
V. vinifera	chr12	-1	*GSVIVG01029753001*	14184316	14190383
V. vinifera	chr18	-1	*GSVIVG01034793001*	21736489	21737241
O. sativa	Chr1	-1	*LOC Os01g27880*	15568776	15573377
O. sativa	Chr2	1	*LOC Os02g05030*	2388399	2394346
O. sativa	Chr5	1	*LOC Os05g05270*	2595449	2599141
L. usitatissimum	scaffold1721	1	*Lus10005654.g*	235917	236957
L. usitatissimum	scaffold779	-1	*Lus10006183.g*	186247	187135
L. usitatissimum	scaffold273	-1	*Lus10012284.g*	377302	383463
L. usitatissimum	scaffold172	1	*Lus10015995.g*	159511	161525
M. domestica	MDC004679.177	-1	*MDP0000170391*	3224	11624
M. domestica	MDC011611.355	1	*MDP0000198686*	23000	25596
M. domestica	MDC008459.175	1	*MDP0000369360*	12592	14220
M. domestica	MDC018929.163	1	*MDP0000570741*	22	1737
M. truncatula	chr1	-1	*Medtr1g040560*	18584386	18589557
M. truncatula	chr1	1	*Medtr1g054980*	24163058	24167529
M. pusillaCCMP1545	scaffold_17	1	*MicpuC2.EuGene.0000170278*	490800	491980
M. guttatus	scaffold_10	1	*Migut.J00731*	4333528	4337761
C. sinensis	scaffold00018	-1	*orange1.1g014638m.g*	995594	999826
C. sinensis	scaffold00264	-1	*orange1.1g040434m.g*	149086	149614
C. sinensis	scaffold00264	-1	*orange1.1g048221m.g*	148089	148920
P. hallii	scaffold_86	-1	*Pahal.0086s0054*	331975	334927
P. hallii	scaffold_86	-1	*Pahal.0086s0055*	335278	338742
P. virgatum	Chr03a	-1	*Pavir.Ca01200*	13570228	13572843
P. virgatum	Chr03a	-1	*Pavir.Ca01201*	13573447	13576521
P. virgatum	Chr05a	-1	*Pavir.Ea01377*	20261658	20268495
P. virgatum	Chr05a	1	*Pavir.Ea03934*	61995345	61996200
P. virgatum	Chr05b	1	*Pavir.Eb01336*	24384136	24388282
P. virgatum	Chr08a	-1	*Pavir.Ha01118*	30542792	30544092
P. virgatum	contig00021	1	*Pavir.J00020*	18258	21287
P. virgatum	contig00021	1	*Pavir.J00021*	21789	25208
P. virgatum	contig00962	1	*Pavir.J00900*	4	1442
P. virgatum	contig04353	1	*Pavir.J03384*	376	1828
S. tuberosum	chr01	-1	*PGSC0003DMG400021341*	2091494	2096245
S. tuberosum	chr10	-1	*PGSC0003DMG400028134*	49108926	49113021
P. patens	Chr10	1	*Phpat.010G036800*	6346005	6349772
P. patens	Chr14	-1	*Phpat.014G019400*	4003434	4008314
P. patens	Chr19	-1	*Phpat.019G021900*	3646836	3649476
P. patens	Chr22	1	*Phpat.022G006700*	1242854	1247224
P. patens	Chr24	-1	*Phpat.024G003900*	917229	921247
P. vulgaris	Chr07	1	*Phvul.007G233700*	47379706	47384798

Supplementary Table S4. Genome-wide identification of *SPP* genes in 50 sequenced genomes (to be continued)

Organism Name	Chromosome Name	Strand	Gene Name	Gene Start (bp)	Gene End (bp)
P. vulgaris	Chr07	1	*Phvul.007G265500*	50318509	50322293
P. trichocarpa	Chr06	1	*Potri.006G199800*	21496115	21501036
P. trichocarpa	Chr08	1	*Potri.008G013300*	722452	726238
P. trichocarpa	Chr16	1	*Potri.016G066100*	4663719	4669374
P. persica	scaffold_5	-1	*ppa006104m.g*	314133	318252
P. persica	scaffold_7	-1	*ppa006143m.g*	12697894	12701833
S. purpurea	chr08	1	*SapurV1A.0070s0510*	504411	507061
S. purpurea	chr16	1	*SapurV1A.0258s0200*	4439281	4444488
S. purpurea	Scaffold0298	1	*SapurV1A.0298s0180*	148635	153658
S. italica	scaffold_5	1	*Si001574m.g*	18670426	18675673
S. italica	scaffold_3	1	*Si022142m.g*	3794773	3797199
S. italica	scaffold_3	1	*Si024709m.g*	3797804	3800147
S. italica	scaffold_2	1	*Si029938m.g*	45440722	45445169
S. bicolor	Chr04	1	*Sobic.004G151800*	47383945	47388587
S. bicolor	Chr09	1	*Sobic.009G040900*	3981857	3984167
S. bicolor	Chr09	1	*Sobic.009G041000*	3984705	3987521
S. lycopersicum	SL2.40ch01	-1	*Solyc01g006740.2*	1336414	1340710
S. lycopersicum	SL2.40ch10	1	*Solyc10g081660.1*	62014716	62018407
S. polyrhiza	pseudo0	1	*Spipo0G0172300*	10957792	10958754
S. polyrhiza	pseudo9	1	*Spipo9G0007600*	547544	548630
T. cacao	scaffold_5	-1	*Thecc1EG023137*	10799504	10802664
T. cacao	scaffold_10r	-1	*Thecc1EG044430*	16672866	16678360
E. salsugineum	scaffold_16	-1	*Thhalv10010361m.g*	1590047	1592525
E. salsugineum	scaffold_16	1	*Thhalv10010402m.g*	767979	771505
E. salsugineum	scaffold_7	1	*Thhalv10011514m.g*	2392922	2395116
E. salsugineum	scaffold_10	1	*Thhalv10016712m.g*	8372660	8375243
E. salsugineum	scaffold_8	1	*Thhalv10023476m.g*	1788773	1790912
T. aestivum	ta_iwgsc_1as_v1_282626	-1	*Traes 1AS 32F5EC9D2*	739	2224
T. aestivum	ta_iwgsc_1as_v1_3268565	-1	*Traes 1AS 49DFE6768*	65	1463
T. aestivum	ta_iwgsc_1bs_v1_3436665	1	*Traes 1BS 998F2E806*	1161	4630
T. aestivum	ta_iwgsc_1ds_v1_1897833	-1	*Traes 1DS 9AE5A76AC*	7413	10649
T. aestivum	ta_iwgsc_3dl_v1_6820059	-1	*Traes 3DL C9B8AE021*	1	704
T. aestivum	ta_iwgsc_5as_v1_1508999	1	*Traes 5AS 962BCA20C*	749	4732
T. aestivum	ta_iwgsc_5bs_v1_2269508	1	*Traes 5BS F7853DBB6*	2905	6449
T. aestivum	ta_iwgsc_5ds_v1_2744983	1	*Traes 5DS 0B17FFD55*	3838	10101
V. carteri	scaffold_2	-1	*Vocar20003060m.g*	469170	477667
V. carteri	scaffold_3	1	*Vocar20012795m.g*	3646250	3652593
V. carteri	scaffold_56	1	*Vocar20013715m.g*	244297	250858

Supplementary Table S5. Genome-wide identification of *UDPGP* genes in 50 sequenced genomes

Organism Name	Chromosome Name	Strand	Gene Name	Gene Start (bp)	Gene End (bp)
A. coerulea	scaffold_3	1	*Aquca_003_00207*	3362757	3369438
A. coerulea	scaffold_2	1	*Aquca_002_01330*	9612289	9619585
A. coerulea	scaffold_22	-1	*Aquca_022_00075*	951238	959707
A. coerulea	scaffold_35	1	*Aquca_035_00026*	146665	149970
A. coerulea	scaffold_14	1	*Aquca_014_00296*	1629183	1636715
A. coerulea	scaffold_20	1	*Aquca_020_00159*	1271863	1279046
A. coerulea	scaffold_3	-1	*Aquca_003_00223*	3571391	3576754
A. halleri	Scaffold10139	-1	*Araha.10139s0003*	6971	9942
A. halleri	Scaffold7513	-1	*Araha.7513s0018*	58277	63172
A. halleri	Scaffold58074	1	*Araha.58074s0001*	2860	6482
A. halleri	Scaffold783	1	*Araha.0783s0020*	80545	84731
A. halleri	Scaffold11841	-1	*Araha.11841s0002*	2758	6356
A. halleri	Scaffold1615	1	*Araha.1615s0014*	62714	66772
A. lyrata	scaffold_3	-1	*477571*	970343	975517
A. lyrata	scaffold_5	-1	*486043*	17776716	17781850
A. lyrata	scaffold_6	-1	*941238*	7132078	7135939
A. lyrata	scaffold_1	1	*473349*	14173729	14182253
A. lyrata	scaffold_8	1	*495327*	15209088	15213362
A. lyrata	scaffold_4	1	*482467*	16628111	16632043
A. thaliana	Chr5	1	*AT5G52560*	21330639	21334811
A. thaliana	Chr3	-1	*AT3G03250*	749390	754159
A. thaliana	Chr3	-1	*AT3G56040*	20792477	20797572
A. thaliana	Chr2	1	*AT2G35020*	14756711	14760706
A. thaliana	Chr1	1	*AT1G31070*	11084859	11088563
A. thaliana	Chr5	-1	*AT5G17310*	5696645	5700922
A. trichopoda	AmTr_v1.0_scaffold00032	-1	*evm_27.TU.AmTr_v1.0_scaffold00032.274*	5477737	5502367
A. trichopoda	AmTr_v1.0_scaffold00044	-1	*evm_27.TU.AmTr_v1.0_scaffold00044.42*	745718	757212
A. trichopoda	AmTr_v1.0_scaffold00202	-1	*evm_27.TU.AmTr_v1.0_scaffold00202.7*	115969	149235
B. distachyon	Bd4	1	*Bradi4g37350*	42621359	42626544
B. distachyon	Bd1	-1	*Bradi1g34180*	29876936	29885675
B. distachyon	Bd3	-1	*Bradi3g01640*	1013856	1018453
B. distachyon	Bd3	1	*Bradi3g17870*	16245571	16250027
B. distachyon	Bd5	-1	*Bradi5g21650*	24308392	24312427
B. rapa	A08	-1	*Brara.H00732*	9531421	9535280
B. rapa	A09	1	*Brara.I04426*	38537311	38541591
B. rapa	A09	-1	*Brara.I02753*	26407332	26411215
B. rapa	A05	1	*Brara.E03526*	27797985	27802592
B. rapa	A03	-1	*Brara.C00762*	3487910	3492270
B. rapa	A01	1	*Brara.A03833*	30357812	30362008
B. rapa	A05	-1	*Brara.E00932*	5404730	5408545
B. stricta	Scaffold2618	-1	*Bostr.2618s0067*	334913	339364
B. stricta	Scaffold20505	1	*Bostr.20505s0150*	642079	647359
B. stricta	Scaffold19046	-1	*Bostr.19046s0036*	133900	139079
B. stricta	Scaffold7305	-1	*Bostr.7305s0014*	87122	91714
B. stricta	Scaffold23794	-1	*Bostr.23794s0601*	3419617	3423710
B. stricta	Scaffold10040	-1	*Bostr.10040s0309*	2856335	2857710
B. stricta	Scaffold15697	1	*Bostr.15697s0411*	2657178	2661128
C. clementina	scaffold_3	1	*Ciclev10023278m.g*	16688557	16695010
C. clementina	scaffold_5	1	*Ciclev10000592m.g*	37876854	37884032
C. clementina	scaffold_6	1	*Ciclev10011657m.g*	21464335	21470434
C. clementina	scaffold_2	1	*Ciclev10014997m.g*	29933202	29940646
C. grandiflora	Scaffold1508	-1	*Cagra.1508s0086*	359843	363449
C. grandiflora	Scaffold4849	1	*Cagra.4849s0003*	8000	12467
C. grandiflora	Scaffold993	1	*Cagra.0993s0012*	29129	33268
C. grandiflora	Scaffold2098	1	*Cagra.2098s0061*	216971	221789
C. grandiflora	Scaffold84	1	*Cagra.0084s0005*	8489	12985

Supplementary Table S5. Genome-wide identification of *UDPGP* genes in 50 sequenced genomes (to be continued)

Organism Name	Chromosome Name	Strand	Gene Name	Gene Start (bp)	Gene End (bp)
C. papaya	supercontig_178	1	*evm.TU.supercontig_178.21*	176409	185535
C. papaya	supercontig_17	1	*evm.TU.supercontig_17.110*	1422231	1432703
C. papaya	supercontig_64	1	*evm.TU.supercontig_64.151*	1095012	1103073
C. papaya	supercontig_30	1	*evm.TU.supercontig_30.136*	1812959	1820194
C. reinhardtii	chromosome_4	1	*Cre04.g229700*	3807638	3814436
C. reinhardtii	chromosome_14	-1	*Cre14.g621751*	2024150	2034535
C. reinhardtii	chromosome_7	-1	*Cre07.g345300*	4740540	4747565
C. rubella	scaffold_8	-1	*Carubv10027818m.g*	561829	564657
C. rubella	scaffold_8	1	*Carubv10026051m.g*	7381797	7385980
C. rubella	scaffold_6	-1	*Carubv10000883m.g*	5737529	5741826
C. rubella	scaffold_5	-1	*Carubv10018646m.g*	11014775	11019338
C. rubella	scaffold_3	-1	*Carubv10013624m.g*	770375	775267
C. rubella	scaffold_1	1	*Carubv10011519m.g*	10839946	10843298
C. rubella	scaffold_4	1	*Carubv10024537m.g*	9686525	9690226
C. sativus	scaffold00542	1	*Cucsa.048760*	3398531	3405715
C. sativus	scaffold03746	-1	*Cucsa.375300*	232872	243934
C. sativus	scaffold03159	-1	*Cucsa.328110*	412152	422436
C. sativus	scaffold01044	-1	*Cucsa.136760*	1349309	1361599
C. sativus	scaffold04100	1	*Cucsa.393970*	70010	76838
C. sinensis	scaffold00021	1	*orange1.1g002690m.g*	245944	252289
C. sinensis	scaffold00376	1	*orange1.1g006541m.g*	117669	124478
C. sinensis	scaffold00091	1	*orange1.1g012172m.g*	138623	145733
C. sinensis	scaffold00008	1	*orange1.1g010950m.g*	1418861	1427055
C. sinensis	scaffold00004	-1	*orange1.1g006297m.g*	81941	89007
C. subellipsoideaC_169	scaffold_10	1	*estExt_fgenesh1_pm.C_100138*	1549458	1554318
C. subellipsoideaC_169	scaffold_10	-1	*estExt_fgenesh1_pm.C_100179*	1840627	1844450
C. subellipsoideaC_169	scaffold_2	-1	*fgenesh1_pm.2_#_281*	3114381	3119561
E. grandis	Chr04	-1	*Eucgr.D01117*	23599880	23607017
E. grandis	Chr06	1	*Eucgr.F03856*	49282945	49292511
E. grandis	Chr10	1	*Eucgr.J00183*	1914873	1923553
E. grandis	Chr05	-1	*Eucgr.E04308*	75560271	75568189
E. grandis	Chr06	-1	*Eucgr.F02905*	41206358	41212892
E. salsugineum	scaffold_10	1	*Thhalv10016553m.g*	8023222	8027308
E. salsugineum	scaffold_5	-1	*Thhalv10007394m.g*	3802695	3806548
E. salsugineum	scaffold_2	-1	*Thhalv10013449m.g*	5831580	5836899
E. salsugineum	scaffold_15	1	*Thhalv10000457m.g*	6367076	6370519
E. salsugineum	scaffold_13	1	*Thhalv10020665m.g*	8716037	8720732
F. vesca	LG1	1	*gene31637-v1.0-hybrid*	10318924	10325100
F. vesca	LG3	-1	*gene03208-v1.0-hybrid*	10690636	10698050
F. vesca	LG7	-1	*gene21010-v1.0-hybrid*	17573691	17579427
F. vesca	LG2	1	*gene10960-v1.0-hybrid*	17240904	17247438
F. vesca	LG3	1	*gene19994-v1.0-hybrid*	7435268	7441173
G. max	Chr18	1	*Glyma.18G044400*	3836270	3837813
G. max	Chr05	-1	*Glyma.05G132100*	32506962	32515601
G. max	Chr04	-1	*Glyma.04G245100*	51257284	51265241
G. max	Chr18	1	*Glyma.18G025500*	1879117	1887759
G. max	Chr11	-1	*Glyma.11G231700*	32734340	32742549
G. max	Chr05	-1	*Glyma.05G164500*	35525530	35532374
G. max	Chr08	-1	*Glyma.08G121900*	9389955	9396364
G. max	Chr14	1	*Glyma.14G210700*	47577189	47584929
G. max	Chr02	1	*Glyma.02G241100*	42951620	42957971
G. max	Chr06	1	*Glyma.06G118100*	9616298	9624675
G. max	Chr13	1	*Glyma.13G152500*	26712715	26718689

Supplementary Table S5. Genome-wide identification of *UDPGP* genes in 50 sequenced genomes (to be continued)

Organism Name	Chromosome Name	Strand	Gene Name	Gene Start (bp)	Gene End (bp)
G. max	Chr19	1	*Glyma.19G104700*	35430695	35431194
G. max	scaffold_675	-1	*Glyma.U041600*	33	3370
G. max	Chr06	-1	*Glyma.06G001900*	173301	187071
G. max	Chr08	-1	*Glyma.08G086700*	6555198	6564711
G. raimondii	Chr01	1	*Gorai.001G152100*	21237082	21239688
G. raimondii	Chr04	1	*Gorai.004G048300*	4354037	4361011
G. raimondii	Chr11	-1	*Gorai.011G201100*	48722807	48723759
G. raimondii	Chr07	-1	*Gorai.007G250600*	39407018	39413564
G. raimondii	Chr11	1	*Gorai.011G181500*	43037560	43039588
G. raimondii	Chr06	-1	*Gorai.006G047500*	15537793	15539785
G. raimondii	Chr08	1	*Gorai.008G132300*	37865349	37868355
G. raimondii	Chr05	1	*Gorai.005G063100*	6638951	6641558
G. raimondii	Chr04	1	*Gorai.004G254500*	59153528	59159026
G. raimondii	Chr13	1	*Gorai.013G108400*	23671668	23679521
G. raimondii	Chr11	1	*Gorai.011G291300*	62257481	62263043
G. raimondii	Chr09	1	*Gorai.009G415300*	63894616	63894950
G. raimondii	Chr07	-1	*Gorai.007G188400*	18148823	18154007
G. raimondii	Chr11	-1	*Gorai.011G040700*	3016906	3022448
G. raimondii	Chr11	-1	*Gorai.011G102900*	11808854	11809255
G. raimondii	Chr02	-1	*Gorai.002G159200*	34883252	34887026
G. raimondii	Chr03	-1	*Gorai.003G092100*	27806451	27806848
G. raimondii	Chr08	1	*Gorai.008G049800*	7040053	7045522
G. raimondii	Chr05	-1	*Gorai.005G268300*	64020019	64025371
G. raimondii	Chr05	-1	*Gorai.005G087200*	11202160	11211348
L. usitatissimum	scaffold27	1	*Lus10036227.g*	273003	277682
L. usitatissimum	scaffold286	1	*Lus10010957.g*	339055	343092
L. usitatissimum	scaffold123	1	*Lus10041971.g*	174210	180211
L. usitatissimum	scaffold302	1	*Lus10007370.g*	198924	207423
L. usitatissimum	scaffold1519	-1	*Lus10007512.g*	196755	201008
L. usitatissimum	scaffold346	-1	*Lus10028658.g*	647527	653112
L. usitatissimum	scaffold28	1	*Lus10038366.g*	948186	952612
L. usitatissimum	scaffold511	1	*Lus10017443.g*	647641	651895
L. usitatissimum	scaffold2968	1	*Lus10000971.g*	39539	44231
L. usitatissimum	scaffold303	1	*Lus10020788.g*	695113	699336
L. usitatissimum	scaffold863	-1	*Lus10031365.g*	52794	56944
M. domestica	MDC004836.412	-1	*MDP0000323036*	13329	19054
M. domestica	MDC020292.85	1	*MDP0000232533*	21020	27047
M. domestica	MDC010439.1078	1	*MDP0000828130*	5574	6034
M. domestica	MDC009987.257	-1	*MDP0000187925*	8238	12654
M. domestica	MDC020292.81	1	*MDP0000475073*	3876	17151
M. domestica	MDC002134.125	-1	*MDP0000841490*	14754	19471
M. domestica	MDC018800.123	-1	*MDP0000156131*	31057	38609
M. domestica	MDC001961.200	1	*MDP0000794656*	1827	4950
M. domestica	MDC000641.134	1	*MDP0000263801*	5487	7211
M. domestica	MDC018876.225	1	*MDP0000141108*	1629	4773
M. domestica	MDC010439.1077	1	*MDP0000191267*	644	6492
M. domestica	MDC013034.68	-1	*MDP0000292934*	5173	11180
M. domestica	MDC022696.164	-1	*MDP0000739295*	2465	5620
M. domestica	MDC012290.160	-1	*MDP0000300177*	22737	36583
M. esculenta	scaffold07520	-1	*cassava4.1_006979m.g*	420055	425860
M. esculenta	scaffold12147	-1	*cassava4.1_006282m.g*	112150	123283
M. esculenta	scaffold10689	1	*cassava4.1_001216m.g*	433183	441339
M. esculenta	scaffold07591	1	*cassava4.1_006973m.g*	36284	43714
M. esculenta	scaffold11297	-1	*cassava4.1_003947m.g*	856994	863521
M. guttatus	scaffold_13	-1	*Migut.M01998*	21087868	21092430
M. guttatus	scaffold_3	-1	*Migut.C01079*	17304868	17310642
M. guttatus	scaffold_2	1	*Migut.B01416*	16199290	16206078
M. guttatus	scaffold_11	-1	*Migut.K00740*	5743063	5748700

Supplementary Table S5. Genome-wide identification of *UDPGP* genes in 50 sequenced genomes (to be continued)

Organism Name	Chromosome Name	Strand	Gene Name	Gene Start (bp)	Gene End (bp)
M. pusillaCCMP1545	scaffold_9	-1	*estExt_Genewise1Plus.C_9_t20186*	702950	708262
M. pusillaCCMP1545	scaffold_10	-1	*MicpuC2.estExt_fgenesh1_pg.C 100296*	1093648	1095677
M. spRCC299	Chr_06	-1	*estExt_fgenesh2_pm.C_Chr_060089*	674913	677776
M. spRCC299	Chr_12	1	*e_gw2.12.183.1*	4219	5784
M. truncatula	chr4	-1	*Medtr4g094668*	38685187	38693786
M. truncatula	chr5	1	*Medtr5g099590*	43623390	43625231
M. truncatula	chr3	1	*Medtr3g088650*	40382946	40391125
M. truncatula	chr3	-1	*Medtr3g064480*	29049928	29052183
M. truncatula	chr5	1	*Medtr5g077000*	32848157	32856006
M. truncatula	chr3	-1	*Medtr3g064490*	29053045	29057091
M. truncatula	chr3	1	*Medtr3g118200*	55329045	55338497
O. lucimarinus	Chr_21	1	*estExt_Genewise_ext.C_Chr 210146*	202897	204870
O. lucimarinus	Chr_13	-1	*estExt_Genewise_ext.C_Chr 130208*	323599	325572
O. lucimarinus	Chr_11	1	*eugene.1100010317*	564302	565765
O. sativa	Chr1	1	*LOC_Os01g15910*	8958411	8967207
O. sativa	Chr4	-1	*LOC_Os04g52370*	31108920	31113473
O. sativa	Chr2	-1	*LOC_Os02g02560*	926031	929945
O. sativa	Chr6	-1	*LOC_Os06g48760*	29508824	29515309
O. sativa	Chr8	1	*LOC_Os08g10600*	6237449	6242123
O. sativa	Chr9	1	*LOC_Os09g38030*	21917319	21922425
P. hallii	scaffold_151	-1	*Pahal.0151s0049*	383304	387626
P. hallii	scaffold_134	-1	*Pahal.0134s0123*	729594	735132
P. hallii	scaffold_35	-1	*Pahal.0035s0067*	504967	508911
P. hallii	scaffold_32	-1	*Pahal.0032s0200*	1818474	1822873
P. hallii	scaffold_43	-1	*Pahal.0043s0013*	70169	77826
P. hallii	scaffold_31	-1	*Pahal.0031s0243*	1539283	1547210
P. patens	Chr07	-1	*Phpat.007G086600*	15624597	15628084
P. patens	Chr11	-1	*Phpat.011G002400*	295341	300987
P. patens	Chr15	-1	*Phpat.015G054900*	9379878	9384278
P. patens	Chr23	1	*Phpat.023G072800*	13417946	13422968
P. patens	Chr04	1	*Phpat.004G065000*	11874343	11879296
P. patens	Chr14	1	*Phpat.014G102200*	16781685	16787294
P. patens	Chr01	-1	*Phpat.001G001100*	94210	99511
P. persica	scaffold_2	-1	*ppa001281m.g*	23106483	23112607
P. persica	scaffold_1	-1	*ppa003010m.g*	39711770	39720258
P. persica	scaffold_4	-1	*ppa004903m.g*	6203915	6210952
P. persica	scaffold_2	-1	*ppa006111m.g*	13716838	13719028
P. persica	scaffold_2	-1	*ppa026418m.g*	13711797	13713020
P. persica	scaffold_3	1	*ppa005232m.g*	208225	215962
P. trichocarpa	Chr01	1	*Potri.001G353800*	36152636	36160024
P. trichocarpa	Chr04	1	*Potri.004G074400*	6213982	6220184
P. trichocarpa	Chr02	-1	*Potri.002G077400*	5347369	5353765
P. trichocarpa	Chr17	-1	*Potri.017G144700*	15254831	15260627
P. trichocarpa	Chr04	1	*Potri.004G074600*	6227395	6230876
P. trichocarpa	Chr01	-1	*Potri.001G159700*	13297689	13303709
P. trichocarpa	Chr03	1	*Potri.003G074700*	10333095	10339996
P. trichocarpa	Chr10	1	*Potri.010G185800*	18245315	18253570
P. trichocarpa	Chr13	1	*Potri.013G070000*	5812970	5820453
P. virgatum	contig01442	1	*Pavir.J01317*	5	5606
P. virgatum	contig102406	-1	*Pavir.J06411*	4	2966
P. virgatum	Chr01b	-1	*Pavir.Ab00183*	2505526	2509812
P. virgatum	Chr04b	1	*Pavir.Db00243*	3461846	3467304
P. virgatum	Chr02a	1	*Pavir.Ba01388*	17646178	17651649
P. virgatum	Chr06a	1	*Pavir.Fa01746*	41889296	41893859

Supplementary Table S5. Genome-wide identification of *UDPGP* genes in 50 sequenced genomes (to be continued)

Organism Name	Chromosome Name	Strand	Gene Name	Gene Start (bp)	Gene End (bp)
P. virgatum	Chr07a	1	*Pavir.Ga00467*	5402196	5406365
P. virgatum	Chr02b	1	*Pavir.Bb02554*	59189330	59191675
P. virgatum	contig14638	1	*Pavir.J12326*	3381	7912
P. virgatum	contig03163	-1	*Pavir.J02665*	6573	20316
P. virgatum	contig153859	-1	*Pavir.J13229*	1044	2779
P. virgatum	contig193167	1	*Pavir.J17527*	937	2251
P. virgatum	Chr01a	1	*Pavir.Aa03505*	70493164	70497548
P. virgatum	contig12516	1	*Pavir.J09594*	2	7880
P. vulgaris	Chr02	1	*Phvul.002G190700*	34670116	34681615
P. vulgaris	Chr09	1	*Phvul.009G130500*	19201311	19209056
P. vulgaris	Chr09	-1	*Phvul.009G117800*	17567058	17576084
P. vulgaris	Chr08	-1	*Phvul.008G240600*	55496379	55503818
R. communis	30148	1	*30148.t000004*	43156	50990
R. communis	30171	1	*30171.t000020*	320489	327017
R. communis	29648	-1	*29648.t000056*	400064	407584
R. communis	27704	-1	*27704.t000009*	66308	71188
R. communis	29814	-1	*29814.t000031*	207551	216275
S. bicolor	Chr06	-1	*Sobic.006G213100*	57099905	57104389
S. bicolor	Chr04	-1	*Sobic.004G013500*	1124939	1129671
S. bicolor	Chr02	1	*Sobic.002G291200*	66908714	66915334
S. bicolor	Chr07	1	*Sobic.007G075500*	8439367	8444474
S. bicolor	Chr10	-1	*Sobic.010G251200*	58837788	58855545
S. italica	scaffold_6	-1	*Si013631m.g*	1950114	1954400
S. italica	scaffold_2	1	*Si029679m.g*	39477356	39482964
S. italica	scaffold_1	1	*Si017115m.g*	9865008	9869083
S. italica	scaffold_7	1	*Si012193m.g*	34815330	34821097
S. italica	scaffold_3	-1	*Si024886m.g*	16487876	16495327
S. italica	scaffold_7	-1	*Si010650m.g*	29765321	29767831
S. italica	scaffold_4	1	*Si005943m.g*	36636773	36644855
S. lycopersicum	SL2.40ch11	-1	*Solyc11g011960.1*	4912805	4919067
S. lycopersicum	SL2.40ch02	1	*Solyc02g068530.2*	33060678	33069001
S. lycopersicum	SL2.40ch05	-1	*Solyc05g054060.2*	63182024	63189388
S. lycopersicum	SL2.40ch04	1	*Solyc04g058070.2*	54328964	54342003
S. lycopersicum	SL2.40ch01	-1	*Solyc01g081520.2*	73222596	73233236
S. lycopersicum	SL2.40ch06	1	*Solyc06g051080.2*	30687110	30697786
S. moellendorffii	scaffold_6	-1	*167999*	3735916	3738260
S. moellendorffii	scaffold_23	1	*100378*	531037	533716
S. moellendorffii	scaffold_3	-1	*165987*	2240143	2242680
S. polyrhiza	pseudo8	-1	*Spipo8G0011100*	1035083	1050528
S. polyrhiza	pseudo17	1	*Spipo17G0034300*	2589312	2593501
S. polyrhiza	pseudo7	-1	*Spipo7G0027800*	2497810	2506955
S. polyrhiza	pseudo16	-1	*Spipo16G0026600*	1921679	1926136
S. purpurea	Scaffold4037	1	*SapurV1A.4037s0010*	8648	10744
S. purpurea	chr01	1	*SapurV1A.0210s0260*	13513809	13517930
S. purpurea	chr01	1	*SapurV1A.0273s0260*	12341257	12350453
S. purpurea	chr04	1	*SapurV1A.0147s0470*	8591356	8600346
S. purpurea	chr04	1	*SapurV1A.0147s0450*	8602293	8602724
S. purpurea	chr17	1	*SapurV1A.0392s0040*	17296403	17299762
S. purpurea	Scaffold1526	-1	*SapurV1A.1526s0020*	13743	21688
S. purpurea	Scaffold0829	-1	*SapurV1A.0829s0050*	19935	27469
S. purpurea	chr16	1	*SapurV1A.0126s0130*	22257788	22264844
S. purpurea	chr01	1	*SapurV1A.0210s0250*	13518207	13526284
S. purpurea	chr13	-1	*SapurV1A.0671s0120*	9398109	9406324
S. purpurea	chr03	-1	*SapurV1A.0104s0260*	5729730	5736373
S. tuberosum	chr04	-1	*PGSC0003DMG400023024*	47955755	47968998
S. tuberosum	chr02	1	*PGSC0003DMG400010448*	48850860	48859924

Supplementary Table S5. Genome-wide identification of *UDPGP* genes in 50 sequenced genomes (to be continued)

Organism Name	Chromosome Name	Strand	Gene Name	Gene Start (bp)	Gene End (bp)
S. tuberosum	chr01	1	*PGSC0003DMG401031123*	73395793	73405106
S. tuberosum	chr11	1	*PGSC0003DMG401013333*	2402051	2408593
T. aestivum	ta_iwgsc_1bl_v1_3809624	1	*Traes_1BL_5C7C06485*	1645	4332
T. aestivum	ta_iwgsc_7bl_v1_6741135	1	*Traes_7BL_4C45F5D62*	565	7219
T. aestivum	ta_iwgsc_6as_v1_4340916	1	*Traes_6AS_3A8E07254*	2129	5676
T. aestivum	ta_iwgsc_6bs_v1_2504270	1	*Traes_6BS_0AFE47E4B*	1	3357
T. aestivum	ta_iwgsc_1dl_v1_2274275	-1	*Traes_1DL_65E1F2414*	8807	13363
T. aestivum	ta_iwgsc_1al_v2_3923578	1	*Traes_1AL_5AD4E3431*	456	1948
T. aestivum	ta_iwgsc_5al_v1_2769163	1	*Traes_5AL_E97939490*	27	5060
T. aestivum	ta_iwgsc_7dl_v1_3354930	-1	*Traes_7DL_6CCFD4346*	4401	11757
T. aestivum	ta_iwgsc_2al_v1_6379266	-1	*Traes_2AL_81025812B*	10031	14213
T. aestivum	ta_iwgsc_7bl_v1_6739348	1	*Traes_7BL_AB31FFA84*	30	1199
T. aestivum	ta_iwgsc_6bs_v1_332886	-1	*Traes_6BS_9B66808AE*	1	251
T. aestivum	ta_iwgsc_7al_v1_4378343	1	*Traes_7AL_8CDD7A174*	344	6937
T. aestivum	ta_iwgsc_2dl_v1_9908928	-1	*Traes_2DL_DAAE7AFAF*	1	4022
T. aestivum	ta_iwgsc_2bl_v1_8033683	-1	*Traes_2BL_965768E2D*	6349	9087
T. aestivum	ta_iwgsc_5bl_v1_10822911	1	*Traes_5BL_AEEB6621B*	8627	14540
T. aestivum	ta_iwgsc_5dl_v1_4567798	1	*Traes_5DL_CFFABFAA6*	4752	9926
T. cacao	scaffold_8	1	*Thecc1EG035220*	7174026	7181604
T. cacao	scaffold_4	1	*Thecc1EG016775*	109696	117963
T. cacao	scaffold_10r	1	*Thecc1EG042464*	646984	652705
T. cacao	scaffold_2	1	*Thecc1EG006018*	311663	317452
T. cacao	scaffold_5	-1	*Thecc1EG025753*	34428171	34434800
V. carteri	scaffold_11	1	*Vocar20005448m.g*	134598	140538
V. carteri	scaffold_1	1	*Vocar20009812m.g*	13542252	13547795
V. carteri	scaffold_12	-1	*Vocar20006051m.g*	2871233	2887893
V. carteri	scaffold_4	-1	*Vocar20010534m.g*	1392048	1402804
V. vinifera	chr1	1	*GSVIVG01012124001*	1226337	1240986
V. vinifera	chr4	1	*GSVIVG01026563001*	21784491	21800688
V. vinifera	chr18	-1	*GSVIVG01008799001*	2203013	2218030
V. vinifera	chr13	-1	*GSVIVG01032777001*	824557	834472
Z. mays	7	-1	*GRMZM2G402650*	117340728	117345980
Z. mays	5	-1	*GRMZM2G098370*	70071104	70073937
Z. mays	4	1	*GRMZM2G342226*	14112401	14120324
Z. mays	3	1	*GRMZM2G018022*	152617469	152622382
Z. mays	4	-1	*GRMZM2G519736*	147663687	147668178
Z. mays	1	1	*AC195366.3_FG008*	292407905	292410550
Z. mays	10	-1	*GRMZM2G155729*	63796931	63801064
Z. mays	2	1	*GRMZM2G044629*	10478639	10483070
Z. mays	2	1	*GRMZM6G729818*	198410457	198414756
Z. mays	5	-1	*GRMZM2G116586*	56811276	56822670
Z. mays	8	1	*GRMZM2G378402*	129654037	129658984
Z. mays	9	-1	*GRMZM2G424038*	5945755	5946378
Z. mays	10	-1	*GRMZM2G019986*	141495963	141500441
Z. mays	6	1	*GRMZM2G161493*	86936812	86947218
Z. mays	5	1	*GRMZM2G134415*	14242164	14246423
Z. mays	7	-1	*GRMZM2G032003*	146528998	146534984
Z. mays	7	-1	*AC216869.3_FG001*	12405146	12407775

Supplementary Table S6. Distribution of *SuSy*, *SPS*, *SPP* and *UDPGP* gene families

Classification	SuSy	SPS	SPP	UDPGP
Higher plant	Yes	Yes	Yes	Yes
Moss	Yes	Yes	Yes	Yes
Algae	Yes	No	Yes	Yes
Animal	No	No	No	Yes
Fungi	No	No	Yes	Yes
Bacteria	Yes	Yes	Yes	Yes
Archaea	Yes	No	Yes	No
Virus	No	No	No	No
